# Suitable Evaluation Frameworks for Disease-Agnostic Platforms for Remote Patient Monitoring: Scoping Review

**DOI:** 10.2196/68910

**Published:** 2025-06-16

**Authors:** Arrash Yassaee, Angus Bruno Reed, Ben Swanson, Ana Luísa Neves, Dougal Hargreaves

**Affiliations:** 1 School of Public Health Imperial College London London United Kingdom; 2 Huma Therapeutics London United Kingdom; 3 Medical Sciences Division University of Oxford Oxford United Kingdom; 4 Department of Primary Care and Public Health School of Public Health Imperial College London London United Kingdom; 5 Mohn Centre for Children's Health & Wellbeing School of Public Health Imperial College London London United Kingdom

**Keywords:** digital health, remote patient monitoring, mobile health, mHealth, medical device regulation

## Abstract

**Background:**

Disease-agnostic platforms (DAPs) are a digital health intervention (DHI) category that can support patients across multiple clinical conditions. While their versatility and configurability can address the fragmentation caused by condition-specific DHIs, DAPs present challenges for evaluation and certification, as they must be assessed across multiple therapeutic areas and diverse applications. A core challenge is identifying suitable evaluation frameworks that can accommodate the highly adaptable nature of this technology.

**Objective:**

This review explored whether there are suitable evaluation frameworks for the appraisal and subsequent certification of DAPs, whether any were applied in previous evaluations of DHIs and were available through open access (OA) publications.

**Methods:**

Twelve databases (PubMed, Embase, PsycINFO, MEDLINE, Scopus, Web of Science, ACMDL, IEEE Xplore, CINAHL, Cochrane Library, Compendex, and Business Source Complete) were searched on January 28, 2024. Titles, abstracts, and full texts were screened by 1 reviewer, with a minimum 10% sample dual-screened. Inclusion criteria included describing an evaluation framework applied to a DHI. Studies were excluded if there was no novel evaluation framework available to researchers, applicable to digital health evaluation domains, and applicable to a broad patient population. Evaluation frameworks identified were combined with those identified from 5 previous reviews, alongside handsearching and gray literature results. Each framework was assessed against essential and desirable criteria. Essential criteria were applicability across different therapeutic areas and populations, applicability to all domains of digital health, ability to support both formative and summative evaluation, and availability to researchers. Frameworks that met all essential criteria were assessed against desirable criteria, which included presence of operational guidance, evidence of considerations specific to digital health, and incorporation of an evidence assessment method.

**Results:**

A total of 40,907 search results contained 72 new frameworks. These were combined with 181 frameworks identified from previous reviews and 1 framework from handsearching and gray literature. Of the 254 frameworks assessed, 15 met all essential criteria, indicating potential suitability for DAP evaluation. One framework, the WHO (World Health Organization) guideline on monitoring and evaluating DHIs, met all desirable criteria. All suitable frameworks had been applied to DHIs and were available in at least 1 OA publication.

**Conclusions:**

We found that at least 15 existing frameworks appear suitable for DAP evaluation and can be used to benchmark DAPs against other DHIs. Limitations include potential bias from screening methods and underpinning reviews. Prior use of these frameworks in DHI evaluations and their availability in OA publications enables standardized, high-quality evaluation. Identification of suitable frameworks indicates that research efforts can focus on other outstanding questions related to DAP evaluation, including the acceptable performance benchmark for DAPs, the range of therapeutic areas DAPs need to be favorably evaluated in, and how to synthesize findings across multiple therapeutic areas.

**Trial Registration:**

OSF Registries 10.17605/OSF.IO/X578S; https://osf.io/x578s

## Introduction

### Background

In the digital age, mobile health (mHealth) has emerged as a subtype of telemedicine, focusing on the use of technologies, including mobile phones and wearables (including smart watches), to collect health data and support medical and public health practice [1].

In particular, smartphone-based remote patient monitoring (RPM) is a form of mHealth that facilitates care decisions outside of traditional hospital settings through regular data capture and faster decision-making [2]. Across all modalities of RPM, many of which use more traditional technologies such as phone calls and SMS, smartphone RPM is the largest and fastest growing. The global RPM market and supporting device market are multibillion sectors, both projected to be 12-figure industries by 2030, with annual growth rates of ~20% [3]. Smartphone-based RPM takes advantage of the growing prevalence of smartphones to deploy readily available software for remote monitoring in care and research settings. While the concept of mHealth has existed since 2003, recent advancements in hardware and software, coupled with the ubiquity of handset ownership, have now made smartphone RPM feasible at scale [4]. Additionally, significant investment in digital health following the COVID-19 pandemic has seen transformations that have enabled increased uptake in wider digital health services pre-pandemic, including updated user interfaces, increased digital literacy, and realignment of financial and policy incentives [5,6]. Accordingly, recent research indicates that smartphone RPM is an acceptable and feasible modality of care delivery across a range of therapeutic areas [7]. Additionally, early evidence synthesis indicates that it may be a particularly promising modality for pediatric telemedicine [8].

However, one persisting pain point is a fragmented digital ecosystem, a problem observed for decades [9]. Most digital health interventions (DHIs) are point solutions with a narrow focus, for example, supporting care in a single condition. Where DHIs have an intended use that covers multiple conditions, this tends to focus on a specific component of the care journey, such as symptom checkers or medication diaries [10,11]. Although the dominating paradigm, DHIs, with a narrow intended use, do not reflect the reality of multimorbidity, nor that health care providers offer care pathways across a range of therapeutic areas. As a result, providers and patients often use up to 5-10 different non-integrated point solutions to meet their health and wellness needs [12].

### Approaches to Date

Efforts to reduce this fragmentation have often centered around software integration. While standard rules and specifications, such as the Fast Healthcare Interoperability Resources standards by Health Level-7 UK, exist and are commonly adopted by DHIs, there is still significant variation in implementation [13]. As a result, several promising approaches to end-stage integration, such as the use of application programming interfaces, have not delivered the seamless experience originally touted [14].

A more recent, alternative approach to reducing fragmentation has been to introduce full-pathway standardization through the development of disease-agnostic platform (DAP) solutions. These are software solutions that, from the outset, are deliberately designed to cover a range of scenarios and disease areas. In the example of RPM, monitoring for a population of patients with a range of different chronic diseases can either be addressed through a series of point solutions, each tailored for a specific disease (eg, a focused asthma solution and standalone diabetes solutions), or through a single DAP, which can cater for the totality of a patient’s needs across their health journey, as well as covering the additional range of therapeutic areas serviced by providers. Several such technologies already exist, have seen hundreds of millions of dollars of investment, and are starting to influence national guidelines [15].

### Current Challenges

Platform technologies are increasingly used in industries including commerce, finance, and quality management. Applying this technology in health care has additional hurdles, including certification and regulation. DHIs that serve a medical purpose are designated as Software as Medical Devices (SaMDs) and must follow a series of legal requirements, regulatory guidelines, and best practices. This includes the need to use an appropriate evaluation framework and the requirement to compare the performance of an SaMD against current state-of-the-art (SoTA) [16,17].

Compared to other DHIs, DAPs have additional considerations that need to be accounted for during evaluation. For example, DAPs cover multiple therapeutic areas, meaning appropriate evaluation frameworks must be able to accommodate and synthesize findings from different clinical endpoints. Additionally, DAPs are designed to be configured and tailored for a range of use cases. Formative evaluations can help refine configurations of DAPs and support selection of metrics to measure device performance for subsequent summative evaluation [18]. Therefore, appropriate evaluation frameworks must support both formative and summative evaluations as said configurations are tested and scaled [19].

Despite a growing library of DHI evaluation frameworks, recent reviews have found many frameworks to be insufficient to meet the needs of regulators and key stakeholders such as payers, professional organizations, and clinicians [20,21]. This suggests that when evaluating new technologies such as DAPs, researchers cannot assume that current frameworks are appropriate for certification and stakeholder assurance. To date, there has been no framework specifically identified as suitable for DAPs. This has contributed to the reason why certification of SaMD platforms can be highly heterogenous, making comparisons and benchmarking challenging.

However, should a suitable evaluation framework be identified, this would offer a route to standardizing evaluation and certification of DAPs. Furthermore, if a review of the literature found that a suitable framework already exists, is readily accessible to researchers, and has already been used to appraise DHIs, this would facilitate better comparisons between DAPs and point solutions, enabling benchmarking of DAPs against current SoTA. Compared to hand searching and expert recommendations, a broad review of the literature offers a better chance of identifying suitable frameworks and may identify multiple options to choose from.

### Goal of This Review

The aim of this scoping review is to identify if DAP evaluations can feasibly be conducted by one or more existing evaluation frameworks. Specific objectives include the following: (1) to identify and describe existing evaluation frameworks suitable for the appraisal of DAPs; (2) to evaluate whether the frameworks identified in (1) have previously been applied to DHIs; and (3) to evaluate whether any frameworks identified in (1) were open access (OA).

## Methods

This scoping review followed the PRISMA-ScR (Preferred Reporting Items for Systematic Reviews and Meta-Analyses extension for Scoping Reviews) checklist (Multimedia Appendix 1) [22]. The study protocol was published on Open Science Framework before full-text screening commenced [23]. There were no deviations from this protocol.

### Search Strategy

Evaluation frameworks were identified using three complementary approaches: (1) evaluation frameworks identified by 5 prior reviews of evaluation frameworks (Kowatsch et al; Lagan et al; Moshi et al; Nouri et al; and Silberman et al) [20,21,24-26]; (2) refreshing the searches conducted by Kowatsch et al; Lagan et al; Moshi et al; and Nouri et al to include records between when the original search was conducted and January 2024; and (3) searching gray literature sources, including OpenGrey, Mobile Active and ProQuest Dissertation and Theses, and handsearching of journals.

This approach was chosen to build upon and complement the work of previous research on digital health evaluation, thereby leveraging existing knowledge and validated search strategies [20,21,24-26]. Recognizing that reviews often built upon each other's search strategies [20,21], we aimed to consolidate and extend the existing body of work to ensure a comprehensive and up-to-date survey of evaluation frameworks to help address our specific research question. Previous attempts to consolidate and refresh existing reviews have adopted scoping methods [20] with a limited breadth of databases used, potentially explaining why international digital health evaluation guidance was not captured.

By drawing upon previous research, updating and expanding validated searches, and incorporating gray literature, this approach aimed to provide the most robust identification of frameworks to maximize the chances of identifying frameworks suitable for DAP evaluation.

This approach enabled prior screening efforts to be leveraged, reducing duplication of previous work. Additionally, the multi-search approach circumvented the significant challenge, identified during consultations with a specialist librarian, of creating a single new search strategy that was neither too restrictive to miss relevant frameworks nor yielding unmanageable numbers of results.

As part of (2), 12 databases (PubMed, Embase, PsycInfo, MEDLINE, Scopus, Web of Science, ACM Digital Library, IEEE Xplore, CINAHL, Cochrane Library, Compendex, and Business Source Complete) were searched on January 28, 2024. In each instance, the search concepts and terms used by the original reviews were replicated, with no language limits. The date limit set to start from the corresponding reviews’ search was executed and ended on January 28, 2024. Although this approach to date restriction resulted in searches with different date restrictions, this was done to reduce overlap and redundancy with the original reviews. Examples of search terms are outlined in Multimedia Appendix 2.

#### Study Selection Criteria

Studies were included based on the below criteria (Textbox 1).

Studies were not excluded on the basis of design; therefore, commentaries and opinion pieces that cited novel, relevant frameworks were included. Similarly, studies were not excluded on the basis of geography or study participants. However, studies were excluded based on the following criteria (Textbox 2).

**Study inclusion criteria.**

- They cited an evaluation frameworks used to appraise a digital health tool that had a diagnostic and therapeutic purpose.
- Addressed clinical efficacy or effectiveness in addition to other assessment domains (eg, usability and patient acceptance).
- When assessing the quality of study design, the study had to use an established evidence assessment method.

**Study exclusion criteria.**

- They only discussed evaluation frameworks already identified by one of the 5 previous reviews.
- From the description, it was evident that the evaluation framework could only be applied to a specific therapeutic area or patient population (eg, relied on a specific biomarker or safety signal).
- From the description, it was evident that the framework was not available to researchers (eg, proprietary tools that required license fees) or included usability, clinical effectiveness, and health economics assessment [27].
- From the description, it was evident that the framework is not applicable to any of the domains in digital health evaluation.
- The study described a bespoke evaluation without framing the methodology as an approach that could be applied to other digital health interventions.

#### Screening and Data Extraction

All searches were conducted on January 28, 2024, with results uploaded into EndNote (version 21.2) for initial deduplication and then to Covidence for further deduplication and screening.

All abstracts and full papers were screened by a single reviewer, and, at each stage, a second reviewer double-screened 10% while blinded to the first reviewer’s decisions. Following this, interrater reliability (IRR) was calculated using Cohen κ. If IRR <0.81 (the threshold for near-perfect agreement [28]), a further 10% would be double-screened. This continued until IRR was >0.80 or all results were screened. In line with the protocol, all disagreements were reconciled with discussion, with the use of a third arbitrating screener not required.

Studies that passed full-text screening then underwent data extraction. Data extraction was conducted using a standardized data extraction form using Covidence, with fields including the name of frameworks mentioned in the paper, the original citation of the frameworks, the year of publication of the frameworks, as well as free text notes.

One researcher undertook manual extraction of all included results. In parallel, a second researcher undertook an extraction of a 10% sample. They were compared, and in the event of material differences, these would be reconciled by consensus, and a further 10% would be extracted. Minor differences (eg, formatting and order of extracting frameworks) were not considered meaningful and were interpreted as extractor agreement.

These identified frameworks were then combined with the frameworks identified through components (1) and (3) of the overall review strategy.

All identified evaluation frameworks were assessed against pre-agreed criteria. These are outlined below (Table 1) along with a justification for inclusion. Frameworks were initially appraised on criteria i-iv, which were viewed as “essential” for DAPs. Frameworks that met all 4 criteria were then appraised on criteria v-x, which were viewed as “desirable” criteria. Frameworks were judged as potentially suitable for DAP evaluation if they met all essential criteria.

Due to the high number (150+) of frameworks anticipated, a pragmatic approach to synthesis was adopted, mirroring the methods used for screening. A single researcher appraised all frameworks against the essential criteria. A random 10% sample was then appraised by a second reviewer.

**Table 1:** Assessment criteria for evaluation frameworks.

Criterion	Detail	Essential or desirable	Justification
i	Applicability not restricted to a specific population and therapeutic area	Essential	Frameworks that cannot be applied to multiple therapeutic areas are implicitly unsuitable to appraise DAPs^a^ [29].
ii	Can be applied to all domains of digital health evaluation (including clinical efficacy and effectiveness, usability, health economic assessment, etc)	Essential	The evaluation of DHIs^b^ should evolve alongside the intervention's maturity, encompassing different domains as it scales [30]. DAPs will have specific configurations at different deployment maturities. This necessitates a framework applicable across all domains to support evaluation throughout the DAP lifecycle.
iii	Can support formative and summative evaluation (eg, not limited to a retrospective checklist)	Essential	DAPs are designed to be regularly (re)configured for a range of use cases. Identifying and improving suitable configurations requires a combination of formative and summative evaluation [18].
iv	Freely available to researchers and is not a proprietary tool that requires license fees or similar.	Essential	Standardized approaches to evaluation are impractical if resources are inaccessible and evaluators have a financial disincentive to using the framework [31].
v	References evidence considerations that are specific to digital health (eg, how to approach blinding in DHI evaluations, reporting intention-to-treat analyses, ensuring the study population reflects the target population, etc [20]).	Desirable	Desirable criteria to improve researcher awareness on how to ensure evaluations are appropriately tailored to the unique nature of digital interventions [30]. Deemed not essential, as researchers trained in digital health evaluation should still be able to apply the framework appropriately, potentially using external guidance.
vi	Applied to digital health previously	Desirable	Desirable criteria indicate the framework has practical utility. Deemed not essential as the framework’s suitability should depend on intrinsic characteristics (eg, scope, flexibility, methods, etc)
vii	Available in at least 1 resource in English	Desirable	Desirable criteria ensure the framework is comprehensible to a large segment of the research and innovation community. Deemed not essential as language affects reach, not core suitability.
viii	Flexible enough to incorporate other scoring tools or evaluation checklists	Desirable	Desirable criteria, as this would facilitate benchmarking against existing published evaluations of technologies. Deemed not essential as frameworks can still be self-sufficient for DAP evaluation without this benchmarking.
ix	Incorporates an evidence assessment method (eg, GRADE^c^)	Desirable	Desirable criteria, as this facilitates appraisal and comparison of evidence across a range of study designs. Viewed as a useful feature, as RCTs^d^ may not always be practical or appropriate for digital health evaluation [30]. Deemed not essential, as such methods can be applied separately.
x	Provides guidance and advice on how to conduct and design digital health evaluation	Desirable	Desirable criteria to guide evaluators and encourage standardization of digital health evaluation [32]. Deemed not essential, as an absence of guidance would still enable suitably experienced researchers to use the framework.

^a^DAP: disease-agnostic platform.

^b^DHI: digital health intervention.

^c^GRADE: Grading of Recommendations Assessment, Development, and Evaluation.

^d^RCT: randomized controlled trial.

#### Risk of Bias Assessment

Given the focus on extracting evaluation frameworks rather than empirical findings, risk of bias, reporting bias, and certainty assessments were not deemed applicable. Instead, frameworks were evaluated based on their performance against preagreed criteria.

#### Data Synthesis

A narrative synthesis of identified frameworks was performed to analyze and summarize the extracted data, exploring the performance of all studies against the 4 essential criteria, starting with the most frequently met criterion. Frameworks meeting all 4 essential criteria and thus potentially suitable for DAP evaluation were then discussed. Given the focus of the research was on identifying suitable frameworks, a meta-analysis was not appropriate.

## Results

### Search Results

**Figure 1:**
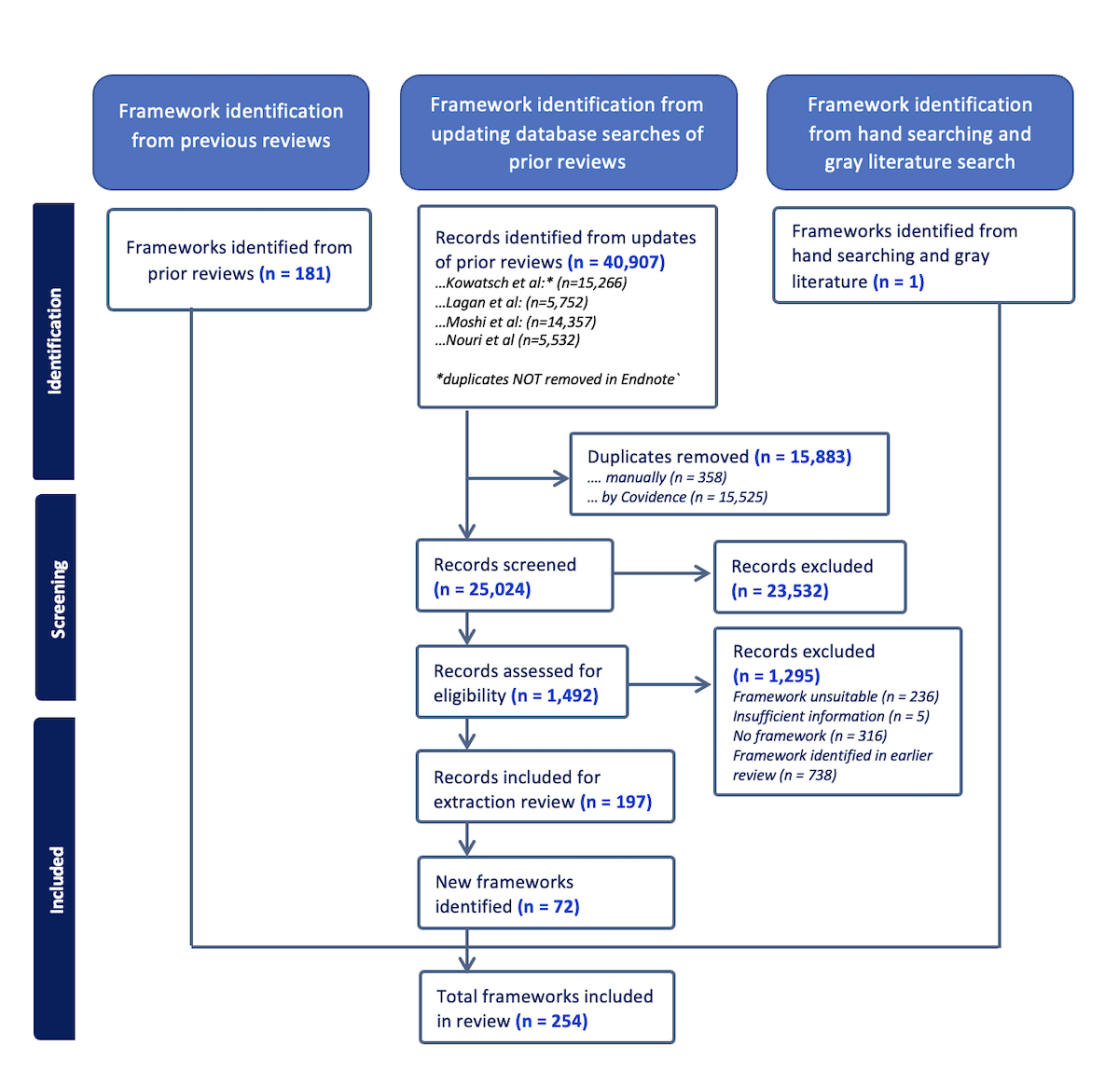
PRISMA (Preferred Reporting Items for Systematic Reviews and Meta-Analyses) flowchart of this review.

Initial searches retrieved a total of 40,907 results. For both abstract and full-text screening, only 1 round of double-screening was required to achieve an IRR of >0.80. After title, abstract, and full-text screening, 197 papers met the inclusion criteria and were extracted (Multimedia Appendix 3). Across these 197 papers, 72 new frameworks were described. These new frameworks were combined with the 181 frameworks found in previous reviews and 1 framework identified from hand searching and gray literature, resulting in a total of 254 frameworks for evaluation (Figure 1 [21,24-26]).

#### Evaluation of Identified Frameworks

The 254 identified frameworks were initially evaluated against the 4 essential criteria (i-iv). Publication dates of frameworks ranged from 1994 to 2024, with more than half of frameworks published in 2016 or later. A full evaluation of the 254 frameworks against the essential criteria can be found in Multimedia Appendix 4.

Availability to researchers was the most met criterion, satisfied by 239 out of 254 (94.1%) frameworks. Reasons why frameworks failed this criterion included requiring a license fee or an appointment with the framework author, being replaced by new standards, or the resource no longer being available. In some instances, only an abridged version of the framework [33], or results of assessments using the framework, was freely available [34].

Similarly, 213 (83.8%) frameworks could be applied to different populations and therapeutic areas. Of the frameworks where applicability was limited to a specific indication, the focus tended to be diabetes, mental health, and infectious disease (including HIV). Reasons for this included assessment of specific biomarkers or presence of specific clinical signs and symptoms that indicate poor clinical outcomes [35,36].

Fewer than a third (29.9%) of frameworks were applicable across all domains of digital health evaluation. While there were some instances where frameworks solely focused on 1 domain (for example, clinical effectiveness), in most instances frameworks were unable to support one or more evaluation domains, typically usability and health economic assessment [37,38].

Most frameworks were designed to support retrospective appraisal, with only 22 (8.7%) frameworks conducive to formative and summative evaluation. Of the frameworks which met this criterion, 15 (68.2%) met the other 3 essential criteria. Those that did not were limited by the digital health domains they could cover, for example, deriving from a narrow focus of intervention or target population [39,40].

Addressing the primary research question, 15 frameworks met all 4 essential criteria and thus were potentially suitable for DAP evaluation. These frameworks were subsequently appraised against criteria v-x, summarized below (Table 2).

There were 3 desirable criteria met by all 15 frameworks—previously being applied to digital health, availability in English, and flexibility to incorporate other checklists and scores.

Practical guidance for conducting digital health evaluations was offered by 6 frameworks. Similarly, there was a range of the detail and length of this guidance. For example, the World Health Organization (WHO) guide extends to over 100 pages, with distinct sections on ideating, conducting, and reporting evaluations. In contrast, the Design and Evaluation of Digital Health Interventions framework provided just over 600 words of high-level guidance on how to conduct evaluations across 4 phases.

Only 3 frameworks referenced evidence considerations specific to digital health evaluation, while only 2 frameworks included an evidence appraisal method, useful for platforms whose validation will rely on multiple evaluations across different use-cases. Of the suitable frameworks, only 1 met all desirable criteria—the WHO guideline on monitoring and evaluating DHIs.

**Table 2:** Assessment of frameworks against evaluation criteria.

Evaluation framework and year of development	Criteria									
	i	ii	iii	iv	v	vi	vii	viii	ix	x
Digital therapeutics alliance “Setting the Stage” recommendations [41], 2022	Y^a^	Y	Y	Y	Y	Y	Y	Y	N^b^	Y
Design and evaluation of digital health interventions (DEDHI) [21], 2019	Y	Y	Y	Y	N	Y	Y	Y	N	Y
Framework for the effectiveness evaluation of mobile (mental) health tools, MindTech Healthcare Cooperative, National Institute for Health Research [42], 2017	Y	Y	Y	Y	N	Y	Y	Y	Y	Y
MRC^c^ complex intervention framework [43], 2000	Y	Y	Y	Y	N	Y	Y	Y	N	Y
Revised MRC complex intervention framework [44], 2008	Y	Y	Y	Y	N	Y	Y	Y	N	Y
Multiphase Optimization Strategy (MOST) [45], 2007	Y	Y	Y	Y	N	Y	Y	Y	N	N
6 steps in quality intervention development (6SQuID) [46], 2016	Y	Y	Y	Y	N	Y	Y	Y	N	N
Reach effectiveness adoption implementation and maintenance (RE-AIM) framework [47], 1999	Y	Y	Y	Y	N	Y	Y	Y	N	N
Consolidated framework for implementation research (CFIR) [48], 2009	Y	Y	Y	Y	N	Y	Y	Y	N	N
ADDIE (analysis, design, development, implementation, and evaluation) [49], 1975	Y	Y	Y	Y	N	Y	Y	Y	N	N
Trial of intervention principles framework [50], 2015	Y	Y	Y	Y	Y	Y	Y	Y	N	N
WHO^d^ guideline on monitoring and evaluating DHIs [51], 2016	Y	Y	Y	Y	Y	Y	Y	Y	Y	Y
Clinical adoption framework (CAF) [52], 2011	Y	Y	Y	Y	N	Y	Y	Y	N	N
Iterative decision-making for evaluation of adaptations (IDEA) [53], 2020	Y	Y	Y	Y	N	Y	Y	Y	N	N
Sequential Multiple Assignment Randomized Trial (SMART) [45], 2007	Y	Y	Y	Y	N	Y	Y	Y	N	N

^a^Y: yes.

^b^N: no.

^c^MRC: Medical Research Council.

^d^WHO: World Health Organization.

#### Previous Application of Frameworks in Digital Health

Addressing one of the secondary research questions, all 15 frameworks had previously been applied to digital health. A forward citation review on Google Scholar revealed a range in citation frequency, with frameworks such as MOST (Multiphase Optimization Strategy), SMART (Sequential Multiple Assignment Randomized Trial), and that by the Medical Research Council cited over a thousand times, compared to 18 citations for the clinical adoption framework paper by Lau and Price [52].

#### Open Access Availability

All 15 suitable frameworks were available through at least 1 OA publication, addressing the other secondary research question.

## Discussion

### Principal Results

Across the 254 frameworks included in this review, 15 (5.9%) were deemed suitable for the evaluation and subsequent certification of DAPs in digital health, all of which had been used for previous digital health evaluations and all of which were available in at least 1 OA publication.

The most common reason frameworks were unsuitable for DAP evaluation was their inability to support both formative and summative evaluation, a key requirement for the iterative nature of platform deployment. This highlights a potential gap in the current DHI evaluation framework literature. Most frameworks appear to be designed for static solutions, for the benefit of external appraisers rather than to support manufacturers, and often for DHIs with narrow rather than broad intended uses.

### Interpretation of Findings in the Context of Previous Research

These findings are consistent with previous reviews which highlight the limitations of existing DHI evaluation frameworks, particularly their struggle to keep pace with the specific requirements of digital health technologies [20,21,24-26]. These reviews often point to the limitations of traditional, often rigid, evaluation models derived from drug development paradigms when applied to the dynamic and iterative nature of DHIs.

However, this review specifically addresses the emerging need for frameworks suitable for DAPs, a rapidly evolving area in digital health that presents unique evaluation challenges. Unlike point solutions designed for specific conditions, DAPs require frameworks capable of equitably assessing their adaptability and effectiveness across multiple therapeutic areas and diverse user populations. The identification of 15 suitable frameworks provides a valuable resource for researchers and industry professionals working in this space. It enables the benchmarking of DAPs against current SoTA solutions, particularly in therapeutic areas with a high volume of DHIs, such as diabetes and asthma, where benchmarks and clinical guidelines are better established and can serve as points of reference.

The review also highlighted the large number of DHI evaluation frameworks developed in recent years. This proliferation partly stems from a growing recognition that traditional evaluation practices in health care are based on drug development paradigms. Key differences that DHI evaluation frameworks need to accommodate include the appropriateness of control arms in digital health, acknowledgement of rapid development cycles, and co-creation with intended users [30,50].

This review highlights the value of frameworks originating from outside of the digital health domain, fields such as quality improvement, which offer different perspectives on evaluating complex interventions within real-world settings. Nonetheless, the review did identify that frameworks designed primarily for digital health evaluations were more likely to reference specific evidence considerations in digital health—a common gap identified in previous literature [20,41,51].

The varying genesis of frameworks also explains the spectrum of prescriptiveness across these frameworks. For example, frameworks such as MOST and SMART focus on randomized controlled trials [45]. In contrast, the WHO framework offers guidance on how to synthesize evidence across a variety of study designs [51]. This flexibility is of value given that randomized controlled trials are still a debated study design in digital health [30,50,54].

Frameworks with a genesis in digital health were also more likely to offer detailed guidance on ideating and conducting evaluations at different stages of an intervention’s maturity. This is another consideration in DAPs where multiple deployments may exist simultaneously at different scales and implementation stages.

Some standardization is desirable if digital health evaluations are to be comparable and benchmarks of DHI performance are to be determined. Where frameworks offer minimal implementation guidance to research, authors and other researchers have acknowledged that there remain unanswered questions and challenges regarding the operationalization of a framework [46,47,55].

Among the identified frameworks, the WHO guidance appears particularly suitable for DAP evaluation. The detailed guidance around conducting evaluation, as well as specific considerations unique to digital health, places the framework in a good position to facilitate consistently high-quality evaluation. Furthermore, the framework presents an approach to synthesizing evidence across multiple evaluations, which would be particularly useful for DAPs targeting deployment in multiple pathways simultaneously. The framework is also cited extensively in digital health evaluations.

### Strengths and Limitations

The review benefits from a comprehensive search strategy, increasing the likelihood of identifying relevant frameworks. The search strategy covered 12 databases and gray literature sources and combined terms validated from previous published reviews. It is reassuring that the majority of suitable frameworks arose from refreshed searches and that some frameworks were hosted in gray literature but were still picked up by our strategy. The goal of the review was not to recommend a specific framework but to assess whether an existing framework could be used to appraise DAP or whether a novel framework would need to be created. On this aspect, we believe this review has provided sufficient evidence that, for now, existing approaches can suffice.

However, several limitations should be considered. First, the appraisal of frameworks was based on a priori assessment of the evaluation needs of DAPs. The essential criteria were selected to be as minimally restrictive as possible and so were limited to those criteria without which appraisal of DAPs would be critically flawed. As this type of technology becomes more common and stakeholders more comfortable in its appraisal, additional or modified criteria may emerge which could be applied in a future refresh of this review. For example, there may be greater emphasis on standardizing evaluations in digital health to enable stakeholders to directly compare technologies. In this scenario, providing explicit guidance on evidence considerations specific to digital health might be viewed as an essential aspect when appraising evaluation frameworks.

Secondly, our exclusion criteria, while necessary for focus, might have inadvertently excluded frameworks that could be adapted or relevant for DAP evaluation under different contexts. For example, standalone evaluations that did not cite a formal framework may have adopted a method suitable for DAPs. Additionally, our strategy deliberately built upon 5 previous reviews to leverage existing work; however, this reliance could introduce inherited biases from the scope or conclusions of those original reviews, potentially influencing the pool of frameworks identified.

Thirdly, the review did not formally evaluate the practicality or ease of application of the identified frameworks, which could influence their uptake and utility for researchers and developers. Usability and real-world applicability could be a focus in future research; however, it is reassuring that all identified frameworks have been cited already, sometimes extensively. We also did not formally evaluate the specific challenges or practicalities of applying these frameworks within the context of regulatory certification for SaMDs. We recommend this is a focus for future work, starting with whether any of these frameworks can support the determination of SoTA for DAPs.

Additionally, the pragmatic approach to dual screening may have introduced some bias, and there may be additional frameworks screened out that would not have been had all results been dual screened. However, given the sheer number of results from the searches, a pragmatic approach to screening was required for this work to be feasible. Additionally, we are somewhat assured by the fact that the approach to dual screening had a similar sample size but a higher κ threshold than similar approaches in published the literature and is recognized as preferable to single reviewer screening [56,57].

### Implications for Research, Policy, and Practice

The review offers some implications for the development, evaluation, and regulation of DAPs.

### Benchmarking Frameworks

First, that there are frameworks that are suitable for DAP evaluations indicates that efforts should be directed towards benchmarking and appraising DAPs, rather than creating novel frameworks from scratch.

The prior use of existing frameworks for DHI evaluation provides academics, manufacturers, and procurers with a range of credible methodologies for benchmarking DAPs against the current SoTA. This is particularly pertinent when appraising DAP performance in therapeutic areas such as diabetes and asthma, where the digital health landscape is already quite crowded and where more standardized approaches would be of benefit to patients, providers, and health systems.

That all potentially suitable frameworks were available in at least 1 OA source also bodes well for the accessibility and utility of these frameworks in standardizing DHI assessment.

Using some of these identified frameworks to critically appraise the evidence base for specific DAPs can support policy makers and practitioners in the procurement of DAPs and the ongoing monitoring of their utility.

### Regulatory Implications

Additionally, the identification of appropriate evaluation frameworks offers regulators a route to appraising the conformity of DAPs with medical device regulations. Although some knowledge gaps remain, including the application of these frameworks towards certifying SaMDs, these can be addressed through a combination of agreed guidance and further research.

To illustrate, the feasibility of DAPs, a foundational aspect of many evaluation frameworks, will need to be determined for each therapeutic area. One approach could be to develop recommendations of minimum feature lists for DHIs in each therapeutic area. For example, a DAP cannot feasibly support asthma management (and hence be truly considered disease agnostic) if it is unable to keep a record of as-required inhaler use or track respiratory parameters such as peak flow, validated questionnaires, and oxygen saturations. Similarly, a DAP cannot legitimately claim to be disease agnostic if it cannot track blood sugar levels in a way to support the management of type 1 diabetes. An a priori approach, based on existing care guidelines across therapeutic areas and starting with the most common conditions currently supported by RPM, offers a practicable path to outlining necessary (but not sufficient) requirements for DAPs.

Related questions include how wide a range of therapeutic areas a DAP should be favorably evaluated in before it can be certified with a broad, condition-agnostic intended use. Researchers should also consider how to synthesize findings across multiple therapeutic areas to enable different DAPs to be meaningfully compared, considering the context-dependent nature of outcomes.

Medical device regulations require manufacturers to compare their product against current SoTA (ie, established practice) [58,59]. Although this review highlights frameworks that would be suitable to evaluate DAPs, it is currently unclear what the acceptable performance benchmark, and thereby SoTA, would be for this technology. Current medical device regulations see individual manufacturers conduct their own, internal SoTA analysis [60]. Published research that synthesizes evidence on this topic could improve transparency in DHI certification and could facilitate innovation by removing the burden from individual manufacturers.

### Future Work

Based on the frameworks identified in this review, a promising candidate for SoTA assessment in DAPs would be usability. This appears in a range of frameworks, would be a universal metric collected across therapeutic areas, and would be apparent in the early stages of DHI deployment, thereby acting as an early signal of quality.

As digital health expands in scope and expertise, newer frameworks may be developed, which may be superior compared to those identified in this review. Future refreshes of the search strategy, followed by additional or refined framework appraisal criteria—for example, complexity of application—may be of value. Considering the large number of records returned in the search, a pragmatic approach would be to refresh this review every 3-4 years to maintain its currency.

### Conclusions

In conclusion, this refreshed search of the literature, combined with previous reviews, identified several frameworks suitable for DAP evaluation, all of which have been applied in digital health previously and which are available through at least 1 OA publication. Frameworks such as the WHO guidelines show promise due to flexibility, specific tailoring for digital evaluation, and offering practical operational guidance.

This review has also identified priority topics for future research. This includes clarifying and defining the SoTA for RPM, with usability as one promising candidate evaluation domain for benchmarking DAP performance.

## Data Availability

The datasets generated or analyzed during this study are available from the corresponding author on reasonable request.

## Appendix

Multimedia Appendix 1 PRISMA-ScR checklist.

Multimedia Appendix 2 Examples of search terms used across databases.

Multimedia Appendix 3 Summarized extraction of included studies.

Multimedia Appendix 4 Appraisal of identified frameworks against essential criteria.

## Abbreviations

DAP: disease-agnostic platform
DHI: digital health intervention
IRR: interrater reliability
mHealth: mobile health
MOST: Multiphase Optimization Strategy
OA: open access
PRISMA-ScR: Preferred Reporting Items for Systematic Reviews and Meta-Analyses extension for Scoping Reviews
RPM: remote patient monitoring
SaMD: Software as Medical Device
SMART: Sequential Multiple Assignment Randomized Trial
SoTA: state-of-the-art
WHO: World Health Organization
