# Supplementary material for: Suitable Evaluation Frameworks for Disease-Agnostic Platforms for Remote Patient Monitoring: Scoping Review
Source: J Med Internet Res. 2025 Jun 16;27:e68910. doi: 10.2196/68910 (PMC12209728; doi:10.2196/68910)
Supplement: Multimedia Appendix 2 [file jmir_v27i1e68910_app2.docx]

Appendix 2 – Examples of search terms used across databases.

*Table 1 – information sources and interfaces used to refresh constituent reviews*

| **Original review** | **Database used** | **Interface employed** | **Date restriction** |
| --- | --- | --- | --- |
| *Lagan et al* | *Pubmed* | *Pubmed* | *01 Oct 2020 – 28 Jan 2024* |
|  | *Embase* | *Ovid* |  |
|  | *Psychinfo* | *Ovid* |  |
| *Kowatsch et al* | *Medline* | *Ovid* | *01 May 2019 – 28 Jan 2024* |
|  | *Embase* | *Ovid* |  |
|  | *Scopus* | *EBSCO* |  |
|  | *Web of Science* | *EBSCO* |  |
|  | *ACM-DL* | *EBSCO* |  |
|  | *IEEE Xplore* | *EBSCO* |  |
| *Moshi et al* | *Pubmed* | *Pubmed* | *01 Nov 2016 - 28 Jan 2024* |
|  | *Medline* | *Ovid* |  |
|  | *Psychinfo* | *Ovid* |  |
|  | *CINAHL* | *Ovid* |  |
|  | *Cochrane Library* | *Cochrane library* |  |
|  | *Compendex* | *EBSCO* |  |
|  | *Business Source Complete* | *EBSCO* |  |
| *Nouri et al* | *Medline* | *Ovid* | *23 Dec 2016 – 28 Jan 2024* |
|  | *Embase* | *Ovid* |  |
|  | *Scopus* | *EBSCO* |  |
|  | *Web of Science* | *EBSCO* |  |

**Appendix 1a: Searches to refresh the review conducted by Lagan et al [1]**

**Pubmed:**

((mobile application) OR (smartphone app)) AND ((framework) OR (criteria) OR (rating))

*Filter applied for results between 01 Oct 2020 and 28 January 2024*

**Embase:**

| **#** | **Query** |
| --- | --- |
| 1 | exp mobile application/ |
| 2 | smartphone app*.mp. |
| 3 | mobile app*.mp. |
| 4 | 1 or 2 or 3 |
| 5 | framework.mp. |
| 6 | criteria.mp. |
| 7 | rating.mp. |
| 8 | 5 or 6 or 7 |
| 9 | 4 and 8 |
| 10 | limit 9 to dc=20201001-20240128 |

**Psychinfo:**

| **#** | **Query** |
| --- | --- |
| 1 | exp mobile application/ |
| 2 | smartphone app*.mp. |
| 3 | mobile app*.mp. |
| 4 | 1 or 2 or 3 |
| 5 | framework.mp. |
| 6 | criteria.mp. |
| 7 | rating.mp. |
| 8 | exp Evaluation Criteria/ |
| 9 | exp Rating/ |
| 10 | 5 or 6 or 7 or 8 or 9 |
| 11 | 4 and 10 |
| 12 | limit 11 to up=20201001-20240128 |

**Appendix 1b: Searches to refresh the review conducted by Kowatsch et al [2]**

**Medline:**

| **#** | **Query** |
| --- | --- |
| 1 | assessment.tw. |
| 2 | assessing.tw. |
| 3 | evaluation.tw. |
| 4 | evaluating.tw. |
| 5 | criteria.tw. |
| 6 | rating.tw. |
| 7 | score.tw. |
| 8 | scoring.tw. |
| 9 | 1 or 2 or 3 or 4 or 5 or 6 or 7 or 8 |
| 10 | intervention.tw. |
| 11 | program.tw. |
| 12 | therapy.tw. |
| 13 | prevention.tw. |
| 14 | treatment.tw. |
| 15 | app.tw. |
| 16 | application.tw. |
| 17 | 10 or 11 or 12 or 13 or 14 or 15 or 16 |
| 18 | health.ti. |
| 19 | medical.ti. |
| 20 | clinic.ti. |
| 21 | clinical.ti. |
| 22 | 18 or 19 or 20 or 21 |
| 23 | digital.ti. |
| 24 | mobile.ti. |
| 25 | app.ti. |
| 26 | mobile application.ti. |
| 27 | mobile.ti. |
| 28 | web.ti. |
| 29 | internet.ti. |
| 30 | smartphone.ti. |
| 31 | phone.ti. |
| 32 | mobile-phone.ti. |
| 33 | electronic.ti. |
| 34 | mhealth.ti. |
| 35 | m-health.ti. |
| 36 | ehealth.ti. |
| 37 | e-health.ti. |
| 38 | telemedicine.ti. |
| 39 | tele-medicine.ti. |
| 40 | telehealth.ti. |
| 41 | tele-health.ti. |
| 42 | 23 or 24 or 25 or 26 or 27 or 28 or 29 or 30 or 31 or 32 or 33 or 34 or 35 or 36 or 37 or 38 or 39 or 40 or 41 |
| 43 | 9 and 17 and 22 and 42 |
| 44 | limit 43 to dt=20190501-20240128 |

**Embase:**

| **#** | **Query** |
| --- | --- |
| 1 | assessment.tw. |
| 2 | assessing.tw. |
| 3 | evaluation.tw. |
| 4 | evaluating.tw. |
| 5 | criteria.tw. |
| 6 | rating.tw. |
| 7 | score.tw. |
| 8 | scoring.tw. |
| 9 | 1 or 2 or 3 or 4 or 5 or 6 or 7 or 8 |
| 10 | intervention.tw. |
| 11 | program.tw. |
| 12 | therapy.tw. |
| 13 | prevention.tw. |
| 14 | treatment.tw. |
| 15 | app.tw. |
| 16 | application.tw. |
| 17 | 10 or 11 or 12 or 13 or 14 or 15 or 16 |
| 18 | health.ti. |
| 19 | medical.ti. |
| 20 | clinic.ti. |
| 21 | clinical.ti. |
| 22 | 18 or 19 or 20 or 21 |
| 23 | digital.ti. |
| 24 | mobile.ti. |
| 25 | app.ti. |
| 26 | mobile application.ti. |
| 27 | mobile.ti. |
| 28 | web.ti. |
| 29 | internet.ti. |
| 30 | smartphone.ti. |
| 31 | phone.ti. |
| 32 | mobile-phone.ti. |
| 33 | electronic.ti. |
| 34 | mhealth.ti. |
| 35 | m-health.ti. |
| 36 | ehealth.ti. |
| 37 | e-health.ti. |
| 38 | telemedicine.ti. |
| 39 | tele-medicine.ti. |
| 40 | telehealth.ti. |
| 41 | tele-health.ti. |
| 42 | 23 or 24 or 25 or 26 or 27 or 28 or 29 or 30 or 31 or 32 or 33 or 34 or 35 or 36 or 37 or 38 or 39 or 40 or 41 |
| 43 | 9 and 17 and 22 and 42 |
| 44 | limit 43 to dc=20190501-20240128 |

**Scopus:**

( TITLE-ABS-KEY ( assessment OR assessing OR evaluation OR evaluating OR criteria OR rating OR score OR scoring ) AND TITLE-ABS-KEY ( intervention OR program OR therapy OR prevention OR treatment OR app OR application ) AND TITLE ( health OR medical OR clinic OR clinical ) AND TITLE ( digital OR mobile OR app OR mobile AND application OR mobile AND app OR web OR internet OR smartphone OR phone OR mobile-phone OR electronic OR mhealth OR m-health OR ehealth OR e-health OR telemedicine OR tele-medicine OR telehealth OR tele-health ) ) AND PUBYEAR > 2018

**Web of Science:**

| 1 | ((TI=(assessment OR assessing OR evaluation OR evaluating OR criteria OR rating OR score OR scoring)) OR AB=(assessment OR assessing OR evaluation OR evaluating OR criteria OR rating OR score OR scoring)) |
| --- | --- |
| 2 | ((TI=(assessment OR program OR therapy OR prevention OR treatment OR app OR application)) OR AB=(assessment OR program OR therapy OR prevention OR treatment OR app OR application)) |
| 3 | (TI=(health OR medical OR clinic OR clinical) |
| 4 | (TI=(digital OR mobile OR app OR mobile application OR mobile app OR web OR internet OR smartphone OR phone OR mobile phone OR electronic OR mhealth OR m-health OR ehealth OR e-health OR telemedicine OR tele-medicine OR telehealth or tele-health)) |
| 5 | #1 AND #2 AND #3 AND #4 |

**ACM-DL:**

[[Title: assessment] OR [Title: assessing] OR [Title: evaluation] OR [Title: evaluating] OR [Title: criteria] OR [Title: rating] OR [Title: score] OR [Title: scoring] OR [Abstract: assessment] OR [Abstract: assessing] OR [Abstract: evaluation] OR [Abstract: evaluating] OR [Abstract: criteria] OR [Abstract: rating] OR [Abstract: score] OR [Abstract: scoring]]

AND [[Title: intervention] OR [Title: program] OR [Title: therapy] OR [Title: prevention] OR [Title: treatment] OR [Title: app] OR [Title: application] OR [Abstract: intervention] OR [Abstract: program] OR [Abstract: therapy] OR [Abstract: prevention] OR [Abstract: treatment] OR [Abstract: app] OR [Abstract: application]]

AND [[Title: health] OR [Title: medical] OR [Title: clinic] OR [Title: clinical]]

AND [[Title: digital] OR [Title: mobile] OR [Title: app] OR [Title: mobile application] OR [Title: mobile app] OR [Title: web] OR [Title: internet] OR [Title: smartphone] OR [Title: phone] OR [Title: mobile-phone] OR [Title: electronic] OR [Title: mhealth] OR [Title: m-health] OR [Title: ehealth] OR [Title: e-health] OR [Title: telemedicine] OR [Title: tele-medicine] OR [Title: telehealth] OR [Title: tele-health]]

AND [E-Publication Date: (01/05/2019 TO 28/01/2024)]

**IEE Explore:**

| 1 | (((((“Document Title”:digital OR “Document Title”:mobile OR “Document Title”:app OR “Document Title”:mobile application OR “Document Title”:mobile app OR “Document Title”:web OR “Document Title”:internet OR “Document Title”:smartphone OR “Document Title”:phone OR “Document Title”:mobile-phone OR “Document Title”:electronic OR “Document Title”:mhealth OR “Document Title”:m-health OR “Document Title”:ehealth OR “Document Title”:e-health OR “Document Title”:telemedicine OR “Document Title”:tele-medicine OR “Document Title”:telehealth OR “Document Title”:tele-health) refined by:Year:2019-2024))  AND ((“Document Title”:health OR “Document Title”:medical OR “Document Title”:clinic OR “Document Title”:clinical) refined by:Year:2019-2024 ))  AND ((“Document Title” OR Abstract:intervention OR program OR therapy OR prevention OR treatment OR app OR application))  AND ((“Document Title” OR Abstract: assessment OR assessing OR evaluation OR evaluating OR criteria OR rating OR score OR scoring)))) |
| --- | --- |
| 2 | (“Document Title”:digital OR “Document Title”:mobile OR “Document Title”:app OR “Document Title”:mobile application OR “Document Title”:mobile app OR “Document Title”:web “Document Title”:internet OR “Document Title”:smartphone OR “Document Title”:phone OR “Document Title”:mobile-phone OR “Document Title”:electronic OR “Document Title”:mhealth OR “Document Title”:m-health OR “Document Title”:ehealth OR “Document Title”:e-health OR “Document Title”:telemedicine  refined by: Year: 2019-2024 |
| 3 | (((“Document Title” OR Abstract: intervention OR program OR therapy OR prevention OR treatment OR app OR application))) |
| 4 | (((“Document Title” OR Abstract: assessment OR assessing OR evaluation OR evaluating OR criteria OR rating OR score OR scoring))) |
| 5 | #1 AND #2 AND #3 AND #4 |

**Appendix 1c: Searches to refresh the review conducted by Moshi et al.[3]**

**Medline:**

| **#** | **Query** |
| --- | --- |
| 1 | exp Mobile Applications/ |
| 2 | mobile app*.tw. |
| 3 | portable electronic app*.tw. |
| 4 | mhealth app*.tw. |
| 5 | m-health app*.tw. |
| 6 | portable software app*.tw. |
| 7 | mobile medical app*.tw. |
| 8 | mobile health app*.tw. |
| 9 | exp Telemedicine/ |
| 10 | telemedicine app*.tw. |
| 11 | telehealth app*.tw. |
| 12 | tele-medicine app*.tw. |
| 13 | tele-health app*.tw. |
| 14 | electronic health app*.tw. |
| 15 | e-health app*.tw. |
| 16 | ehealth app*.tw. |
| 17 | 1 or 2 or 3 or 4 or 5 or 6 or 7 or 8 or 9 or 10 or 11 or 12 or 13 or 14 or 15 or 16 |
| 18 | exp Cell Phone/ |
| 19 | exp Smartphone/ |
| 20 | cell phone*.tw. |
| 21 | cellphone*.tw. |
| 22 | cell-phone*.tw. |
| 23 | cellular phone*.tw. |
| 24 | cellular telephone*.tw. |
| 25 | mobile phone*.tw. |
| 26 | mobile.tw. |
| 27 | mobile device*.tw. |
| 28 | mobile platform*.tw. |
| 29 | smartphone*.tw. |
| 30 | smart phone*.tw. |
| 31 | android*.tw. |
| 32 | ipad*.tw. |
| 33 | iphone*.tw. |
| 34 | apple watch*.tw. |
| 35 | smart watch*.tw. |
| 36 | tablet*.tw. |
| 37 | iOS.tw. |
| 38 | Blackberr*.tw. |
| 39 | windows.tw. |
| 40 | Microsoft.tw. |
| 41 | google.tw. |
| 42 | apple.tw. |
| 43 | HTC.tw. |
| 44 | 18 or 19 or 20 or 21 or 22 or 23 or 24 or 25 or 26 or 27 or 28 or 29 or 30 or 31 or 32 or 33 or 34 or 35 or 36 or 37 or 38 or 39 or 40 or 41 or 42 or 43 |
| 45 | 17 and 44 |
| 46 | evaluat*.tw. |
| 47 | criteri*.tw. |
| 48 | apprais*.tw. |
| 49 | 46 or 47 or 48 |
| 50 | 45 and 49 |
| 51 | limit 50 to dt=20161101-20240128 |

**Psychinfo:**

| **#** | **Query** |
| --- | --- |
| 1 | exp mobile applications/ |
| 2 | mobile app*.tw. |
| 3 | portable electronic app*.tw. |
| 4 | mhealth app*.tw. |
| 5 | m-health app*.tw. |
| 6 | portable software app*.tw. |
| 7 | mobile medical app*.tw. |
| 8 | mobile health app*.tw. |
| 9 | exp Telemedicine/ |
| 10 | telemedicine app*.tw. |
| 11 | telehealth app*.tw. |
| 12 | tele-medicine app*.tw. |
| 13 | tele-health app*.tw. |
| 14 | electronic health app*.tw. |
| 15 | e-health app*.tw. |
| 16 | ehealth app*.tw. |
| 17 | 1 or 2 or 3 or 4 or 5 or 6 or 7 or 8 or 9 or 10 or 11 or 12 or 13 or 14 or 15 or 16 |
| 18 | exp Cell Phone/ |
| 19 | exp smartphones/ |
| 20 | cell phone*.tw. |
| 21 | cellphone*.tw. |
| 22 | cell-phone*.tw. |
| 23 | cellular phone*.tw. |
| 24 | cellular telephone*.tw. |
| 25 | mobile phone*.tw. |
| 26 | mobile.tw. |
| 27 | mobile device*.tw. |
| 28 | mobile platform*.tw. |
| 29 | smartphone*.tw. |
| 30 | smart phone*.tw. |
| 31 | android*.tw. |
| 32 | ipad*.tw. |
| 33 | iphone*.tw. |
| 34 | apple watch*.tw. |
| 35 | smart watch*.tw. |
| 36 | tablet*.tw. |
| 37 | iOS.tw. |
| 38 | Blackberr*.tw. |
| 39 | windows.tw. |
| 40 | Microsoft.tw. |
| 41 | google.tw. |
| 42 | apple.tw. |
| 43 | HTC.tw. |
| 44 | 18 or 19 or 20 or 21 or 22 or 23 or 24 or 25 or 26 or 27 or 28 or 29 or 30 or 31 or 32 or 33 or 34 or 35 or 36 or 37 or 38 or 39 or 40 or 41 or 42 or 43 |
| 45 | 17 and 44 |
| 46 | evaluat*.tw. |
| 47 | criteri*.tw. |
| 48 | apprais*.tw. |
| 49 | exp evaluation criteria/ |
| 50 | exp Rating/ |
| 51 | 46 or 47 or 48 or 49 or 50 |
| 52 | 45 and 51 |
| 53 | limit 52 to up=20161101-20240128 |

**CINAHL:**

| # | Query |
| --- | --- |
| S1 | MH mobile applications |
| S2 | TI mobile app* OR AB mobile app* |
| S3 | TI portable electronic app* OR AB portable electronic app* |
| S4 | TI mhealth app* OR AB mhealth app OR TI m-health app OR AB m-health app OR TI mhealth app OR AB mhealth app* |
| S5 | TI portable software app OR AB portable software app* |
| S6 | TI mobile medical app* OR AB mobile medical app* |
| S7 | MH telemedicine OR MH tele-medicine |
| S8 | AB telemedicine app* OR TI telemedicine app* OR TI tele-medicine app OR AB tele-medicine app* |
| S9 | MH telehealth OR MH tele-health |
| S10 | TI telehealth app* OR AB telehealth app* OR TI tele-health app* OR AB tele-health app* |
| S11 | TI electronic health app* OR AB electronic health app* |
| S12 | TI ehealth app OR AB ehealth app OR TI e-Health app OR AB e-Health app* |
| S13 | S1 OR S2 OR S3 OR S4 OR S5 OR S6 OR S7 OR S8 OR S9 OR S10 OR S11 OR S12 |
| S14 | MH cell phones OR MH smartphone OR MH smartphones OR MH cellphones |
| S15 | TI cell phone* OR AB cell phone OR TI cellphone* OR AB cellphone* |
| S16 | TI cellular phone* OR AB cellular phone |
| S17 | TI cellular telephone* OR AB cellular telephone* |
| S18 | TI mobile phone* OR AB mobile phone* |
| S19 | AB mobile OR TI mobile |
| S20 | Tl mobile device* OR AB mobile device* |
| S21 | AB mobile platform* OR TI mobile platform* |
| S22 | TI smartphone* OR AB smartphone* |
| S23 | TI smart phone* OR AB smart phone* |
| S24 | TI android OR AB android* |
| S25 | TI ipad* OR AB ipad* |
| S26 | TI iphone OR AB iphone* |
| S27 | Tl apple watch* OR AB apple watch* |
| S28 | Tl smart watch* OR AB smart watch* |
| S29 | TI tablet OR AB tablet* |
| S30 | TI IOS OR AB IOS |
| S31 | TI blackberr* OR AB blackberr* |
| S32 | TI windows OR AB windows |
| S33 | TI microsoft OR AB microsoft |
| S34 | TI google OR AB google |
| S35 | TI apple OR AB apple |
| S36 | TI htc OR AB htc |
| S37 | S14 OR S15 OR S16 OR S17 OR S18 OR S19 OR S20 OR S21 OR S22 OR S23 OR S24 OR S25 OR S26 OR S27 OR S28 OR S29 OR S30 OR S31 OR S32 OR S33 OR S34 OR S35 OR S36 |
| S38 | MH criteria for evaluation |
| S39 | TI apprais* OR AB apprais* |
| S40 | TI criteri OR AB criteri* |
| S41 | MH evaluation or assessment |
| S42 | MH appraisal |
| S43 | Tl evaluat OR AB evaluat* |
| S44 | S38 OR S39 OR S40 OR S41 OR S42 OR S43 |
| S45 | S13 AND S37 AND S44 |
| S46 | S13 AND S37 AND S44 |

**Cochrane library:**

| **No.** | **Search** |
| --- | --- |
| **#1** | MeSH descriptor: [Mobile Applications] explode all trees |
| **#2** | MeSH descriptor: [Telemedicine] explode all trees |
| **#3** | (mobile app*):ab OR (mobile app*):ti OR (m*health app*):ab OR (m*health app*):ti |
| **#4** | (portable electronic app*):ti OR (portable electronic app*):ab OR (portable software app*):ti OR (portable software app*):ab |
| **#5** | (mobile medical app*):ab OR (mobile medical app*):ti OR (mobile health app*):ti OR (mobile health app*):ab |
| **#6** | (tele*medicine app*):ab OR (tele*medicine app*):ti OR (e*health app*):ab OR (e*health app*):ti |
| **#7** | (tele*health app*):ab OR (tele*health app*):ti |
| **#8** | MeSH descriptor: [Evaluation Studies as Topic] explode all trees |
| **#9** | (evaluat*):ti OR (evaluat*):ab OR (criteri*):ti OR (criteri*):ab |
| **#10** | (apprais*):ab OR (apprais*):ti |
| **#11** | #1 OR #2 OR #3 OR #4 OR #5 OR #6 OR #7 |
| **#12** | #8 OR #9 OR #10 |
| **#13** | #11 AND #12 with Cochrane Library publication date Between Nov 2016 and Jan 2024 |
| **#14** | #13 in Cochrane Reviews, Cochrane Protocols |

**Compendex and Business source complete:**

(((((((("mobile medical app*" OR "mobile health app*" OR "mobile health application" OR "tele*medicine app*" OR "tele*medicine application" OR "m*Health app*" OR "m*health application"  OR "e*Health app*" OR "e*Health application" OR "tele*health app*" "tele*health application")) WN ALL) AND (((evaluat* OR criteri* OR apprais*)) WN ALL)) AND (((tool* OR framework*)) WN ALL)) AND ((medic*) WN ALL))) AND ((2024 OR 2023 OR 2022 OR 2021 OR 2020 OR 2019 OR 2018 OR 2017 OR 2016) WN YR))

**Appendix 1d: Searches to refresh the review conducted by Nouri et al [4]**

**Medline:**

| **#** | **Query** |
| --- | --- |
| 1 | exp Mobile Applications/ |
| 2 | "mhealth application*1 ".tw. |
| 3 | "mhealth app*1 ".tw. |
| 4 | "m-health app*1 ".tw. |
| 5 | "m-health application*1 ".tw. |
| 6 | (mobile adj2 app*1).tw. |
| 7 | (mobile adj2 application*1).tw. |
| 8 | ("smart-phone" adj2 app*1).tw. |
| 9 | ("smart-phone" adj2 application*1).tw. |
| 10 | (smartphone adj2 app*1).tw. |
| 11 | (smartphone adj2 application*1).tw. |
| 12 | 1 or 2 or 3 or 4 or 5 or 6 or 7 or 8 or 9 or 10 or 11 |
| 13 | ("assess*" or "evaluat*" or "measur*" or score? or scoring or criteri* or scale? or scaling).ti. |
| 14 | 12 and 13 |
| 15 | limit 14 to dt=20161223-20240128 |

**Embase:**

| **#** | **Query** |
| --- | --- |
| 1 | exp mobile application/ |
| 2 | ("m health app*" or "m-health app*").hw. |
| 3 | exp mobile health application/ |
| 4 | (mobile adj2 app*).hw. |
| 5 | ("smartphone adj2 app*" or "smart phone adj2 app*").hw. |
| 6 | 1 or 2 or 3 or 4 or 5 |
| 7 | (assess* or evaluat* or measur* or score*OR scoring or criteri* or scale*OR scaling).ti. |
| 8 | 6 and 7 |
| 9 | limit 8 to dc=20161223-20240128 |

**Scopus:**

( TITLE-ABS-KEY ( ( "m-health app*" OR "mhealth app*" OR "mobile health app*" OR "smartphone health app*" OR "smart phone health app*" OR "smartphone medical app*" OR "smart-phone medical app*" OR "mobile-phone health app*" OR "mobile-phone medical app*" OR "health mobile phone app*" OR "health mobile app*" OR "health medical app*" OR "Mobile healthcare app*" OR "Mobile medical app*" OR "medical mobile app*" ) )

AND TITLE ( ( "evaluat*" OR "assess*" OR "measur*" OR "score" OR "score?" OR "scoring" OR "criteri*" OR "scale" OR "scale?" OR "scaling" ) ) )

AND PUBYEAR > 2015

AND PUBYEAR < 2025

**Web of Science:**

( TITLE-ABS-KEY ( ( "m-health app*" OR "mhealth app*" OR "mobile health app*" OR "smartphone health app*" OR "smart phone health app*" OR "smartphone medical app*" OR "smart-phone medical app*" OR "mobile-phone health app*" OR "mobile-phone medical app*" OR "health mobile phone app*" OR "health mobile app*" OR "health medical app*" OR "Mobile healthcare app*" OR "Mobile medical app*" OR "medical mobile app*" ) )

AND TITLE ( ( "evaluat*" OR "assess*" OR "measur*" OR "score" OR "score?" OR "scoring" OR "criteri*" OR "scale" OR "scale?" OR "scaling" ) ) )

AND PUBYEAR > 2015

AND PUBYEAR < 2025

**References:**

1. Lagan S, Sandler L, Torous J. Evaluating evaluation frameworks: a scoping review of frameworks for assessing health apps. BMJ Open. 2021 Mar 19;11(3):e047001. PMID: 33741674. doi: 10.1136/bmjopen-2020-047001.

2. Kowatsch T, Otto L, Harperink S, Cotti A, Schlieter H. A design and evaluation framework for digital health interventions. 2019;61(5-6):253-63. doi: doi:10.1515/itit-2019-0019.

3. Moshi MR, Tooher R, Merlin T. Suitability of current evaluation frameworks for use in the health technology assessment of mobile medical applications: a systematic review. International journal of technology assessment in health care. 2018;34(5):464-75. doi: https://dx.doi.org/10.1017/S026646231800051X.

4. Nouri R, Kalhori NRS, Ghazisaeedi M, Marchand G, Yasini M. Criteria for assessing the quality of mHealth apps: a systematic review. Journal of the American Medical Informatics Association. 2018;25(8):1089-98. doi: 10.1093/jamia/ocy050.
