# Supplementary material for: Suitable Evaluation Frameworks for Disease-Agnostic Platforms for Remote Patient Monitoring: Scoping Review
Source: J Med Internet Res. 2025 Jun 16;27:e68910. doi: 10.2196/68910 (PMC12209728; doi:10.2196/68910)
Supplement: Multimedia Appendix 3 [file jmir_v27i1e68910_app3.docx]

Appendix 3 – Summarised extraction of included studies

| **Author, citation and title of study** | | | **Cited framework(s)** | **Notes** |
| --- | --- | --- | --- | --- |
| Schliemann 2022 |  | Implementation of a colorectal cancer screening intervention in Malaysia (CRC-SIM) in the context of a pandemic: Study protocol | Reach Effectiveness Adoption Implementation and Maintenance (RE-AIM) |  |
| Rasooly 2022 |  | Quality and Performance Measurement in Primary Diabetes Care: A Qualitative Study in Urban China | Consolidated Framework for Implementation Research (CFIR) |  |
| MacMillanUribe 2022 |  | O25 Evaluation of Commercially Available Infant Feeding Mobile Applications Using the App Quality Evaluation Tool | App Quality Evaluation Tool (AQEL) |  |
| DiSebastiano 2022 |  | An Evaluation of a Commercialized mHealth Intervention to Promote Physical Activity in the Workplace | Reach Effectiveness Adoption Implementation and Maintenance (RE-AIM) framework |  |
| Bu 2022 |  | Optimising implementation of telehealth in oncology: A systematic review examining barriers and enablers using the RE-AIM planning and evaluation framework | Reach Effectiveness Adoption Implementation and Maintenance (RE-AIM) |  |
| Bernard 2022 |  | Strategies for Implementing Occupational eMental Health Interventions: Scoping Review | Consolidated Framework for Implementation Research (CFIR)  Reach Effectiveness Adoption Implementation and Maintenance (RE-AIM) |  |
| Ko 2021 |  | The Development of a Mobile Application for Older Adults for Rehabilitation Instructions After Hip Fracture Surgery | ADDIE (analysis, design, development, implementation, and evaluation) |  |
| Garvin 2021 |  | Use of Video Telehealth Tablets to Increase Access for Veterans Experiencing Homelessness | Reach Effectiveness Adoption Implementation and Maintenance (RE-AIM) |  |
| Mosch 2020 |  | Evaluating the implementation of a remote patient monitoring platform in the ICU: A qualitative study | Consolidated Framework for Implementation Research (CFIR) |  |
| Johnson 2020 |  | Engaging Parents in Education for Discharge (ePED): Evaluating the Reach, Adoption & Implementation of an Innovative Discharge Teaching Method | Reach Effectiveness Adoption Implementation and Maintenance (RE-AIM) |  |
| Furlonger 2020 |  | Ways in which school psychologists can identify suitable apps for supporting the self-management of asthma by students | Behaviour Change Technique Taxonomy Version One (BCTTv1 |  |
| Denecke 2020 |  | How to Evaluate Health Applications with Conversational User Interface?...30th Medical Informatics Europe Conference | Unnamed framework for chatbot development |  |
| Avdagovska 2020 |  | Capturing the impact of patient portals based on the quadruple aim and benefits evaluation frameworks: Scoping review | Benefits evaluation framework | Framework designed in 2006 and updated by Lau et al in 2010. |
| Steigerwalt 2019 |  | A novel mobile app's reliability, end user satisfaction, and changes in dash diet eating patterns over 8 weeks | App Quality Evaluation Tool (AQEL) |  |
| Siaw-Teng 2019 |  | Use of mHealth for promoting healthy ageing and supporting delivery of age-friendly care services: a systematic review | Reach Effectiveness Adoption Implementation and Maintenance (RE-AIM) |  |
| Rogers 2019 |  | Barriers and Facilitators to the Implementation of a Mobile Insulin Titration Intervention for Patients With Uncontrolled Diabetes: A Qualitative Analysis | Consolidated Framework for Implementation Research (CFIR) |  |
| Koot 2019 |  | A Mobile Lifestyle Management Program (GlycoLeap) for People With Type 2 Diabetes: Single-Arm Feasibility Study | Reach Effectiveness Adoption Implementation and Maintenance (RE-AIM) |  |
| Ide 2019 |  | People welcomed this innovation with two hands: A qualitative report of an mhealth intervention for community case management in Malawi | Consolidated Framework for Implementation Research (CFIR) |  |
| Hay-Smith 2019 |  | Apps-olutely fabulous?-the quality of pfmt smartphone app content and design rated using the mobile app rating scale, behaviour change taxonomy, and guidance for exercise prescription | BCT taxonomy |  |
| Felix 2019 |  | Development of a complex intervention to improve adherence to antidiabetic medication in older people using an anthropomorphic virtual assistant software | BCTTv1 |  |
| Blok 2019 |  | Nurse-Driven mHealth Implementation Using the Technology Inpatient Program for Smokers (TIPS): Mixed Methods Study | Reach Effectiveness Adoption Implementation and Maintenance (RE-AIM) |  |
| Arrossi 2019 |  | Mixed-methods approach to evaluate an mHealth intervention to increase adherence to triage of human papillomavirus-positive women who have performed self-collection (the ATICA study): study protocol for a hybrid type I cluster randomized effectiveness-implementation trial | Consolidated Framework for Implementation Research (CFIR)  Reach, Effectiveness, Adoption, Implementation, and Maintenance (RE-AIM) | Contains two frameworks.  Authors argue that RE-AIM is particularly appropriate for assessing public health impact of interventions. And that CFIR useful for systematically assessment contexual factors that influence adoption and implementation. |
| Stockner 2018 |  | Using the RE-AIM Framework in Formative Evaluation/Planning of a Mobile Prehospital Telestroke Intervention in an Urban Setting: Pilot Data for the Prehospital Rapid Evaluation via Ambulance Lead Emergency Remote Telemedicine (PRE-ALERT) Study | Reach Effectiveness Adoption Implementation and Maintenance (RE-AIM) |  |
| Lakerveld 2018 |  | Improving cardiometabolic health through nudging dietary behaviours and physical activity in low SES adults: design of the Supreme Nudge project | Reach Effectiveness Adoption Implementation and Maintenance (RE-AIM) |  |
| Xie 2017 |  | Chinese Cardiovascular Disease Mobile Apps' Information Types, Information Quality, and Interactive Functions for Self-Management: Systematic Review | Self-Management Framework for Evaluating Interactive App Functions (SFEIAF) |  |
| Tinschert 2017 |  | The Potential of Mobile Apps for Improving Asthma Self-Management: A Review of Publicly Available and Well-Adopted Asthma Apps | BCTTv1 |  |
| Sapru 2017 |  | Applying RE-AIM to evaluate two community-based programs designed to improve access to eye care for those at high-risk for glaucoma | Reach Effectiveness Adoption Implementation and Maintenance (RE-AIM) |  |
| Mehmood 2017 |  | Development of an mHealth trauma registry in the Middle East using an implementation science framework | Modified ICT implementation framework |  |
| Vallespin 2016 |  | Ensuring evidence-based safe and effective mHealth applications | Framework for continuous systemic evaluation for Health IT systems  HOT-fit |  |
| Garg 2016 |  | Qualitative analysis of programmatic initiatives to text patients with mobile devices in resource-limited health systems | Consolidated Framework for Implementation Research (CFIR) |  |
| Panda 2023 |  | Perceptions of Mobile Health Technology in Elective Surgery: A Qualitative Study of North American Surgeons | Consolidated Framework for Implementation Research (CFIR) |  |
| MacMillanUribe 2023 |  | Appropriateness and Relevance of Infant Feeding Mobile Applications for Lesbian, Gay, Bisexual, Transgender, or Queer Mothers...Society for Nutrition Education and Behavior (SNEB), 55th Annual Conference, July 20-23, 2023, Washington, DC | App Quality Evaluation Tool (AQEL) |  |
| Khalid 2023 |  | Implementation Science Perspectives on Implementing Telemedicine Interventions for Hypertension or Diabetes Management: Scoping Review | Consolidated Framework for Implementation Research (CFIR) |  |
| Cox 2023 |  | Tele rehabilitation in the 'Real World': Implementation of Remotely Delivered Pulmonary Rehabilitation | Reach Effectiveness Adoption Implementation and Maintenance (RE-AIM) |  |
| Cox 2023 |  | Telerehabilitation in the 'real-world': Implementation of remotely delivered pulmonary rehabilitation | Reach, Effectiveness, Adoption, Implementa-  tion, Maintenance framework (RE-AIM) |  |
| Brill 2023 |  | Implementing the Better Starts For All Pilot Mobile and Telehealth Intervention in Ohio Appalachia: Improving Access to Maternal Healthcare | Reach Effectiveness Adoption Implementation and Maintenance (RE-AIM) |  |
| Aydin 2023 |  | Mobile care app development process: using the ADDIE model to manage symptoms after breast cancer surgery (step 1) | ADDIE (Analysis, Design, Development, Implementation, Evaluation) |  |
| Youn 2023 |  | Leveraging Implementation Science to Integrate Digital Mental Health Interventions as part of Routine Care in a Practice Research Network | Reach Effectiveness Adoption Implementation and Maintenance (RE-AIM) |  |
| Straw 2023 |  | Implementation and scaling-up of an effective mHealth intervention to increase adherence to triage of HPV-positive women (ATICA study): perceptions of health decision-makers and health-care providers | Reach Effectiveness Adoption Implementation and Maintenance (RE-AIM)  Consolidated Framework for Implementation Research (CFIR) |  |
| Sharma 2023 |  | A systematic review assessing the state of analytical validation for connected, mobile, sensor-based digital health technologies | V3 Framework | Supported by EVIDENCE checklist to define appropriate reporting criteria for studies evaluating these products. |
| Lundstrom 2023 |  | Effectiveness of Internet-based cognitive-behavioural therapy for obsessive-compulsive disorder (OCD-NET) and body dysmorphic disorder (BDD-NET) in the Swedish public health system using the RE-AIM implementation framework | Reach Effectiveness Adoption Implementation and Maintenance (RE-AIM) |  |
| Li 2023 |  | Barriers and facilitators of implementing electronic monitors to improve adherence and health outcomes in tuberculosis patients: protocol for a systematic review based on the Consolidated Framework for Implementation Research | Consolidated Framework for Implementation Research (CFIR) |  |
| LeLaurin 2023 |  | Pediatric primary care provider and staff perspectives on the implementation of electronic health record-based social needs interventions: A mixed-methods study | Consolidated Framework for Implementation Research (CFIR) |  |
| Knapp 2023 |  | "The library is so much more than books": considerations for the design and implementation of teen digital mental health services in public libraries | Consolidated Framework for Implementation Research (CFIR) |  |
| Holloway 2023 |  | Low-Intensity mental health Support via a Telehealth Enabled Network for adults with diabetes (LISTEN): protocol for a hybrid type 1 effectiveness implementation trial | Reach Effectiveness Adoption Implementation and Maintenance (RE-AIM) |  |
| Hailemariam 2023 |  | Individual and contextual level enablers and barriers determining electronic community health information system implementation in northwest Ethiopia | Consolidated Framework for Implementation Research (CFIR) |  |
| Gisondi 2023 |  | Teaching LGBTQ+ Health, a Web-Based Faculty Development Course: Program Evaluation Study Using the RE-AIM Framework | Reach Effectiveness Adoption Implementation and Maintenance (RE-AIM) |  |
| Gamble 2023 |  | Telehealth Diabetes Prevention Program for Adults With Prediabetes in an Academic Medical Center Setting: Protocol for a Hybrid Type III Trial | Reach Effectiveness Adoption Implementation and Maintenance (RE-AIM) |  |
| Freund 2023 |  | Using the Consolidated Framework for Implementation Research to evaluate a nationwide depression prevention project (ImplementIT) from the perspective of health care workers and implementers: Results on the implementation of digital interventions for farmers | Consolidated Framework for Implementation Research (CFIR) |  |
| Bagsic 2023 |  | Process evaluation of Dulce Digital-Me: an adaptive mobile health (mHealth) intervention for underserved Hispanics with diabetes | Reach Effectiveness Adoption Implementation and Maintenance (RE-AIM) |  |
| Yudkin 2022 |  | Needs Assessment and Best Practices for Digital Trainings for Health Professionals in Ethiopia Using the RE-AIM Framework: COVID-19, Case Study | Reach Effectiveness Adoption Implementation and Maintenance (RE-AIM) |  |
| Stump 2022 |  | Development of an Implementation Facilitation Strategy to Link Mental Health Screening and eHealth Intervention for Clients in Ryan White-Funded Clinics in Chicago | Consolidated Framework for Implementation Research (CFIR) |  |
| Simon 2022 |  | DIGITAL HEALTH INTERVENTIONS FOR PAIN IN PEDIATRIC ONCOLOGY: STATE OF THE FIELD | Consolidated Framework for Implementation Research (CFIR)  Reach Effectiveness Adoption Implementation and Maintenance (RE-AIM) | Also includes knowledge to action framework (not an evaluation framework) |
| Mosch 2022 |  | Creation of an Evidence-Based Implementation Framework for Digital Health Technology in the Intensive Care Unit: Qualitative Study | Consolidated Framework for Implementation Research (CFIR) |  |
| Mantri 2022 |  | Assessment of e-aushadhi program (drug inventory e-health initiative in Rajasthan) using benefit evaluation framework | Benefits Evaluation (BE) framework | Framework is based on work by DeLone and McLean (1992, 2003). Original citation not included in paper |
| Holt 2022 |  | Real-world implementation evaluation of an electronic health record-integrated consumer informatics tool that collects patient-generated contextual data | Reach Effectiveness Adoption Implementation and Maintenance (RE-AIM) |  |
| Hodges 2022 |  | Evaluation of the Implementation and Effectiveness of a Mobile Health Intervention to Improve Outcomes for People With HIV in the Washington, DC Cohort: Study Protocol for a Cluster Randomized Controlled Trial | Reach Effectiveness Adoption Implementation and Maintenance (RE-AIM)  Consolidated Framework for Implementation Research (CFIR) |  |
| Dwyer 2022 |  | Navigating Disrupted Puberty: Development and Evaluation of a Mobile-Health Transition Passport for Klinefelter Syndrome | Patient Education Materials Assessment Tool (PEMAT) |  |
| Castor 2022 |  | Assessment of the implementation context in preparation for a clinical study of machine-learning algorithms to automate the classification of digital cervical images for cervical cancer screening in resource-constrained settings | Consolidated Framework for Implementation Research (CFIR) |  |
| Burton 2022 |  | A Comparative Utility Score for Digital Health Tools | Utility Factor Score |  |
| Yoshida 2020 |  | Using the RE-AIM framework to evaluate internal and external validity of mobile phone-based interventions in diabetes self-management education and support | Reach Effectiveness Adoption Implementation and Maintenance (RE-AIM) |  |
| vanReijen 2018 |  | Users' Perspectives, Opportunities, and Barriers of the Strengthen Your Ankle App for Evidence-Based Ankle Sprain Prevention: Mixed-Methods Process Evaluation for a Randomized Controlled Trial | Reach Effectiveness Adoption Implementation and Maintenance (RE-AIM) |  |
| Chapel 2022 |  | Standardization of the assessment process within telerehabilitation in chronic diseases: a scoping meta-review | Health technology assessment framework |  |
| Yu 2019 |  | Process Evaluation of the Diabetes Canada Guidelines Dissemination Strategy Using the Reach Effectiveness Adoption Implementation Maintenance (RE-AIM) Framework | Reach Effectiveness Adoption Implementation and Maintenance (RE-AIM) |  |
| MartinPayo 2019 |  | Prescribing fitness apps for people with cancer: a preliminary assessment of content and quality of commercially available apps | BCTTv1 |  |
| Bardosh 2017 |  | Operationalizing mHealth to improve patient care: A qualitative implementation science evaluation of the WelTel texting intervention in Canada and Kenya | Consolidated Framework for Implementation Research (CFIR) |  |
| DiFilippo 2017 |  | A New Tool for Nutrition App Quality Evaluation (AQEL): Development, Validation, and Reliability Testing | Nutrition App Quality Evaluation (AQEL) |  |
| DiFilippo 2018 |  | Mobile Apps for the Dietary Approaches to Stop Hypertension (DASH): App Quality Evaluation | App Quality Evaluation (AQEL) |  |
| Lehmann 2021 |  | MHealthAtlas - An approach for the multidisciplinary evaluation of mHealth applications | mhealth Atlas |  |
| Fiore 2017 |  | How to Evaluate Mobile Health Applications: A Scoping Review | AHRQ criteria  CRAAP-O  NPMEDAPP  Mobile medical application evaluation rubric | Tool to evaluate health information also available (RADAR) as well as tools to assess if an app is a medical device (canadian MDR guidance)  Some other frameworks already identified e.g. imedicalapps, Medical app journal, MobihealthNews (Aungst et al) |
| Sadegh 2018 |  | A framework for m-health service development and success evaluation | Unnamed framework |  |
| Capraş 2016 |  | An evaluation of free medical applications for android smartphones | Bespoke evaluation framework |  |
| McKay 2018 |  | Evaluating mobile phone applications for health behaviour change: A systematic review | Coventry, Aberdeen and London-Revised taxonomy (CALO-RE)  Abraham and Michie taxonomy of health behaviour change  BCTTv1 |  |
| Turnbull 2023 |  | Development of OSOMO Prompt Mobile Application on Elderly Population for Village Health Volunteers Using the Analysis, Design, Development, Implementation, and Evaluation (ADDIE) Model | Analysis, Design, Development,  Implementation, and Evaluation (ADDIE)  Model |  |
| Saeidnia 2022 |  | Development of a Mobile App for Self-Care Against COVID-19 Using the Analysis, Design, Development, Implementation, and Evaluation (ADDIE) Model: Methodological Study | Analysis, Design, Development, Implementation, and  Evaluation (ADDIE) Model |  |
| Son 2023 |  | Development and evaluation of a tailored mHealth parenting program for multicultural families: a three-arm cluster randomized controlled trial | Analysis, Design, Development, Implementation, and Evaluation (ADDIE) model |  |
| White 2016 |  | Designing evaluation plans for health promotion mHealth interventions: a case study of the Milk Man mobile app | Trial of intervention principles framework |  |
| Torquati 2018 |  | Changing Diet and Physical Activity in Nurses: A Pilot Study and Process Evaluation Highlighting Challenges in Workplace Health Promotion | Reach Effectiveness Adoption Implementation and Maintenance (RE-AIM) |  |
| Grau 2016 |  | Assessment method for mobile health applications in Spanish: The iSYScore index | iSYScore Index |  |
| Wang 2020 |  | An analysis and evaluation of quality and behavioral change techniques among physical activity apps in China | BCTTv1 |  |
| Woulfe 2021 |  | Identification and Evaluation of Methodologies to Assess the Quality of Mobile Health Apps in High-, Low-, and Middle-Income Countries: Rapid Review | App Quality Evaluation Tool (AQEL)  Six Aims for Quality Improvement |  |
| Wang 2021 |  | Where Should Mobile Health Application Providers Focus Their Goals? | mobile health application evaluation system (MHAES) |  |
| Spencer-Brown 2021 |  | Evaluation of an Electronic Health Record Referral Process to Enhance Participation in Evidence-Based Arthritis Interventions | Consolidated Framework for Implementation Research (CFIR) |  |
| Reisinger-Kindle 2021 |  | Evaluation of rapid telehealth implementation for prenatal and postpartum care visits during the COVID-19 pandemic in an academic clinic in Springfield, Massachusetts, United States of America | Reach Effectiveness Adoption Implementation and Maintenance (RE-AIM) |  |
| Pach 2021 |  | Five Lessons Learned From Randomized Controlled Trials on Mobile Health Interventions: Consensus Procedure on Practical Recommendations for Sustainable Research | Reach Effectiveness Adoption Implementation and Maintenance (RE-AIM) | Also cites: Dansky KH, Thompson D, Sanner T. A framework for evaluating eHealth research. Eval Program Plann. 2006 Nov;29(4):397–404. doi: 10.1016/j.evalprogplan.2006.08.009.  This is a very high level framework on how to address ehealth research issues more generally. |
| Joshi 2021 |  | Approaches to Assess E-Health Programs: A Scoping Review | Constructive eHealth evaluation method (CeHEM)  Digital health benefit evaluation (BE) framework  Lifecycle evaluation framework  International Network of Agencies for Health Technology Assessment (INAHTA) checklist  WHO guideline on monitoring and evaluating DHIs  Clinical adoption framework (CAF)  Clinical adoption meta-model (CAMM)  Pragmatic health information technology evaluation framework | "Report of the Expert Panel on Effective Ways of Investing in Health, 2019, mentioned that most of the developed evaluation frameworks for evaluating digital health services were guided by health technology assessment (HTA) methodology"  Murray et al do not present a framework, but high level questions.  Nabukenya and Justus Ashaba do not present a framework but discuss methodlogies which DHT evaluation can consider  Lau and Kuziemsky discuss several models, some of which not suitable (e.g. economic evaluation) |
| Halminen 2021 |  | Demonstrating the value of digital health: Guidance on contextual evidence gathering for companies in different stages of maturity | WHO guide: Monitoring and Evaluating Digital Health Interventions: A practical guide to conducting research and assessment. |  |
| Gong 2021 |  | The Implementation of a Primary Care-Based Integrated Mobile Health Intervention for Stroke Management in Rural China: Mixed-Methods Process Evaluation | Reach Effectiveness Adoption Implementation and Maintenance (RE-AIM) |  |
| Daniels 2021 |  | 77 Using Mobile Integrated Health and Telehealth to Support Transition of Care Amond Heart Failure Patients: Mighty Heart Study Protocol | Reach Effectiveness  Adoption Implementation and Maintenance (RE-AIM) framework |  |
| Beauchemin 2021 |  | Incorporating systematic financial screening into the electronic health record | Consolidated Framework for Implementation Research (CFIR) |  |
| Vasudevan 2020 |  | Using digital health to facilitate compliance with standardized pediatric cancer treatment guidelines in Tanzania: Protocol for an early-stage effectiveness-implementation hybrid study | Consolidated Framework for Implementation Research (CFIR)  Reach Effectiveness Adoption Implementation and Maintenance (RE-AIM) |  |
| Steinman 2020 |  | Can mHealth and eHealth improve management of diabetes and hypertension in a hard-to-reach population? -lessons learned from a process evaluation of digital health to support a peer educator model in Cambodia using the RE-AIM framework br | Reach Effectiveness Adoption Implementation and Maintenance (RE-AIM) |  |
| Pithara 2020 |  | Implementing a Digital Tool to Support Shared Care Planning in Community-Based Mental Health Services: Qualitative Evaluation | Consolidated Framework for Implementation Research (CFIR) |  |
| Nolan 2020 |  | Design and impact evaluation of a digital reproductive health program in Rwanda using a cluster randomized design: study protocol | Proctor's implementation science framework |  |
| Minian 2020 |  | Impact of a Web-Based Clinical Decision Support System to Assist Practitioners in Addressing Physical Activity and/or Healthy Eating for Smoking Cessation Treatment: Protocol for a Hybrid Type I Randomized Controlled Trial | Reach Effectiveness Adoption Implementation and Maintenance (RE-AIM) |  |
| Meinert 2020 |  | Agile Requirements Engineering and Software Planning for a Digital Health Platform to Engage the Effects of Isolation Caused by Social Distancing: Case Study | Reach Effectiveness Adoption Implementation and Maintenance (RE-AIM) | Also includes Public Health England guidance on evaluating digital health products, which in turn recommends DTAC and NICE frameworks |
| Fuller 2020 |  | Interactive Digital Health Tools to Engage Patients and Caregivers in Discharge Preparation: Implementation Study | Reach Effectiveness Adoption Implementation and Maintenance (RE-AIM) |  |
| Freund 2020 |  | Implementing internet-and tele-based interventions to prevent mental health disorders in farmers, foresters and gardeners (ImplementIT): Study protocol for the multi-level evaluation of a nationwide project | Reach Effectiveness Adoption Implementation and Maintenance (RE-AIM) framework  Consolidated Framework for Implementation Research (CFIR) |  |
| Batsis 2020 |  | Barriers and facilitators in implementing a pilot, pragmatic, telemedicine-delivered healthy lifestyle program for obesity management in a rural, academic obesity clinic | Reach Effectiveness Adoption Implementation and Maintenance (RE-AIM)  Consolidated Framework for Implementation Research (CFIR) |  |
| Bashi 2020 |  | Digital health interventions for chronic diseases: a scoping review of evaluation frameworks | TECH model (TElehealth in CHronic Disease) | They identify other frameworks (conceptual, results, logical theory of change etc) but many did not report evaluation approach. |
| VanDerHout 2019 |  | E-health self-management applications targeting cancer survivors-who do we reach? | Reach Effectiveness Adoption Implementation and Maintenance (RE-AIM) |  |
| Rahal 2019 |  | Primary Care Physicians' Experience Using Advanced Electronic Medical Record Features to Support Chronic Disease Prevention and Management: Qualitative Study | Extended Clinical Adoption Framework |  |
| Girman 2019 |  | How do we assess when electronic health records or claims databases are fit for a specific research or regulatory purpose? | FDA framework for use of real-world evidence |  |
| Abdel-Wahab 2019 |  | A comprehensive scoping review to identify standards for the development of health information resources on the internet | Jones Instrument  Healthcare Website Assessment Instrument | Only included validated tools which covered multiple domains including evidence-based content, were condition agnostic and which were not focussed purely on the content. |
| Blackman 2013 |  | Assessing the internal and external validity of mobile health physical activity promotion interventions: a systematic literature review using the RE-AIM framework | Reach Effectiveness Adoption Implementation and Maintenance (RE-AIM) |  |
| Boatin 2021 |  | Wireless versus routine physiologic monitoring after cesarean delivery to reduce maternal morbidity and mortality in a resource-limited setting: protocol of type 2 hybrid effectiveness-implementation study | Reach Effectiveness Adoption Implementation and Maintenance (RE-AIM) |  |
| Savira 2023 |  | Virtual Care Initiatives for Older Adults in Australia: Scoping Review | World Health Organization (WHO) digital health evaluation framework |  |
| Fernando 2023 |  | Using Theories, Models, and Frameworks to Inform Implementation Cycles of Computerized Clinical Decision Support Systems in Tertiary Health Care Settings: Scoping Review | Reach Effectiveness Adoption Implementation and Maintenance (RE-AIM)  Consolidated Framework for Implementation Research (CFIR) | Jacobsohn et al draws on REAIM framework |
| Reid 2022 |  | Using the Consolidated Framework for Implementation Research to Inform the Design of the Mobile Inspección Visual con Ácido Acético System: Mixed Methods Case Study | Consolidated Framework for Implementation Research (CFIR) |  |
| Fan 2023 |  | Uptake of an App-Based Case Management Service for HIV-Positive Men Who Have Sex With Men in China: Process Evaluation Study | Linnan and Steckler Framework |  |
| Tiwari 2023 |  | A Type II hybrid effectiveness-implementation study of an integrated CHW intervention to address maternal healthcare in rural Nepal | Reach Effectiveness Adoption Implementation and Maintenance (RE-AIM) |  |
| Tighe 2020 |  | Toward a Digital Platform for the Self-Management of Noncommunicable Disease: Systematic Review of Platform-Like Interventions | WHO guidance on monitoring and evaluating digital health interventions |  |
| Du 2023 |  | "They Can't Believe They're a Tiger": Insights from pediatric speech-language pathologist mobile app users and app designers | Consolidated Framework for Implementation Research (CFIR) |  |
| Hussain 2021 |  | Technology assessment framework for precision health applications | IDEAL-D  Health technology assessment framework for Public Health interventions | Authors clarify that not all components of the HTA need to be applied to each technology (should be as appropriate for the target use case) |
| Brown 2023 |  | A Suicide Prevention Intervention for Emerging Adult Sexual and Gender Minority Groups: Protocol for a Pilot Hybrid Effectiveness Randomized Controlled Trial | Reach Effectiveness Adoption Implementation and Maintenance (RE-AIM) |  |
| Domin 2021 |  | Smartphone-Based Interventions for Physical Activity Promotion: Scoping Review of the Evidence Over the Last 10 Years | BCTTv1  CALO-RE taxonomy  BCT 26 item |  |
| Hensher 2021 |  | Scoping review: Development and assessment of evaluation frameworks of mobile health apps for recommendations to consumers | Self-developed checklist  CRAAP test (Currency, Relevance, Authority, Accuracy, and Purpose of information)  Unnamed survey  MedAd-AppQ ( Medication Adherence App Quality assessment tool) | Some frameworks not suitable, for example as only focussed on usability/feasibility (e.g. Spook et al), or bespoke criteria not designed to be presented as more widely adopted framework. |
| Schueller 2020 |  | Scaling evidence-based treatments through digital mental health | Consolidated Framework for Implementation Research (CFIR) |  |
| Suleman 2021 |  | Scale-up study protocol of the implementation of a mobile health sbirt approach for alcohol use reduction in mozambique | Reach Effectiveness Adoption Implementation and Maintenance (RE-AIM)  Consolidated Framework for Implementation Research (CFIR) |  |
| Lisowska 2023 |  | SATO (IDEAS expAnded wiTh BCIO): Workflow for designers of patient-centered mobile health behaviour change intervention applications | SATO (IDEAS expAnded wiTh BCIO) |  |
| Lessard 2022 |  | Remote Follow-up of Self-isolating Patients With COVID-19 Using a Patient Portal: Protocol for a Mixed Methods Pilot Study (Opal-COVID Study) | Consolidated Framework for Implementation Research (CFIR) |  |
| Tran 2022 |  | REDCap mobile data collection: Using implementation science to explore the potential and pitfalls of a digital health tool in routine voluntary medical male circumcision outreach settings in Zimbabwe | Reach Effectiveness Adoption Implementation and Maintenance (RE-AIM) |  |
| Berreta 2023 |  | A RE-AIM Analysis of a Mental Health App for Undergraduate and Medical Students during the COVID-19 Pandemic: A Retrospective Cross-Sectional Study | Reach Effectiveness Adoption Implementation and Maintenance (RE-AIM) |  |
| Ferrari 2022 |  | A Rapid-Learning Health System to Support Implementation of Early Intervention Services for Psychosis in Quebec, Canada: Protocol | Reach Effectiveness Adoption Implementation and Maintenance (RE-AIM) |  |
| Hayman 2021 |  | Quality, features, and presence of behavior change techniques in mobile apps designed to improve physical activity in pregnant women: Systematic search and content analysis | Taxonomy of BCTs |  |
| Laws 2021 |  | Protocol for an Effectiveness-Implementation Hybrid Trial to Evaluate Scale up of an Evidence-Based Intervention Addressing Lifestyle Behaviours From the Start of Life: INFANT | Reach Effectiveness Adoption Implementation and Maintenance (RE-AIM)  Outcomes for Implementation Research |  |
| Scherer 2022 |  | Proposal of a New Rating Concept for Digital Health Applications in Orthopedics and Traumatology | Bespoke evaluation tool for health-related apps |  |
| Wang 2021 |  | Promoting healthy lifestyle in Chinese college students: evaluation of a social media-based intervention applying the RE-AIM framework | Reach Effectiveness Adoption Implementation and Maintenance (RE-AIM) |  |
| Fortuna 2023 |  | Patient-centric approach to utilizing visual misperception as a marker for neuropsychiatric disease | Consolidated Framework for Implementation Research (CFIR) |  |
| White 2023 |  | Organizational trust, usability, and inclusivity are key implementation facilitators for a proposed assets-based mobile health intervention | Consolidated Framework for Implementation Research (CFIR) |  |
| Bradford 2021 |  | Optimising symptom management in children with cancer using a novel mobile phone application: protocol for a controlled hybrid effectiveness implementation trial (RESPONSE) | Consolidated Framework for Implementation Research (CFIR)  Reach Effectiveness Adoption Implementation and Maintenance (RE-AIM) | Also includes Expert Recommendation for Implementing Change (ERIC) but not a suitable evaluation framework |
| Glasgow 2021 |  | The NUDGE trial pragmatic trial to enhance cardiovascular medication adherence: study protocol for a randomized controlled trial | Practical, Robust Implementation and Sustainability Model (PRISM) | PRISM includes RE-AIM and adds contextual factors which interact with an intervention to produce RE-AIM outcomes. |
| Lu 2023 |  | Mom's Good Mood: screening and management of perinatal depression within primary healthcare system in China-protocol for an effectiveness-implementation design study | Reach Effectiveness Adoption Implementation and Maintenance (RE-AIM) |  |
| Woulfe 2022 |  | Modification and Validation of an mHealth App Quality Assessment Methodology for International Use: Cross-sectional and eDelphi Studies | Modified Enlight Suite |  |
| Charness 2020 |  | Mobile monitoring and intervention (MMI) technology for adaptive aging | Reach Effectiveness Adoption Implementation and Maintenance (RE-AIM) |  |
| Azad-Khaneghah 2021 |  | Mobile health app usability and quality rating scales: a systematic review | App Quality Evaluation (AQEL)  Unnamed framework by Butcher et al |  |
| Gomm 2022 |  | Mobile Applications Available in Germany Supporting Breast Cancer Patients during Treatment and Aftercare: A Systematic Review | Bespoke evaluation framework for breast cancer apps | Whilst designed for breast cancer, can be readily pivoted to other conditions. |
| Li 2021 |  | Mobile App-Based Intervention for Pregnant Women With Stress Urinary Incontinence: Protocol for a Hybrid Effectiveness-Implementation Trial | Reach Effectiveness Adoption Implementation and Maintenance (RE-AIM) |  |
| Young 2020 |  | A mobile app to capture EPA assessment data: Utilizing the consolidated framework for implementation research to identify enablers and barriers to engagement | Consolidated Framework for Implementation Research (CFIR) |  |
| Chang 2023 |  | Mixed Methods, Implementation Science Evaluation of a Community Health Worker Strategy for HIV Service Engagement in Uganda | Reach Effectiveness Adoption Implementation and Maintenance (RE-AIM) |  |
| Godinho 2020 |  | mHealth for Integrated People-Centred Health Services in the Western Pacific: A Systematic Review | Reach Effectiveness Adoption Implementation and Maintenance (RE-AIM) |  |
| Gladman 2021 |  | Measuring the Quality of Clinical Skills Mobile Apps for Student Learning: Systematic Search, Analysis, and Comparison of Two Measurement Scales | Mobile App Rubric for Learning (MARuL) | Focussed on apps to support learning |
| Pearson 2023 |  | Institute for Clinical and Economic Review - Peterson Health Technology Institute value assessment framework for digital health technologies | Peterson Health Technology Institute value assessment framework for digital health technologies |  |
| Jaffar 2022 |  | Improving Pelvic Floor Muscle Training Adherence Among Pregnant Women: Validation Study | Reach Effectiveness Adoption Implementation and Maintenance (RE-AIM)  APEASE: affordability, practicability, effectiveness and cost-effectiveness, acceptability, side-effects and safety, equity; | APEASE is part of the BCW by Michie et al |
| Siebenhuner 2021 |  | Improvements in Health Might Contradict Adherence to Mobile Health Interventions: Findings from a Self-Care Cancer App Study | Reach Effectiveness Adoption Implementation and Maintenance (RE-AIM) |  |
| Ye 2023 |  | Implications for implementation and adoption of telehealth in developing countries: a systematic review of China's practices and experiences | Consolidated Framework for Implementation Research (CFIR) |  |
| Franzoi 2023 |  | Implementing a PROACTive Care Pathway to Empower and Support Survivors of Breast Cancer | Reach Effectiveness Adoption Implementation and Maintenance (RE-AIM) |  |
| Anderson 2023 |  | Implementing a Community-Led Arsenic Mitigation Intervention for Private Well Users in American Indian Communities: A Qualitative Evaluation of the Strong Heart Water Study Program | Consolidated Framework for Implementation Research (CFIR) |  |
| Cohn 2021 |  | An Implementation Strategy to Expand Mobile Health Use in HIV Care Settings: Rapid Evaluation Study Using the Consolidated Framework for Implementation Research | Consolidated Framework for Implementation Research (CFIR) |  |
| Nelson 2020 |  | Implementation of Technology-Delivered Diabetes Self-care Interventions in Clinical Care: a Narrative Review | Reach Effectiveness Adoption Implementation and Maintenance (RE-AIM)  Consolidated Framework for Implementation Research (CFIR)  Proctor's Implementation Outcomes Framework |  |
| Philpot 2023 |  | Implementation of eLearning solutions for patients with chronic pain conditions | Reach Effectiveness Adoption Implementation and Maintenance (RE-AIM) | CFIR referenced but not used in this instance for evaluation purposes |
| Ellis 2020 |  | Implementation of e-mental health for depression and anxiety: A critical scoping review | Reach Effectiveness Adoption Implementation and Maintenance (RE-AIM)  Consolidated Framework for Implementation Research (CFIR)  Promoting action on research implementation (PARIHS)  Normalisation Process Theory (NPT) | Also includes active implementation framework. Not suitable as not focussed on evaluation. |
| Cho 2023 |  | Implementation of a Scalable Online Weight Management Programme in Clinical Settings: Protocol for the PROPS 2.0 Programme (Partnerships for Reducing Overweight and Obesity with Patient-Centered Strategies 2.0) | Reach, Effectiveness, Adoption, Implementation, and Maintenance (RE-AIM) | RE-AIM selected because authors argue it is well-established and is designed to address issues of implementation and external validity. |
| Smith 2023 |  | Implementation of a culturally competent APOL1 genetic testing programme into living donor evaluation: A two-site, non-randomised, pre-post trial design | Reach Effectiveness Adoption Implementation and Maintenance (RE-AIM) |  |
| Badawy 2021 |  | Implementation and preliminary effectiveness of mHealth apps for improving sickle cell disease care during COVID-19: A mixed-methods evaluation | Reach Effectiveness Adoption Implementation and Maintenance (RE-AIM) |  |
| Ng 2021 |  | Implementation and effectiveness of a multi-domain program for older adults at risk of cognitive impairment at neighborhood senior centres | Reach Effectiveness Adoption Implementation and Maintenance (RE-AIM) |  |
| Badawy 2022 |  | Impact of the COVID-19 Pandemic on the Implementation of Mobile Health to Improve the Uptake of Hydroxyurea in Patients With Sickle Cell Disease: Mixed Methods Study | Reach Effectiveness Adoption Implementation and Maintenance (RE-AIM) |  |
| Edwards 2022 |  | Impact of community health worker intervention on PrEP knowledge and use in Rakai, Uganda: A mixed methods, implementation science evaluation | Reach Effectiveness Adoption Implementation and Maintenance (RE-AIM) |  |
| Alnooh 2023 |  | Identification of the Most Suitable Mobile Apps to Support Dietary Approaches to Stop Hypertension (DASH) Diet Self-Management: Systematic Search of App Stores and Content Analysis | App Quality Evaluation (AQEL)  BCTTv1 |  |
| Scott 2020 |  | Going digital: a narrative overview of the effects, quality and utility of mobile apps in chronic disease self-management | Bespoke evaluation criteria |  |
| Tan 2022 |  | Framework to assess the quality of mHealth apps: a mixed-method international case study protocol | Modified Enlight Suite |  |
| Kleckner 2021 |  | Framework for selecting and benchmarking mobile devices in psychophysiological research | 7-step framework for evaluating mobile devices |  |
| Sit 2021 |  | A Feasibility Study of the WHO Digital Mental Health Intervention Step-by-Step to Address Depression Among Chinese Young Adults | Reach Effectiveness Adoption Implementation and Maintenance (RE-AIM) |  |
| Tsangaris 2023 |  | Feasibility of implementing patient-reported outcome measures into routine breast cancer care delivery using a novel collection and reporting platform | Reach Effectiveness Adoption Implementation and Maintenance (RE-AIM) |  |
| Ming 2023 |  | Feasibility of Implementation of a Mobile Digital Personal Health Record to Coordinate Care for Children and Youth With Special Health Care Needs in Primary Care: Protocol for a Mixed Methods Study | Consolidated Framework for Implementation Research (CFIR) | Also uses technology acceptance model |
| Cordoba 2021 |  | Examining the Information Systems Success (ISS) of a mobile sexual health app (MyPEEPS Mobile) from the perspective of very young men who have sex with men (YMSM) | ISS framework |  |
| ElJoueidi 2021 |  | Evaluation of the implementation process of the mobile health platform 'WelTel' in six sites in East Africa and Canada using the modified consolidated framework for implementation research (mCFIR) | modified consolidated framework for implementation  research (mCFIR) |  |
| Sharma 2022 |  | Evaluation of mHealth Apps for Diverse, Low-Income Patient Populations: Framework Development and Application Study | Bespoke framework to assess strengths and weaknesses of mhealth apps for diverse, low income populations |  |
| Raeesi 2021 |  | Evaluation of HIV/AIDS-related mobile health applications content using an evidence-based content rating tool | Evidence-based content rating tool of mobile health applications (EBCRT-mHealth) | Only one field directly related to HIV/AIDs - potentially adaptable to other conditions. |
| Koziol-McLain 2021 |  | Evaluation of a Healthy Relationship Smartphone App With Indigenous Young People: Protocol for a Co-designed Stepped Wedge Randomized Trial | Reach Effectiveness Adoption Implementation and Maintenance (RE-AIM) |  |
| Niemann 2023 |  | Evaluation criteria for health apps supporting medication adherence in early-stage technology development - a scoping review | BCTTv1  MedAd-AppQ | Framework by Loy identified |
| Ardito 2023 |  | Evaluating Barriers and Facilitators to the Uptake of mHealth Apps in Cancer Care Using the Consolidated Framework for Implementation Research: Scoping Literature Review | Consolidated Framework for Implementation Research (CFIR) |  |
| Carter 2022 |  | Essential Elements to Implementing a Paramedic Palliative Model of Care: An Application of the Consolidated Framework for Implementation Research | Consolidated Framework for Implementation Research (CFIR) |  |
| Franzmair 2021 |  | Effective German and English Language mHealth Apps for Self-management of Bronchial Asthma in Children and Adolescents: Comparison Study | BCT taxonomy |  |
| Qin 2023 |  | Early user experience and lessons learned using ultra-portable digital X-ray with computer-aided detection (DXR-CAD) products: A qualitative study from the perspective of healthcare providers | Consolidated Framework for Implementation Research (CFIR) |  |
| Khan 2023 |  | A "Do No Harm" Novel Safety Checklist and Research Approach to Determine Whether to Launch an Artificial Intelligence-Based Medical Technology: Introducing the Biological-Psychological, Economic, and Social (BPES) Framework | Biological-Psychological, Economic, and Social (BPES) Framework |  |
| Melvin 2022 |  | Dissemination and Implementation of a Google Apple Exposure Notification System for COVID-19 Risk Mitigation at a National Public University: Protocol for a Pilot Evaluation Study in a Real-World Setting | Reach Effectiveness Adoption Implementation and Maintenance (RE-AIM) |  |
| Liem 2020 |  | A digital mental health intervention to reduce depressive symptoms among overseas Filipino workers: protocol for a pilot hybrid type 1 effectiveness-implementation randomized controlled trial | Reach Effectiveness Adoption Implementation and Maintenance (RE-AIM) |  |
| Wagner 2023 |  | Development of an App for Tracking Family Engagement With Early Intervention Services: Focus Groups and Pilot Evaluation Study | Consolidated Framework for Implementation Research (CFIR) |  |
| Ribaut 2024 |  | Developing a Comprehensive List of Criteria to Evaluate the Characteristics and Quality of eHealth Smartphone Apps: Systematic Review | eHealth Smartphone App Evaluation (eHAPPI) framework | Authors acknowledge the criteria is too bulky for regular use. |
| Alon 2023 |  | Current challenges for evaluating mobile health applications | CredibleMind  Framework for evaluating app quality and utility  Health navigator New Zealand  Health-related mobile app evaluation criteria  iMedical | Healthy living Apps score uses other validated scales  Verywellmind is focused on reviewing articles and focused on mental health |
| Sujarwoto 2022 |  | COVID-19 Mobile Health Apps: An Overview of Mobile Applications in Indonesia | WHO guideline recommendations on digital interventions for health system strengthening |  |
| Munoz-Mancisidor 2021 |  | Content, Behavior Change Techniques, and Quality of Pregnancy Apps in Spain: Systematic Search on App Stores | BCTTv1 |  |
| Lee 2023 |  | A content and quality analysis of free, popular mHealth apps supporting 'plant-based' diets | App Quality Evaluation Tool (AQEL) |  |
| McAleese 2020 |  | A content analysis of the quality and behaviour change techniques of smartphone apps promoting the Mediterranean diet | BCTTv1 |  |
| O'Reilly 2023 |  | A Complex mHealth Coaching Intervention to Prevent Overweight, Obesity, and Diabetes in High-Risk Women in Antenatal Care: Protocol for a Hybrid Type 2 Effectiveness-Implementation Study | Reach Effectiveness Adoption Implementation and Maintenance (RE-AIM) |  |
| Hirst 2023 |  | A community-based intervention to improve screening, referral and follow-up of non-communicable diseases and anaemia amongst pregnant and postpartum women in rural India: study protocol for a cluster randomised trial | Reach Effectiveness Adoption Implementation and Maintenance (RE-AIM) |  |
| Ageberg 2024 |  | Co-creating holistic injury prevention training for youth handball: Development of an intervention targeting end-users at the individual, team, and organizational levels | Consolidated Framework for Implementation Research (CFIR) |  |
| Matsuoka 2023 |  | Barriers and facilitators to implementing geriatric assessment in daily oncology practice in Japan: A qualitative study using an implementation framework | Consolidated Framework for Implementation Research (CFIR) |  |
| Meijer 2021 |  | "At least someone thinks I'm doing well": a real-world evaluation of the quit-smoking app StopCoach for lower socio-economic status smokers | Consolidated Framework for Implementation Research (CFIR) |  |
| Stecher 2023 |  | Assessing the Pragmatic Nature of Mobile Health Interventions Promoting Physical Activity: Systematic Review and Meta-analysis | Reach Effectiveness Adoption Implementation and Maintenance (RE-AIM) | Also includes Pragmatic-Explanatory Continuum Indicator Summary-2 (PRECIS-2) which is not suitable as is just a measure of explainability/pragmatism. |
| delaVega 2020 |  | Assessing digital health implementation for a pediatric chronic pain intervention: Comparing the re-aim and bit frameworks against real-world trial data and recommendations for future studies | Reach Effectiveness Adoption Implementation and Maintenance (RE-AIM)  Behavior Interventions using Technology (BIT) |  |
| Grau-Corral 2021 |  | Assessing Apps for Health Care Workers Using the ISYScore-Pro Scale: Development and Validation Study | ISYScore-Pro 17-tem scale |  |
| Fortuna 2023 |  | "As soon as I start trusting human beings, they disappoint me, and now I am going to get on an app that someone could hack. I really do not want to take that chance": barriers and facilitators to digital peer support implementation into community mental health centers | Consolidated Framework for Implementation Research (CFIR) |  |
| Rasmussen 2020 |  | App-Delivered Self-Management Intervention Trial selfBACK for People With Low Back Pain: Protocol for Implementation and Process Evaluation | Reach Effectiveness Adoption Implementation and Maintenance (RE-AIM) |  |
| Baloh 2021 |  | Al-Anon Intensive Referral (AIR): A qualitative formative evaluation for implementation | Consolidated Framework for Implementation Research (CFIR) |  |
| Quimby 2022 |  | Adaptation of a community-based type-2 diabetes mellitus remission intervention during COVID-19: empowering persons living with diabetes to take control | Iterative decision-making for evaluation of adaptations (IDEA) | Another framework was focused on documentation (not suitable)  FRAME-IS (Framework for Reporting Adaptations and Modifications to Evidence-Based Implementation Strategies) |

References:

1. Schliemann D, Ramanathan K, Ibrahim Tamin NSB, O’Neill C, Cardwell CR, Ismail R, et al. Implementation of a colorectal cancer screening intervention in Malaysia (CRC-SIM) in the context of a pandemic: Study protocol. BMJ Open . 2022 Sep;12(9):e058420. Available from: [http://dx.doi.org/10.1136/bmjopen-2021-058420](https://www.google.com/search?q=http://dx.doi.org/10.1136/bmjopen-2021-058420)
2. Rasooly N, Wang D, Luo T, Cheng L, Xue L, Zheng Q, et al. Quality and Performance Measurement in Primary Diabetes Care: A Qualitative Study in Urban^1^ China. Int J Health Policy Manag . 2022 Jun 8; Available from: [http://dx.doi.org/10.34172/ijhpm.2022.6372](https://www.google.com/search?q=http://dx.doi.org/10.34172/ijhpm.2022.6372)
3. MacMillan Uribe A, Houghtaling B, Albrecht J, Fiese B. O25 Evaluation of Commercially Available Infant Feeding Mobile Applications Using the App Quality Evaluation Tool. J Nutr Educ Behav . 2022 Jul;54(7):S23. Available from: [http://dx.doi.org/10.1016/j.jneb.2022.04.032](https://www.google.com/search?q=http://dx.doi.org/10.1016/j.jneb.2022.04.032)
4. Di Sebastiano K, Chulak-Bozzer T, Vanderloo L, Faulkner G. An Evaluation of a Commercialized mHealth Intervention to Promote Physical Activity in the Workplace. Front Public Health . 2022 Apr 26;10:740350. Available from: [http://dx.doi.org/10.3389/fpubh.2022.740350](https://www.google.com/search?q=http://dx.doi.org/10.3389/fpubh.2022.740350)
5. Bu D, Chung S, Kang D, Lee J, Tran B, Lee E, et al. Optimising implementation of telehealth in oncology: A systematic review examining barriers and enablers using the RE-AIM planning and evaluation framework. Crit Rev Oncol Hematol . 2022 Dec;180:103869. Available from: [http://dx.doi.org/10.1016/j.critrevonc.2022.103869](https://www.google.com/search?q=http://dx.doi.org/10.1016/j.critrevonc.2022.103869)
6. Bernard P, Romain AJ, Vancampfort D, Baillot A, Eskenazi M, Lennis L, et al. Strategies for Implementing Occupational eMental Health Interventions: Scoping Review. J Med Internet Res . 2022 Feb 28;24(2):e34479. Available from: [http://dx.doi.org/10.2196/34479](https://www.google.com/search?q=http://dx.doi.org/10.2196/34479)
7. Ko H, Lim J, Choi J, Lee K. The Development of a Mobile Application for Older Adults for Rehabilitation Instructions After Hip Fracture Surgery. Glob Adv Health Med . 2021 Apr 19;10:21514593211006693. Available from: [http://dx.doi.org/10.1177/21514593211006693](https://www.google.com/search?q=http://dx.doi.org/10.1177/21514593211006693)
8. Garvin LA, Hu J, Slightam C, Zulman D, Blonigen D, Asch S, et al. Use of Video Telehealth Tablets to Increase Access for Veterans Experiencing Homelessness. J Gen Intern Med . 2021 Oct;36(10):3069–76. Available from: [http://dx.doi.org/10.1007/s11606-021-06900-8](https://www.google.com/search?q=http://dx.doi.org/10.1007/s11606-021-06900-8)
9. Mosch N, Coleman J, Reed M, Jones D, Thompson H, Holder A, et al. Evaluating the Implementation of a Remote Patient Monitoring Platform in the Intensive Care Unit: Qualitative Study. J Med Internet Res . 2020 Oct 23;22(10):e22866. Available from: [http://dx.doi.org/10.2196/22866](https://www.google.com/search?q=http://dx.doi.org/10.2196/22866)
10. Johnson C, Davis T, Shynett K, Carter J, Hardee S, Jackson J, et al. Engaging Parents in Education for Discharge (ePED): Evaluating the Reach, Adoption & Implementation of an innovative discharge teaching method. J Pediatr Nurs . 2020 Nov;55:238–44. Available from: [http://dx.doi.org/10.1016/j.pedn.2020.05.022](https://www.google.com/search?q=http://dx.doi.org/10.1016/j.pedn.2020.05.022)
11. Furlonger B, Anderson A, Snell T. Ways in which school psychologists can identify suitable apps for supporting the self-management of asthma by students. Educational and Developmental Psychologist . 2021;38(1):110–6. Available from: [http://dx.doi.org/10.1017/edp.2020.3](https://www.google.com/search?q=http://dx.doi.org/10.1017/edp.2020.3)
12. Denecke K, Warren J. How to Evaluate Health Applications with Conversational User Interface? Stud Health Technol Inform . 2020 Jun 16;270:976–80. Available from: <http://dx.doi.org/10.3233/SHTI200307>
13. Avdagovska M, Stanimirovic D, Kuziemsky C. Capturing the Impact of Patient Portals Based on the Quadruple Aim and Benefits Evaluation Frameworks: Scoping Review. J Med Internet Res . 2020 Dec 16;22(12):e24568. Available from: <http://dx.doi.org/10.2196/24568>
14. Steigerwalt K, Persuitte G, Miller C, Jones L, Powell J. P3049 A Novel Mobile App’s Reliability, End User Satisfaction, and Changes in Dash Diet Eating Patterns Over 8 Weeks. Hypertension . 2019 Sep 1;74(Suppl_1). Available from: [http://dx.doi.org/10.1161/hyp.74.suppl_1.p3049](https://www.google.com/search?q=http://dx.doi.org/10.1161/hyp.74.suppl_1.p3049)
15. Liaw S-T, Kuziemsky C, Schreiber R, Jonnagaddala J. Use of mHealth for promoting healthy ageing and supporting delivery of age-friendly care services: a systematic review. International Journal of Integrated Care . 2019 Dec 18;19(S1). Available from: <http://dx.doi.org/10.5334/ijic.s3147>
16. Rogers E, Mizrachi J, Ramirez V, Hughes A, Sperling J, Young K, et al. Barriers and Facilitators to the Implementation of a Mobile Insulin Titration Intervention for Patients With Uncontrolled Diabetes: A Qualitative Analysis. JMIR Diabetes . 2019 Mar 26;4(1):e13906. Available from: [http://dx.doi.org/10.2196/13906](https://www.google.com/search?q=http://dx.doi.org/10.2196/13906)
17. Koot D, Goh P-S, Lim R, Yau T, Ali S, Shabbir A, et al. A Mobile Lifestyle Management Program (GlycoLeap) for People With Type 2 Diabetes: Single-Arm Feasibility Study. JMIR Mhealth Uhealth . 2019 May 23;7(5):e12965. Available from: [http://dx.doi.org/10.2196/12965](https://www.google.com/search?q=http://dx.doi.org/10.2196/12965)
18. Ide C, Paige S, Zimba C, Smith M, van der Putten M, Bennett J. People welcomed this innovation with two hands: A qualitative report of an mhealth intervention for community case management in Malawi. Annals of Global Health . 2019 Mar 14;85(1). Available from: [http://dx.doi.org/10.5334/aogh.919](https://www.google.com/search?q=http://dx.doi.org/10.5334/aogh.919)
19. Hay-Smith J, McClurg D, Frawley H, Dean S. Apps-olutely fabulous?-the quality of pfmt smartphone app content and design rated using the mobile app rating scale, behaviour change taxonomy, and guidance for exercise prescription. Neurourol Urodyn. 2019 Nov;38 Suppl 4:S3.
20. Felix L, Sukunesan S, McLean G, Rodgers S, Semple M, Cheek L, et al. Development of a Complex Intervention to Improve Adherence to Antidiabetic Medication in Older People Using an Anthropomorphic Virtual Assistant Software. Front Pharmacol . 2019 Jun 25;10:680. Available from: [http://dx.doi.org/10.3389/fphar.2019.00680](https://www.google.com/search?q=http://dx.doi.org/10.3389/fphar.2019.00680)
21. Blok A, Carpenter M, Cohn A, Wilson D, Errante A, Gray K, et al. Nurse-Driven mHealth Implementation Using the Technology Inpatient Program for Smokers (TIPS): Mixed Methods^2^ Study. JMIR Mhealth Uhealth . 2019 Nov 19;7(11):e14331. Available from: [http://dx.doi.org/10.2196/14331](https://www.google.com/search?q=http://dx.doi.org/10.2196/14331)
22. Arrossi S, Paolino M, Orellana L, Thouyaret L, Kohler RE, Viswanath K. Mixed-methods approach to evaluate an mHealth intervention to increase adherence to triage of human papillomavirus–positive women who have performed self-collection (the ATICA study): study protocol for a hybrid type I cluster randomized effectiveness–implementation trial. Trials . 2019 Mar 14;20(1):180. Available from: [http://dx.doi.org/10.1186/s13063-019-3229-3](https://www.google.com/search?q=http://dx.doi.org/10.1186/s13063-019-3229-3)
23. Stockner M, Spitzer B, Rappaport D, Kellner C, Sharma R, Koenig J, et al. (P1.212) Using the RE-AIM Framework in Formative Evaluation/Planning of a Mobile Prehospital Telestroke Intervention in an Urban Setting: Pilot Data for the Prehospital Rapid Evaluation via Ambulance Lead Emergency Remote Telemedicine (PRE-ALERT) Study. Neurology . 2018 Apr 1;90(15 Supplement). Available from: [http://dx.doi.org/10.1212/wnl.90.15_supplement.p1.212](https://www.google.com/search?q=http://dx.doi.org/10.1212/wnl.90.15_supplement.p1.212)
24. Lakerveld J, Mackenbach JD, Horvath E, Rutters F, Compernolle S, Bárdos H, et al. Improving cardiometabolic health through nudging dietary behaviours and physical activity in low SES adults: design of the Supreme Nudge project. BMC Public Health . 2018 Aug 16;18(1):1020. Available from: <http://dx.doi.org/10.1186/s12889-018-5839-1>^3^
25. Xie B, Su Z, Zhang W, Cai R. Chinese Cardiovascular Disease Mobile Apps’ Information Types, Information Quality, and Interactive Functions for Self-Management: Systematic Review. JMIR Mhealth Uhealth . 2017 Dec 14;5(12):e195. Available from: [http://dx.doi.org/10.2196/mhealth.8549](https://www.google.com/search?q=http://dx.doi.org/10.2196/mhealth.8549)
26. Tinschert P, Jakob R, Barata F, Kramer J, Kowatsch T. The Potential of Mobile Apps for Improving Asthma Self-Management: A Review of Publicly Available and Well-Adopted Asthma Apps. JMIR Mhealth Uhealth . 2017 Aug 2;5(8):e113. Available from: <http://dx.doi.org/10.2196/mhealth.7177>
27. Sapru S, Sankar PS, Gogate P, Singh K, Motukupally SR, Posnett KR, et al. Applying RE-AIM to evaluate two community-based programs designed to improve access to eye care for those at high-risk for glaucoma. Eval Program Plann . 2017 Oct;64:117–24. Available from: [http://dx.doi.org/10.1016/j.evalprogplan.2017.06.006](https://www.google.com/search?q=http://dx.doi.org/10.1016/j.evalprogplan.2017.06.006)
28. Mehmood A, Saqib G, Laflamme L, Razzak J. Development of an mHealth trauma registry in the Middle East using an implementation science framework. Glob Health Action . 2017 Sep 29;10(1):1380360. Available from: [http://dx.doi.org/10.1080/16549716.2017.1380360](https://www.google.com/search?q=http://dx.doi.org/10.1080/16549716.2017.1380360)
29. Vallespin B, Macedo J, Rose L. Ensuring Evidence-Based Safe and Effective mHealth Applications. Stud Health Technol Inform. 2016;225:248–52.
30. Garg S, Friebel R, Car J, Basu S, Kapur A. Qualitative analysis of programmatic initiatives to text patients with mobile devices in resource-limited health systems. BMC Med Inform Decis Mak . 2016 Feb 26;16(1):23. Available from: <http://dx.doi.org/10.1186/s12911-016-0258-7>
31. Panda N, Lee H, Chen J, Chung P, Law C, Coburn N, et al. Perceptions of Mobile Health Technology in Elective Surgery: A Qualitative Study of North American Surgeons. Ann Surg . 2023 Aug 1;278(2):e392–400. Available from: [http://dx.doi.org/10.1097/sla.0000000000005208](https://www.google.com/search?q=http://dx.doi.org/10.1097/sla.0000000000005208)
32. MacMillan Uribe A, Houghtaling B, Albrecht J, Fiese B. Appropriateness and Relevance of Infant Feeding Mobile Applications for Lesbian, Gay, Bisexual, Transgender, or Queer Mothers Society for Nutrition Education and Behavior (SNEB), 55th Annual Conference, July 20-23, 2023, Washington, DC. J Nutr Educ Behav . 2023 Jul;55(7):S106. Available from: [http://dx.doi.org/10.1016/j.jneb.2023.05.134](https://www.google.com/search?q=http://dx.doi.org/10.1016/j.jneb.2023.05.134)
33. Khalid F, Spruill T, Crowe J, Ogedegbe O, Schoenthaler A. Implementation Science Perspectives on Implementing Telemedicine Interventions for Hypertension or Diabetes Management: Scoping Review. JMIR Form Res . 2023 Feb 10;7:e42134. Available from: [http://dx.doi.org/10.2196/42134](https://www.google.com/search?q=http://dx.doi.org/10.2196/42134)
34. Cox N, McDonald C, Alison J, Mahal A, Wootton S, Hill K, et al. Telerehabilitation in the ‘Real World’: Implementation of Remotely Delivered Pulmonary Rehabilitation. Am J Respir Crit Care Med . 2023 May 1;207(9):A4676–A4676. Available from: [http://dx.doi.org/10.1164/ajrccm-conference.2023.207.1_meetingabstracts.a4676](https://www.google.com/search?q=http://dx.doi.org/10.1164/ajrccm-conference.2023.207.1_meetingabstracts.a4676)
35. Cox N, McDonald C, Alison J, Mahal A, Wootton S, Hill K, et al. Telerehabilitation in the ‘real-world’: Implementation of remotely delivered pulmonary rehabilitation. Am J Respir Crit Care Med . 2023 May 1;207(9):A4676–A4676. Available from: [http://dx.doi.org/10.1164/ajrccm-conference.2023.207.1_meetingabstracts.a4676](https://www.google.com/search?q=http://dx.doi.org/10.1164/ajrccm-conference.2023.207.1_meetingabstracts.a4676)
36. Brill P, McCarthy J, Howard T, Davis N, Lee B, Conrey E, et al. Implementing the Better Starts For All Pilot Mobile and Telehealth Intervention in Ohio Appalachia: Improving Access to Maternal Healthcare. Matern Child Health J . 2024 Jan;28(1):204–12. Available from: [http://dx.doi.org/10.1007/s10995-023-03808-2](https://www.google.com/search?q=http://dx.doi.org/10.1007/s10995-023-03808-2)
37. Aydin E, Güner Ş, Çelebi B, Alataş N, Aladağ E, Özdamar İ, et al. Mobile care app development process: using the ADDIE model to manage symptoms after breast cancer surgery (step 1). Support Care Cancer . 2023 Apr 19;31(5):275. Available from: [http://dx.doi.org/10.1007/s00520-023-07707-5](https://www.google.com/search?q=http://dx.doi.org/10.1007/s00520-023-07707-5)
38. Youn SJ, Constantino MI, Shiner B, Watts B V., Schnurr PP. Leveraging Implementation Science to Integrate Digital Mental Health Interventions as Part of Routine Care in a Practice Research Network. Adm Policy Ment Health . 2023 Jul;50(4):631–43. Available from: [http://dx.doi.org/10.1007/s10488-023-01292-9](https://www.google.com/search?q=http://dx.doi.org/10.1007/s10488-023-01292-9)
39. Straw I, Orellana L, Paolino M, Pesce E, Viswanath K, Arrossi S. Implementation and scaling-up of an effective mHealth intervention to increase adherence to triage of HPV-positive women (ATICA study): perceptions of health decision-makers and health-care providers. BMC Health Serv Res . 2023 Jan 17;23(1):54. Available from: [http://dx.doi.org/10.1186/s12913-023-09022-5](https://www.google.com/search?q=http://dx.doi.org/10.1186/s12913-023-09022-5)
40. Sharma A, Landman A, Bates DW. A systematic review assessing the state of analytical validation for connected, mobile, sensor-based digital health technologies. medRxiv . 2023 May 23; Available from: [http://dx.doi.org/10.1101/2023.05.22.23290371](https://www.google.com/search?q=http://dx.doi.org/10.1101/2023.05.22.23290371)
41. Lundström S, Hesser H, Andersson G, Fernström M, Johansson M, Rück C, et al. Effectiveness of Internet-based cognitive-behavioural therapy for obsessive-compulsive disorder (OCD-NET) and body dysmorphic disorder (BDD-NET) in the Swedish public health system using the RE-AIM implementation framework. Internet Interv . 2023 Dec;34:100608. Available from: [http://dx.doi.org/10.1016/j.invent.2023.100608](https://www.google.com/search?q=http://dx.doi.org/10.1016/j.invent.2023.100608)
42. Li Y, Wang R, Xie H, Zheng H, Peng P. Barriers and facilitators of implementing electronic monitors to improve adherence and health outcomes in tuberculosis patients: protocol for a systematic review based on the Consolidated Framework for Implementation Research. Health Res Policy Syst . 2023 Oct 26;21(1):117. Available from: [http://dx.doi.org/10.1186/s12961-023-01054-x](https://www.google.com/search?q=http://dx.doi.org/10.1186/s12961-023-01054-x)
43. LeLaurin J, Hughes AE, Porter M, Staras SAS, Cottler LB, Thompson LA, et al. Pediatric primary care provider and staff perspectives on the implementation of electronic health record-based social needs interventions: A mixed-methods study. J Clin Transl Sci . 2023;7(1):e163. Available from: [http://dx.doi.org/10.1017/cts.2023.585](https://www.google.com/search?q=http://dx.doi.org/10.1017/cts.2023.585)
44. Knapp M, Schleider JL, Towne S, Lindell L, Lipkin C, Merchant RM, et al. “The library is so much more than books”: considerations for the design and implementation of teen digital mental health services in public libraries. Front Digit Health . 2023 Jul 5;5:1183319. Available from: [http://dx.doi.org/10.3389/fdgth.2023.1183319](https://www.google.com/search?q=http://dx.doi.org/10.3389/fdgth.2023.1183319)
45. Holloway BM, Peterson RA, Fischer LE, Smith JL, Dietz LR, Fisher EB, et al. Low-Intensity mental health Support via a Telehealth Enabled Network for adults with diabetes (LISTEN): protocol for a hybrid type 1 effectiveness implementation trial. Trials . 2023 Jun 15;24(1):398. Available from: [http://dx.doi.org/10.1186/s13063-023-07338-5](https://www.google.com/search?q=http://dx.doi.org/10.1186/s13063-023-07338-5)
46. Hailemariam M, Assefa T, Tilahun B, Mirelman A, Kebede Z, Assefa Y. Individual and contextual level enablers and barriers determining electronic community health information system implementation in northwest Ethiopia. BMC Health Serv Res . 2023 Jun 26;23(1):671. Available from: [http://dx.doi.org/10.1186/s12913-023-09629-8](https://www.google.com/search?q=http://dx.doi.org/10.1186/s12913-023-09629-8)
47. Gisondi M, Papanagnou D, Carney K, Chen M, Cohen A, Giordano C, et al. Teaching LGBTQ+ Health, a Web-Based Faculty Development Course: Program Evaluation Study Using the RE-AIM Framework. JMIR Med Educ . 2023 Oct 16;9:e47777. Available from: [http://dx.doi.org/10.2196/47777](https://www.google.com/search?q=http://dx.doi.org/10.2196/47777)
48. Gamble A, Allen JD, Patel A, Nelson J, Thomas ML, Peterson RA, et al. Telehealth Diabetes Prevention Program for Adults With Prediabetes in an Academic Medical Center Setting: Protocol for a Hybrid Type III Trial. JMIR Res Protoc . 2023 Oct 20;12:e50183. Available from: [http://dx.doi.org/10.2196/50183](https://www.google.com/search?q=http://dx.doi.org/10.2196/50183)
49. Freund T, Terhorst Y, Gensichen J, Zerth J. Using the Consolidated Framework for Implementation Research to evaluate a nationwide depression prevention project (ImplementIT) from the perspective of health care workers and implementers: Results on the implementation of digital interventions for farmers. Front Digit Health . 2023 Jan 6;4:1083143. Available from: [http://dx.doi.org/10.3389/fdgth.2022.1083143](https://www.google.com/search?q=http://dx.doi.org/10.3389/fdgth.2022.1083143)
50. Bagsic R, Sharif M, Garcia G, Albright C, Chow P, Garcia J, et al. Process evaluation of Dulce Digital-Me: an adaptive mobile health (mHealth) intervention for underserved Hispanics with diabetes. Transl Behav Med . 2023 Jun 14;13(6):403–12. Available from: [http://dx.doi.org/10.1093/tbm/ibad020](https://www.google.com/search?q=http://dx.doi.org/10.1093/tbm/ibad020)
51. Yudkin JS, Nigatu T, Okubatsion G, Verguet S, Davies JI, Beran D. Needs Assessment and Best Practices for Digital Trainings for Health Professionals in Ethiopia Using the RE-AIM Framework: COVID-19, Case Study. Disaster Med Public Health Prep . 2022 Nov 9;17:e68. Available from: [http://dx.doi.org/10.1017/dmp.2022.224](https://www.google.com/search?q=http://dx.doi.org/10.1017/dmp.2022.224)
52. Stump T, Benbow N, Ojikutu B, Psihogios A, Hitsman B, Brown C, et al. Development of an Implementation Facilitation Strategy to Link Mental Health Screening and eHealth Intervention for Clients in Ryan White-Funded Clinics in Chicago. J Acquir Immune Defic Syndr . 2022 Aug 1;90(4):391–9. Available from: [http://dx.doi.org/10.1097/qai.0000000000002980](https://www.google.com/search?q=http://dx.doi.org/10.1097/qai.0000000000002980)
53. Simon K, Jordan B, Schwartz L, Baker J, Weaver M, Schwartz D, et al. Digital health interventions for pain in pediatric oncology: state of the field. Support Care Cancer . 2023 Mar 9;31(4):208. Available from: [http://dx.doi.org/10.1007/s00520-023-07629-2](https://www.google.com/search?q=http://dx.doi.org/10.1007/s00520-023-07629-2)
54. Mosch N, Coleman J, Reed M, Jones D, Thompson H, Holder A, et al. Creation of an Evidence-Based Implementation Framework for Digital Health Technology in the Intensive Care Unit: Qualitative Study. JMIR Form Res . 2022 Apr 27;6(4):e34032. Available from: [http://dx.doi.org/10.2196/34032](https://www.google.com/search?q=http://dx.doi.org/10.2196/34032)
55. Mantri SB, Agarwal V, Lakhotia N, Sahu S, Kumar A, Sharma A, et al. Assessment of e-aushadhi program (drug inventory e-health initiative in Rajasthan) using benefit evaluation framework. J Family Med Prim Care . 2022 Feb;11(2):746–53. Available from: <http://dx.doi.org/10.4103/jfmpc.jfmpc_2047_21>
56. Holt L, Unertl K, Johnson K, Lorenzi N. Real-world implementation evaluation of an electronic health record-integrated consumer informatics tool that collects patient-generated contextual data. Int J Med Inform . 2022 Aug;164:104810. Available from: [http://dx.doi.org/10.1016/j.ijmedinf.2022.104810](https://www.google.com/search?q=http://dx.doi.org/10.1016/j.ijmedinf.2022.104810)
57. Hodges JE, Horvath KJ, Castel AD, Williams K, Yoon C, Levy ME, et al. Evaluation of the Implementation and Effectiveness of a Mobile Health Intervention to Improve Outcomes for People With HIV in the Washington, DC Cohort: Study Protocol for a Cluster Randomized Controlled Trial. JMIR Res Protoc . 2022 May 27;11(5):e37748. Available from: [http://dx.doi.org/10.2196/37748](https://www.google.com/search?q=http://dx.doi.org/10.2196/37748)
58. Dwyer A, Indyk J, Leo J, Agius J, Lin A, Yong A, et al. Navigating Disrupted Puberty: Development and Evaluation of a Mobile-Health Transition Passport for Klinefelter Syndrome. Front Endocrinol (Lausanne) . 2022 May 26;13:909830. Available from:^4^ [http://dx.doi.org/10.3389/fendo.2022.909830](https://www.google.com/search?q=http://dx.doi.org/10.3389/fendo.2022.909830)
59. Castor F, Safaeinili N, Li J, Jacobson S, Raman R, Parham G, et al. Assessment of the implementation context in preparation for a clinical study of machine-learning algorithms^5^ to automate the classification of digital cervical images for cervical cancer screening in resource-constrained settings. Front Reprod Health . 2022 Nov 1;4:1000150. Available from: [http://dx.doi.org/10.3389/frhs.2022.1000150](https://www.google.com/search?q=http://dx.doi.org/10.3389/frhs.2022.1000150)
60. Burton J, Regala S, Williams D, Desai A, He H, Aalami O, et al. A Comparative Utility Score for Digital Health Tools. J Med Syst . 2022 May 5;46(6):34. Available from: [http://dx.doi.org/10.1007/s10916-022-01821-3](https://www.google.com/search?q=http://dx.doi.org/10.1007/s10916-022-01821-3)
61. Yoshida Y, Boren SA, Soares J, Popescu M, Nielson SD, Simoes EJ, et al. Using the RE-AIM framework to evaluate internal and external validity of mobile phone-based interventions in diabetes self-management education and support. J Am Med Inform Assoc . 2020 Jul 1;27(7):1131–40. Available from: [http://dx.doi.org/10.1093/jamia/ocaa041](https://www.google.com/search?q=http://dx.doi.org/10.1093/jamia/ocaa041)
62. van Reijen M, Vriend I, van Mechelen W, Finch C, Verhagen E. Users’ Perspectives, Opportunities, and Barriers of the Strengthen Your Ankle App for Evidence-Based Ankle Sprain Prevention: Mixed-Methods Process Evaluation for a Randomized Controlled Trial. JMIR Rehabil Assist Technol . 2018 Jan 12;5(1):e1. Available from: [http://dx.doi.org/10.2196/rehab.8638](https://www.google.com/search?q=http://dx.doi.org/10.2196/rehab.8638)
63. Chapel L, Leboul D, Bertuit J, Perrochon A. Standardization of the assessment process within telerehabilitation in chronic diseases: a scoping meta-review. BMC Health Serv Res . 2022 Aug 27;22(1):1092. Available from: [http://dx.doi.org/10.1186/s12913-022-08370-y](https://www.google.com/search?q=http://dx.doi.org/10.1186/s12913-022-08370-y)
64. Yu C, Srgo K, Srgo E, Straus S, Lovblom L, Halpern E, et al. Process Evaluation of the Diabetes Canada Guidelines Dissemination Strategy Using the Reach Effectiveness Adoption Implementation Maintenance (RE-AIM) Framework. Can J Diabetes . 2019 Aug;43(6):376-383.e1. Available from: [http://dx.doi.org/10.1016/j.jcjd.2018.08.189](https://www.google.com/search?q=http://dx.doi.org/10.1016/j.jcjd.2018.08.189)
65. Martin Payo R, Grande Tejada AM, Garcia Criado EI, Fernandez Perez A, Garcia Ortiz L, Armisén Borrego M, et al. Prescribing fitness apps for people with cancer: a preliminary assessment of content and quality of commercially available apps. Int J Behav Nutr Phys Act . 2019 Jun 13;16(1):49. Available from: [http://dx.doi.org/10.1186/s12966-019-0808-7](https://www.google.com/search?q=http://dx.doi.org/10.1186/s12966-019-0808-7)
66. Bardosh K, Abu-Odah H, Shakil A, Clark H, Murray M, Smillie K, et al. Operationalizing mHealth to improve patient care: A qualitative implementation science evaluation of the WelTel texting intervention in Canada and Kenya. Global Health . 2017 Dec 2;13(1):87. Available from: <http://dx.doi.org/10.1186/s12992-017-0311-z>
67. DiFilippo K, Huang W, Chapman-Novakofski K. A New Tool for Nutrition App Quality Evaluation (AQEL): Development, Validation, and Reliability Testing. JMIR Mhealth Uhealth . 2017 Oct 27;5(10):e163. Available from: <http://dx.doi.org/10.2196/mhealth.7441>
68. DiFilippo K, Huang W-HD, Chapman-Novakofski K. Mobile Apps for the Dietary Approaches to Stop Hypertension (DASH): App Quality Evaluation. J Nutr Educ Behav . 2018 Jul;50(7):620–5. Available from: [http://dx.doi.org/10.1016/j.jneb.2018.02.002](https://www.google.com/search?q=http://dx.doi.org/10.1016/j.jneb.2018.02.002)
69. Lehmann NJ, Weber L, Hoffmann R, Möckel M, Slagman A, Schmieding ML. mHealthAtlas - An approach for the multidisciplinary evaluation of mHealth applications. 2020 IEEE Int Conf E-health Netw Appl Serv HEALTHCOM . 2021 Mar 1;1–5. Available from: [http://dx.doi.org/10.1109/healthcom49281.2021.9399045](https://www.google.com/search?q=http://dx.doi.org/10.1109/healthcom49281.2021.9399045)
70. Fiore M, Triberti S, Graziani F, Pravettoni G, Lombi L. How to Evaluate Mobile Health Applications: A Scoping Review. Stud Health Technol Inform. 2017;235:109–14.
71. Sadegh SS, Saadat PK, Sepehri MM, Assadi V. A framework for m-health service development and success evaluation. Int J Med Inform . 2018 Apr;112:123–30. Available from: [http://dx.doi.org/10.1016/j.ijmedinf.2018.01.003](https://www.google.com/search?q=http://dx.doi.org/10.1016/j.ijmedinf.2018.01.003)
72. Capraş RD, Bolboacă SD. An Evaluation of Free Medical Applications for Android Smartphones. Appl Med Inform . 2016 Dec 29 [cited 2024 Apr 23];38(3-4):117–32. Available from: <https://ami.info.umfcluj.ro/index.php/AMI/article/view/608>
73. McKay FH, Wright A, Shill J, Stephens H, Vandelanotte C. Evaluating mobile phone applications for health behaviour change: A systematic review. Health Informatics J . 2019 Jun;25(2):289–304. Available from: [http://dx.doi.org/10.1177/1460458217738713](https://www.google.com/search?q=http://dx.doi.org/10.1177/1460458217738713)
74. Turnbull T, Suthakorn W, Boonbrahm S. Development of OSOMO Prompt Mobile Application on Elderly Population for Village Health Volunteers Using the Analysis, Design, Development, Implementation, and Evaluation (ADDIE) Model. Stud Health Technol Inform . 2023 Jun 29;305:404–7. Available from: [http://dx.doi.org/10.3233/shti230107](https://www.google.com/search?q=http://dx.doi.org/10.3233/shti230107)
75. Saeidnia H, Khoei A, Saeidi M, Kozak M, Ghazisaeedi M, Maghrebi S, et al. Development of a Mobile App for Self-Care Against COVID-19 Using the Analysis, Design, Development, Implementation, and Evaluation (ADDIE) Model: Methodological Study. JMIR Form Res . 2022 May 17;6(5):e39718. Available from: <http://dx.doi.org/10.2196/39718>
76. Son S, Lee H, Kim H, Kim H, Park C, Cho E. Development and evaluation of a tailored mHealth parenting program for multicultural families: a three-arm cluster randomized controlled trial. Front Public Health . 2023 Jul 13;11:1182310. Available from: [http://dx.doi.org/10.3389/fpubh.2023.1182310](https://www.google.com/search?q=http://dx.doi.org/10.3389/fpubh.2023.1182310)
77. White BK, Lubans DR, Bennie JA, Anderson A, Eather N. Designing evaluation plans for health promotion mHealth interventions: a case study of the Milk Man mobile app. Health Promot Int . 2018 Apr 1;33(2):296–306. Available from: [http://dx.doi.org/10.1093/heapro/daw095](https://www.google.com/search?q=http://dx.doi.org/10.1093/heapro/daw095)
78. Torquati L, Mielke GI, Brown WJ, Burton NW, Kolbe-Alexander TL. Changing Diet and Physical Activity in Nurses: A Pilot Study and Process Evaluation Highlighting Challenges in Workplace Health Promotion. J Nutr Educ Behav . 2018 Mar;50(3):276-285.e1. Available from: [http://dx.doi.org/10.1016/j.jneb.2017.12.001](https://www.google.com/search?q=http://dx.doi.org/10.1016/j.jneb.2017.12.001)
79. Grau I, Kostov B, Gallego JA, Grajales FJ, Sisó-Almirall A. Assessment method for mobile health applications in Spanish: The iSYScore index. Semergen . 2016 Oct;42(7):460–7. Available from: [http://dx.doi.org/10.1016/j.semerg.2015.12.001](https://www.google.com/search?q=http://dx.doi.org/10.1016/j.semerg.2015.12.001)
80. Wang Y, Zhou Y, Feng R, Zhuang Y, Min Q, Dong W, et al. An analysis and evaluation of quality and behavioral change techniques among physical activity apps in China. Int J Med Inform . 2020 Jan;133:104029. Available from: [http://dx.doi.org/10.1016/j.ijmedinf.2019.104029](https://www.google.com/search?q=http://dx.doi.org/10.1016/j.ijmedinf.2019.104029)
81. Woulfe F, O’Donoghue J, O’Grady M. Identification and Evaluation of Methodologies to Assess the Quality of Mobile Health Apps in High-, Low-, and Middle-Income Countries: Rapid Review. JMIR Mhealth Uhealth . 2021 May 28;9(5):e28384. Available from: [http://dx.doi.org/10.2196/28384](https://www.google.com/search?q=http://dx.doi.org/10.2196/28384)
82. Wang X, Du K, Zhu K, Xu S, Zhang S. Where Should Mobile Health Application Providers Focus Their Goals? International Journal of Computational Intelligence Systems . 2021 Mar 1;14(1):1030. Available from: [http://dx.doi.org/10.2991/ijcis.d.210305.001](https://www.google.com/search?q=http://dx.doi.org/10.2991/ijcis.d.210305.001)
83. Spencer-Brown C, Wilcox S, Levine E, Griffin S, Adds M, Tinkley K, et al. Evaluation of an Electronic Health Record Referral Process to Enhance Participation in Evidence-Based Arthritis Interventions. Prev Chronic Dis . 2021 Apr 8;18:E34. Available from: [http://dx.doi.org/10.5888/pcd18.200484](https://www.google.com/search?q=http://dx.doi.org/10.5888/pcd18.200484)
84. Reisinger-Kindle K, Hangsleben A, Rodriguez N, Solomon A, Rankin K, Linden A, et al. Evaluation of rapid telehealth implementation for prenatal and postpartum care visits during the COVID-19 pandemic in an academic clinic in Springfield, Massachusetts, United States of America. Health Sci Rep . 2021 Dec;4(4):e455. Available from: [http://dx.doi.org/10.1002/hsr2.455](https://www.google.com/search?q=http://dx.doi.org/10.1002/hsr2.455)
85. Pach JD, Dabrock P, Fegeler C, Bergmann S, Albrecht UV. Five Lessons Learned From Randomized Controlled Trials on Mobile Health Interventions: Consensus Procedure on Practical Recommendations for Sustainable Research. JMIR Mhealth Uhealth . 2021 Apr 7;9(4):e20630. Available from: <http://dx.doi.org/10.2196/20630>
86. Joshi A, Amadi C, Bloom T, Mastan M, Joshi M. Approaches to Assess E-Health Programs: A Scoping Review. Indian J Community Med . 2021;46(1):12–7. Available from: [http://dx.doi.org/10.4103/ijcm.ijcm_340_20](https://www.google.com/search?q=http://dx.doi.org/10.4103/ijcm.ijcm_340_20)
87. Halminen O, Wan D, Haga S, Brørs G, Andreassen H, Fidjeland A. Demonstrating the value of digital health: Guidance on contextual evidence gathering for companies in different stages of maturity. Health Informatics J . 2021 Jan;27(1):1460458220971447. Available from: [http://dx.doi.org/10.1177/1460458220971447](https://www.google.com/search?q=http://dx.doi.org/10.1177/1460458220971447)
88. Gong E, Gu W, Zhang Z, Chen Y, Wu X, Wang X, et al. The Implementation of a Primary Care-Based Integrated Mobile Health Intervention for Stroke Management in Rural China: Mixed-Methods Process Evaluation. Front Public Health . 2021 Dec 16;9:774907. Available from: [http://dx.doi.org/10.3389/fpubh.2021.774907](https://www.google.com/search?q=http://dx.doi.org/10.3389/fpubh.2021.774907)
89. Daniels M, Schaffer K, Lin A, Blanchard J, McIntyre B, Losonczy L. 77 Using Mobile Integrated Health and Telehealth to Support Transition of Care Amond Heart Failure Patients: Mighty Heart Study Protocol. Ann Emerg Med . 2021 Oct;78(4):S32. Available from: [http://dx.doi.org/10.1016/j.annemergmed.2021.09.086](https://www.google.com/search?q=http://dx.doi.org/10.1016/j.annemergmed%3C5%3E.2021.09.086)
90. Beauchemin M, Schmidt M, Banegas M, Emerson N, Leader A, Flannery M, et al. Incorporating systematic financial screening into the electronic health record. J Clin Oncol . 2021 Oct 1;39(28_suppl):18–18. Available from: [http://dx.doi.org/10.1200/jco.2020.39.28_suppl.18](https://www.google.com/search?q=http://dx.doi.org/10.1200/jco.2020.39.28_suppl.18)
91. Vasudevan L, Bogus J, Kay H, Turner EL, Kwesigabo G, Brennan AT, et al. Using digital health to facilitate compliance with standardized pediatric cancer treatment guidelines in Tanzania: Protocol for an early-stage effectiveness-implementation hybrid study. BMC Cancer . 2020 Feb 7;20(1):105. Available from: [http://dx.doi.org/10.1186/s12885-020-6611-3](https://www.google.com/search?q=http://dx.doi.org/10.1186/s12885-020-6611-3)
92. Steinman L, Doescher M, Lehavot K, Ratzliff A, Katon W. Can mHealth and eHealth improve management of diabetes and hypertension in a hard-to-reach population?—lessons learned from a process evaluation of digital health to support a peer educator model in Cambodia using the RE-AIM framework. mHealth . 2020 Feb;6:5–5. Available from: <http://dx.doi.org/10.21037/mhealth.2020.02.01>
93. Pithara C, O’Hanlon P, Leahy D, O’Connor S, Giblin Y, Tully L, et al. Implementing a Digital Tool to Support Shared Care Planning in Community-Based Mental Health Services: Qualitative Evaluation. J Med Internet Res . 2020 Feb 12;22(2):e14868. Available from: <http://dx.doi.org/10.2196/14868>
94. Nolan E, Andersson N, Cockcroft A. Design and impact evaluation of a digital reproductive^6^ health program in Rwanda using a cluster randomized design: study protocol. BMC Public Health . 2020 Dec 3;20(1):1848. Available from: [http://dx.doi.org/10.1186/s12889-020-09746-7](https://www.google.com/search?q=http://dx.doi.org/10.1186/s12889-020-09746-7)
95. Minian N, Penner J, Voci S, Lecce J, Fikretoglu D, Selby P. Impact of a Web-Based Clinical Decision Support System to Assist Practitioners in Addressing Physical Activity and/or Healthy Eating for Smoking Cessation Treatment: Protocol for a Hybrid Type I Randomized Controlled Trial. JMIR Res Protoc . 2020 May 26;9(5):e19157. Available from: [http://dx.doi.org/10.2196/19157](https://www.google.com/search?q=http://dx.doi.org/10.2196/19157)
96. Meinert E, Milne-Ives M, Surodina S, Lam C. Agile Requirements Engineering and Software Planning for a Digital Health Platform to Engage the Effects of Isolation Caused by Social Distancing: Case Study. JMIR Public Health Surveill . 2020 May 14;6(2):e19297. Available from: <http://dx.doi.org/10.2196/19297>
97. Fuller TE, Pong J, Piniella N, Kamauf R, Morrison J, Bazar A, et al. Interactive Digital Health Tools to Engage Patients and Caregivers in Discharge Preparation: Implementation Study. J Med Internet Res . 2020 Apr 28;22(4):e15573. Available from: <http://dx.doi.org/10.2196/15573>
98. Freund T, Spies J, Gensichen J, Szecsenyi J, Stengel A, Ritter P, et al. Implementing internet- and tele-based interventions to prevent mental health disorders in farmers, foresters and gardeners (ImplementIT): study protocol for the multi-level evaluation of a nationwide project.^7^ BMC Psychiatry . 2020 Sep 29;20(1):466. Available from: <http://dx.doi.org/10.1186/s12888-020-02800-z>
99. Batsis JA, Naslund JA, Gill LE, Masutani RK, Agarwal N, Bartels SJ. Barriers and facilitators in implementing a pilot, pragmatic, telemedicine-delivered healthy lifestyle program for obesity management in a rural, academic obesity clinic. Implement Sci Commun . 2020 Dec 21;1(1):106. Available from: <http://dx.doi.org/10.1186/s43058-020-00075-9>
100. Bashi N, Fatehi F, Mosadeghi-Nik M, Askari MS, Karunanithi M. Digital health interventions for chronic diseases: a scoping review of evaluation frameworks. BMJ Health Care Inform . 2020 Feb;27(1):e100066. Available from: [http://dx.doi.org/10.1136/bmjhci-2019-100066](https://www.google.com/search?q=http://dx.doi.org/10.1136/bmjhci-2019-100066)
101. Van der Hout A, Heuvelman J, Brandenbarg D, Burger H, Oosterom N, Dijk van H, et al. E-health self-management applications targeting cancer survivors-who do we reach? Support Care Cancer . 2020 Feb;28(2):631–9. Available from: [http://dx.doi.org/10.1007/s00520-019-04813-1](https://www.google.com/search?q=http://dx.doi.org/10.1007/s00520-019-04813-1)
102. Rahal RM, Mercer J, Kuziemsky C, Yaya S. Primary Care Physicians’ Experience Using Advanced Electronic Medical Record Features to Support Chronic Disease Prevention and Management: Qualitative Study. JMIR Med Inform . 2019 Nov 29;7(4):e13318. Available from: [http://dx.doi.org/10.2196/13318](https://www.google.com/search?q=http://dx.doi.org/10.2196/13318)
103. Girman CJ, Ehrenstein V, Szatrowski TP, Michels K, Myles T, Lanciault C, et al. How do we assess when electronic health records or claims databases are fit for a specific research or regulatory purpose? Value Health. 2019 Apr;22(4):450–7.
104. Abdel-Wahab N, David L, Rizkalla K, Bruera E, Zimmermann C, Salvo N, et al. A comprehensive scoping review to identify standards for the development of health information resources on the internet. PLoS One . 2019 Aug 1;14(8):e0218342. Available from: <http://dx.doi.org/10.1371/journal.pone.0218342>
105. Blackman KC, Zoellner J, Berrey LM, Alexander R, Fanning J, Hill JL, et al. Assessing the internal and external validity of mobile health physical activity promotion interventions: a systematic literature review using the RE-AIM framework. J Med Internet Res . 2013 Oct 28;15(10):e224. Available from: <http://dx.doi.org/10.2196/jmir.2745>
106. Boatin AA, Cullinane F, Torloni MR, Betrán AP, Due-Christensen M, Grote V, et al. Wireless versus routine physiologic monitoring after cesarean delivery to reduce maternal morbidity^8^ and mortality in a resource-limited setting: protocol of type 2 hybrid effectiveness-implementation study. BMC Pregnancy Childbirth . 2021 Jan 13;21(1):45. Available from: <http://dx.doi.org/10.1186/s12884-021-03550-w>
107. Savira F, Mewton L, Said D, Bhullar N, Chow C, Redfern J. Virtual Care Initiatives for Older Adults in Australia: Scoping Review. J Med Internet Res . 2023 Mar 29;25:e38081. Available from: [http://dx.doi.org/10.2196/38081](https://www.google.com/search?q=http://dx.doi.org/10.2196/38081)
108. Fernando J, Mastellos N, Majeed A, Tully MP. Using Theories, Models, and Frameworks to Inform Implementation Cycles of Computerized Clinical Decision Support Systems in Tertiary Health Care Settings: Scoping Review. JMIR Med Inform . 2023 Feb 27;11:e45163. Available from: [http://dx.doi.org/10.2196/45163](https://www.google.com/search?q=http://dx.doi.org/10.2196/45163)
109. Reid JL, Chrysanthopoulou SA, Sharma S, Lam CT, Rosen RK, Rositch AF. Using the Consolidated Framework for Implementation Research to Inform the Design of the Mobile Inspección Visual con Ácido Acético System: Mixed Methods Case Study. JMIR Form Res . 2022 Aug 16;6(8):e32577. Available from: [http://dx.doi.org/10.2196/32577](https://www.google.com/search?q=http://dx.doi.org/10.2196/32577)
110. Fan S, Leuba SI, Yao H, Zheng H, Xu M, Zhao Y, et al. Uptake of an App-Based Case Management Service for HIV-Positive Men Who Have Sex With Men in China: Process Evaluation Study. JMIR Form Res . 2023 Feb 22;7:e40176. Available from: [http://dx.doi.org/10.2196/40176](https://www.google.com/search?q=http://dx.doi.org/10.2196/40176)
111. Tiwari S, Sharma P, Khatri A, Shrestha S, Yadav R, Maru D, et al. A Type II hybrid effectiveness-implementation study of an integrated CHW intervention to address maternal healthcare in rural Nepal. PLOS Glob Public Health . 2023 Feb 22;3(2):e0001512. Available from: [http://dx.doi.org/10.1371/journal.pgph.0001512](https://www.google.com/search?q=http://dx.doi.org/10.1371/journal.pgph.0001512)
112. Tighe J, Shand F, McKay K, Bryant Z, Larsen ME. Toward a Digital Platform for the Self-Management of Noncommunicable Disease: Systematic Review of Platform-Like Interventions. J Med Internet Res . 2020 Jun 25;22(6):e16774. Available from: [http://dx.doi.org/10.2196/16774](https://www.google.com/search?q=http://dx.doi.org/10.2196/16774)
113. Du G, Theodore RM, Johnson V, Ballard KJ. “They Can’t Believe They’re a Tiger”: Insights from pediatric speech-language pathologist mobile app users and app designers. International Journal of Language & Communication Disorders . 2023 Mar;58(2):692–708. Available from: [http://dx.doi.org/10.1111/1460-6984.12898](https://www.google.com/search?q=http://dx.doi.org/10.1111/1460-6984.12898)
114. Hussain MS, Silvera-Tawil D, Farr-Wharton G. Technology assessment framework for precision health applications. Int J Technol Assess Health Care . 2021;37(1):e67. Available from: [http://dx.doi.org/10.1017/s0266462321000350](https://www.google.com/search?q=http://dx.doi.org/10.1017/s0266462321000350)
115. Brown LL, Jones KT, Mathias R, Lewis L, Brown EA, Jones VC, et al. A Suicide Prevention Intervention for Emerging Adult Sexual and Gender Minority Groups: Protocol for a Pilot Hybrid Effectiveness Randomized Controlled Trial. JMIR Res Protoc . 2023 Jul 20;12:e48177. Available from: [http://dx.doi.org/10.2196/48177](https://www.google.com/search?q=http://dx.doi.org/10.2196/48177)
116. Domin A, Spruijt-Metz D, Theng Y-L, Sandeep M, Müller-Riemenschneider F. Smartphone-Based Interventions for Physical Activity Promotion: Scoping Review of the Evidence Over the Last 10 Years. JMIR Mhealth Uhealth . 2021 Jul 20;9(7):e24308. Available from: [http://dx.doi.org/10.2196/24308](https://www.google.com/search?q=http://dx.doi.org/10.2196/24308)
117. Hensher M, Cooper C, Ryder K, Scuffham P, Scott IA. Scoping review: Development and assessment of evaluation frameworks of mobile health apps for recommendations to consumers. J Am Med Inform Assoc . 2021 Oct 12;28(11):2496–509. Available from: [http://dx.doi.org/10.1093/jamia/ocab041](https://www.google.com/search?q=http://dx.doi.org/10.1093/jamia/ocab041)
118. Schueller SM, Torous J, Beidas RS. Scaling evidence-based treatments through digital mental health. Am Psychol . 2020 Nov;75(8):1093–104. Available from: [http://dx.doi.org/10.1037/amp0000654](https://www.google.com/search?q=http://dx.doi.org/10.1037/amp0000654)
119. Suleman S, Sidat M, Moon T, Matavele R, Sixpence A, Van Der Kop M, et al. Scale-Up Study Protocol of the Implementation of a Mobile Health SBIRT Approach for Alcohol Use Reduction in Mozambique. Psychiatr Serv . 2021 Jun;72(6):721–4. Available from: [http://dx.doi.org/10.1176/appi.ps.202000086](https://www.google.com/search?q=http://dx.doi.org/10.1176/appi.ps.202000086)
120. Lisowska A, Wilk S, Peleg M. SATO (IDEAS expAnded wiTh BCIO): Workflow for designers of patient-centered mobile health behaviour change intervention applications. J Biomed Inform . 2023 Feb;138:104276. Available from: [http://dx.doi.org/10.1016/j.jbi.2022.104276](https://www.google.com/search?q=http://dx.doi.org/10.1016/j.jbi.2022.104276)
121. Lessard L, Sauvé J-S, Michalowski W, Kuziemsky C, Sargious P, Fung D, et al. Remote Follow-up of Self-isolating Patients With COVID-19 Using a Patient Portal: Protocol for a Mixed Methods Pilot Study (Opal-COVID Study). JMIR Res Protoc . 2022 Apr 1;11(4):e35760. Available from: [http://dx.doi.org/10.2196/35760](https://www.google.com/search?q=http://dx.doi.org/10.2196/35760)
122. Tran B, Smith C, Mutseta M, Chikwava S, Muchedzi A, Shambira G, et al. REDCap mobile data collection: Using implementation science to explore the potential and pitfalls of a digital health tool in routine voluntary medical male circumcision outreach settings in Zimbabwe. Digit Health . 2022 Jan;8:20552076221112163. Available from: [http://dx.doi.org/10.1177/20552076221112163](https://www.google.com/search?q=http://dx.doi.org/10.1177/20552076221112163)
123. Berretta E, Bonfanti A, Cavallo F, Mauri G, Pravettoni G. A RE-AIM Analysis of a Mental Health App for Undergraduate and Medical Students during the COVID-19 Pandemic: A Retrospective Cross-Sectional Study. Int J Environ Res Public Health . 2023 Jun 28;20(13):6266. Available from: [http://dx.doi.org/10.3390/ijerph20136266](https://www.google.com/search?q=http://dx.doi.org/10.3390/ijerph20136266)
124. Ferrari M, Seto E, Archie S, Fu B, Chakraborty S, Foussias G, et al. A Rapid-Learning Health System to Support Implementation of Early Intervention Services for Psychosis in Quebec, Canada: Protocol. JMIR Res Protoc . 2022 Jul 27;11(7):e37346. Available from: <http://dx.doi.org/10.2196/37346>
125. Hayman M, Brown W, Ferrar K, Davis S, Mizdrak A. Quality, features, and presence of behavior change techniques in mobile apps designed to improve physical activity in pregnant women: Systematic search and content analysis. JMIR Mhealth Uhealth . 2021 May 17;9(5):e23649. Available from: <http://dx.doi.org/10.2196/23649>
126. Laws R, Campbell K, Hesketh K, Zheng M, U H, Litterbach E, et al. Protocol for an Effectiveness-Implementation Hybrid Trial to Evaluate Scale up of an Evidence-Based Intervention Addressing Lifestyle Behaviours From the Start of Life: INFANT. Front Endocrinol (Lausanne) . 2021 Aug 11;12:717468. Available from: [http://dx.doi.org/10.3389/fendo.2021.717468](https://www.google.com/search?q=http://dx.doi.org/10.3389/fendo.2021.717468)
127. Scherer^9^ J, Youssef Y, Dittrich F, Albrecht UV, Tsitsilonis S, Jung J, et al. Proposal of a New Rating Concept for Digital Health Applications in Orthopedics and Traumatology. Int J Environ Res Public Health . 2022 Nov 13;19(22):14952. Available from: [http://dx.doi.org/10.3390/ijerph192214952](https://www.google.com/search?q=http://dx.doi.org/10.3390/ijerph192214952)
128. Wang J, Xue H, Pan J, Zhong X, Xie X. Promoting healthy lifestyle in Chinese college students: evaluation of a social media-based intervention applying the RE-AIM framework. Eur J Clin Nutr . 2021 Feb;75(2):375–82. Available from: [http://dx.doi.org/10.1038/s41430-020-0643-2](https://www.google.com/search?q=http://dx.doi.org/10.1038/s41430-020-0643-2)
129. Fortuna K, Ferron J, Ferrante D, Fine E, Scherer E, Myers A. Patient-centric approach to utilizing visual misperception as a marker for neuropsychiatric disease. Early Interv Psychiatry. 2023;17 Suppl 1:135–135.
130. White K, Taylor J, Turner K, Østbye T, Lipkus I, Jones L, et al. Organizational trust, usability, and inclusivity are key implementation facilitators for a proposed assets-based mobile health intervention. Transl Behav Med . 2023 Feb 14;13(2):110–9. Available from: [http://dx.doi.org/10.1093/tbm/ibac108](https://www.google.com/search?q=http://dx.doi.org/10.1093/tbm/ibac108)
131. Bradford N, Chamberlain C, Chan R, Taylor J, Williams L, Davies M, et al. Optimising symptom management in children with cancer using a novel mobile phone application: protocol for a controlled hybrid effectiveness implementation trial (RESPONSE). BMC Health Serv Res . 2021 Oct 19;21(1):1091. Available from: [http://dx.doi.org/10.1186/s12913-021-06943-x](https://www.google.com/search?q=http://dx.doi.org/10.1186/s12913-021-06943-x)
132. Glasgow L, Powell A, Taylor L, Thompson A, Nelson T, Rabin C, et al. The NUDGE trial pragmatic trial to enhance cardiovascular medication adherence: study protocol for a randomized controlled trial. Trials . 2021 Aug 9;22(1):523. Available from: [http://dx.doi.org/10.1186/s13063-021-05453-9](https://www.google.com/search?q=http://dx.doi.org/10.1186/s13063-021-05453-9)
133. Lu R, Yan H, Zhang J, Xie H, Wang S, Li R, et al. Mom’s Good Mood: screening and management of perinatal depression within primary healthcare system in China—protocol for an effectiveness-implementation design study. BMJ Open . 2023 May 9;13(5):e063593. Available from: [http://dx.doi.org/10.1136/bmjopen-2022-063593](https://www.google.com/search?q=http://dx.doi.org/10.1136/bmjopen-2022-063593)
134. Woulfe F, Fadahunsi KP, O’Grady M, Chirambo GB, Mawkin M, Majeed A, et al. Modification and Validation of an mHealth App Quality Assessment Methodology for International Use: Cross-sectional and eDelphi Studies. JMIR Form Res . 2022 Aug 19;6(8):e36912. Available from: [http://dx.doi.org/10.2196/36912](https://www.google.com/search?q=http://dx.doi.org/10.2196/36912)
135. Charness N, Boot W, Czaja S, Rogers W. Mobile Monitoring and Intervention (MMI) Technology for Adaptive Aging. Washington, D.C.: National Academies Press; 2020 Nov 5.
136. Azad-Khaneghah P, Neubauer N, Miguel Cruz A, Liu L. Mobile health app usability and quality rating scales: a systematic review. Disabil Rehabil Assist Technol . 2021 Oct;16(7):712–21. Available from: [http://dx.doi.org/10.1080/17483107.2019.1701103](https://www.google.com/search?q=http://dx.doi.org/10.1080/17483107.2019.1701103)
137. Gomm SIM, Ebner FK, Lukac S, El Taie Z, Janni W, Schmidt-Straßburger U, et al. Mobile Applications Available in Germany Supporting Breast Cancer Patients During Treatment and Aftercare: a Systematic Review. Geburtshilfe Frauenheilkd . 2022 Sep 13;82(9):941–54. Available from: [http://dx.doi.org/10.1055/a-1909-8736](https://www.google.com/search?q=http://dx.doi.org/10.1055/a-1909-8736)
138. Li H, Zhang Q, Zhou H, Li H, Xiao M, Xiao S, et al. Mobile App-Based Intervention for Pregnant Women With Stress Urinary Incontinence: Protocol for a Hybrid Effectiveness-Implementation Trial. JMIR Res Protoc . 2021 Nov 23;10(11):e22771. Available from: [http://dx.doi.org/10.2196/22771](https://www.google.com/search?q=http://dx.doi.org/10.2196/22771)
139. Young M, Thomas A, Austin Z, Lubarsky S, Raîche I, Dojeiji S, et al. A mobile app to capture EPA assessment data: Utilizing the consolidated framework for implementation research to identify enablers and barriers to engagement. Adv Health Sci Educ Theory Pract . 2021 May;26(2):681–700. Available from: [http://dx.doi.org/10.1007/s10459-020-10010-6](https://www.google.com/search?q=http://dx.doi.org/10.1007/s10459-020-10010-6)
140. Chang W, Psaros C, Musiimenta A, Tsai AC, Burns BFO, Haberer JE. Mixed Methods, Implementation Science Evaluation of a Community Health Worker Strategy for HIV Service Engagement in Uganda. J Acquir Immune Defic Syndr . 2023^10^ Nov 1;94(3):262–70. Available from: [http://dx.doi.org/10.1097/qai.0000000000003220](https://www.google.com/search?q=http://dx.doi.org/10.1097/qai.0000000000003220)
141. Godinho MA, Choy WL, Medagedara SC, Karunanithi M. mHealth for Integrated People-Centred Health Services in the Western Pacific: A Systematic Review. Int J Med Inform . 2021 Jan;145:104259. Available from: [http://dx.doi.org/10.1016/j.ijmedinf.2020.104259](https://www.google.com/search?q=http://dx.doi.org/10.1016/j.ijmedinf.2020.104259)
142. Gladman T, Tylee G, Gallagher S, Mair J, Rennie S, Grainger R. Measuring the Quality of Clinical Skills Mobile Apps for Student Learning: Systematic Search, Analysis, and Comparison of Two Measurement Scales. JMIR Mhealth Uhealth . 2021 Feb 25;9(2):e25377. Available from: <http://dx.doi.org/10.2196/25377>
143. Pearson SD, Singh P, Beaudoin F, Campbell J, Schapiro L, Emond SK, et al. Institute for Clinical and Economic Review - Peterson Health Technology Institute value assessment framework for digital health technologies. J Comp Eff Res . 2023 Dec;12(12):e230154. Available from: [http://dx.doi.org/10.57264/cer-2023-0154](https://www.google.com/search?q=http://dx.doi.org/10.57264/cer-2023-0154)
144. Jaffar F, Omar M, Ramli N. Improving Pelvic Floor Muscle Training Adherence Among Pregnant Women: Validation Study. JMIR Form Res . 2022 Sep 26;6(9):e30989. Available from: <http://dx.doi.org/10.2196/30989>
145. Siebenhüner AR, Batliner M, Spörri B, Krüger S, Huber F, Stanga Z, et al. Improvements in Health Might Contradict Adherence to Mobile Health Interventions: Findings from a Self-Care Cancer App Study. J Altern Complement Med . 2021 Mar;27(3):258–64. Available from: [http://dx.doi.org/10.1089/acm.2020.0111](https://www.google.com/search?q=http://dx.doi.org/10.1089/acm.2020.0111)
146. Ye J, Wang T, Yang Y, Yin J, Fan X, Zhou M, et al. Implications for implementation and adoption of telehealth in developing countries: a systematic review of China’s practices and experiences. NPJ Digit Med . 2023 Sep 22;6(1):172. Available from: [http://dx.doi.org/10.1038/s41746-023-00908-6](https://www.google.com/search?q=http://dx.doi.org/10.1038/s41746-023-00908-6)
147. Franzoi M, Jardim D, Thompson C, Symecko H, Hilsenbeck S, Osborne C, et al. Implementing a PROACTive Care Pathway to Empower and Support Survivors of Breast Cancer. JCO Oncol Pract . 2023 Apr;19(4):274–80. Available from: [http://dx.doi.org/10.1200/op.23.00016](https://www.google.com/search?q=http://dx.doi.org/10.1200/op.23.00016)
148. Anderson J, Hoover J, Nez Henderson P, Powers M, Beene-Dakota S, Christie C, et al. Implementing a Community-Led Arsenic Mitigation Intervention for Private Well Users in American Indian Communities: A Qualitative Evaluation of the Strong Heart Water Study Program. Int J Environ Res Public Health . 2023 Jan 31;20(3):2681. Available from: [http://dx.doi.org/10.3390/ijerph20032681](https://www.google.com/search?q=http://dx.doi.org/10.3390/ijerph20032681)
149. Cohn EA, Fletcher J, Crusoe K, Moore R, Fernandez MI. An Implementation Strategy to Expand Mobile Health Use in HIV Care Settings: Rapid Evaluation Study Using the Consolidated Framework for Implementation Research. JMIR Form Res . 2021 Jan 29;5(1):e19163. Available from: <http://dx.doi.org/10.2196/19163>
150. Nelson K, Helfrich C, Ralston J. Implementation of Technology-Delivered Diabetes Self-care Interventions in Clinical Care: a Narrative Review. Curr Diab Rep . 2021 Jan;21(1):1. Available from: [http://dx.doi.org/10.1007/s11892-020-01356-2](https://www.google.com/search?q=http://dx.doi.org/10.1007/s11892-020-01356-2)
151. Philpot LM, Siddall P, Lieberman D, Gauntlett-Gilbert J, Campkin S, Duncombe D, et al. Implementation of eLearning solutions for patients with chronic pain conditions. Digit Health . 2023 Jan;9:20552076231216404. Available from: [http://dx.doi.org/10.1177/20552076231216404](https://www.google.com/search?q=http://dx.doi.org/10.1177/20552076231216404)
152. Ellis LA, Meulenbroeks I, Churruca K, Pomare C, Hatem S, Harrison R, et al. Implementation of e-mental health for depression and anxiety: A critical scoping review. J Community Psychol . 2020 Oct;48(7):2165–86. Available from: [http://dx.doi.org/10.1002/jcop.22309](https://www.google.com/search?q=http://dx.doi.org/10.1002/jcop.22309)
153. Cho J, Thomas ML, Peterson RA, Fischer LE, Smith JL, Dietz LR, et al. Implementation of a Scalable Online Weight Management Programme in Clinical Settings: Protocol for the PROPS 2.0 Programme (Partnerships for Reducing Overweight and Obesity with Patient-Centered Strategies 2.0). BMJ Open . 2023 Nov 22;13(11):e077520. Available from: [http://dx.doi.org/10.1136/bmjopen-2023-077520](https://www.google.com/search?q=http://dx.doi.org/10.1136/bmjopen-2023-077520)
154. Smith JL, Mohan S, Gordon EJ, Byrd JB, Dadhania D, Doshi MD, et al. Implementation of a culturally competent APOL1 genetic testing programme into living donor evaluation: a two-site, non-randomised, pre–post trial design. BMJ Open . 2023 Jul 25;13(7):e067657. Available from: [http://dx.doi.org/10.1136/bmjopen-2022-067657](https://www.google.com/search?q=http://dx.doi.org/10.1136/bmjopen-2022-067657)
155. Badawy S, Thompson A, Liem R. Implementation And Preliminary Effectiveness Of Mhealth Apps For Improving Sickle Cell Disease Care During Covid-19: A Mixed-Methods Evaluation. Blood . 2021 Nov 23;138(Supplement 1). Available from: [http://dx.doi.org/10.1182/blood-2021-146411](https://www.google.com/search?q=http://dx.doi.org/10.1182/blood-2021-146411)
156. Ng TP, Nyunt MSZ, Feng L, Lim WS, Chan G, Lee TS, et al. Implementation and effectiveness of a multi-domain program for older adults at risk of cognitive impairment at neighborhood senior centres. Sci Rep . 2021 Feb 17;11(1):4046. Available from: [http://dx.doi.org/10.1038/s41598-021-83408-5](https://www.google.com/search?q=http://dx.doi.org/10.1038/s41598-021-83408-5)
157. Badawy SM, Shah N, Owoyemi P, Thompson AA, Liem RI. Impact of the COVID-19 Pandemic on the Implementation of Mobile Health to Improve the Uptake of Hydroxyurea in Patients With Sickle Cell Disease: Mixed Methods Study. JMIR Form Res . 2022 Nov 9;6(11):e41415. Available from: [http://dx.doi.org/10.2196/41415](https://www.google.com/search?q=http://dx.doi.org/10.2196/41415)
158. Edwards JK, Asiimwe S, Geng E, Reynolds SJ, Bwana MB, Kabami J, et al. Impact of community health worker intervention on PrEP knowledge and use in Rakai, Uganda: A mixed methods, implementation science evaluation. Int J STD AIDS . 2022 Sep;33(10):869–79. Available from: [http://dx.doi.org/10.1177/09564624221121208](https://www.google.com/search?q=http://dx.doi.org/10.1177/09564624221121208)
159. Alnooh G, Fournier A, Estabrooks P. Identification of the Most Suitable Mobile Apps to Support Dietary Approaches to Stop Hypertension (DASH) Diet Self-Management: Systematic Search of App Stores and Content Analysis. Nutrients . 2023 Aug 2;15(15):3476. Available from: [http://dx.doi.org/10.3390/nu15153476](https://www.google.com/search?q=http://dx.doi.org/10.3390/nu15153476)
160. Scott IA, Scuffham P, Gupta D, Harch TM, Borchi J, Richards B. Going digital: a narrative overview of the effects, quality and utility of mobile apps in chronic disease self-management. Aust Health Rev . 2020 Feb;44(1):62–82. Available from: [http://dx.doi.org/10.1071/ah18064](https://www.google.com/search?q=http://dx.doi.org/10.1071/ah18064)
161. Tan SS, Woulfe F, Mawkin M, Chirambo GB, O’Donoghue J, Smith S, et al. Framework to assess the quality of mHealth apps: a mixed-method international case study protocol. BMJ Open . 2022 Sep 19;12(9):e062909. Available from: [http://dx.doi.org/10.1136/bmjopen-2022-062909](https://www.google.com/search?q=http://dx.doi.org/10.1136/bmjopen-2022-062909)
162. Kleckner IR, Feldman MJ, Goodwin MS, Hughes BL, Joiner SA, Lord H, et al. Framework for selecting and benchmarking mobile devices in psychophysiological research. Behav Res Methods . 2021 Feb;53(2):518–35. Available from: [http://dx.doi.org/10.3758/s13428-020-01438-9](https://www.google.com/search?q=http://dx.doi.org/10.3758/s13428-020-01438-9)
163. Sit HFW, Xia J, Leung D, Hung S-F, Sklar M, Cuijpers P, et al. A Feasibility Study of the WHO Digital Mental Health Intervention Step-by-Step to Address Depression Among Chinese Young Adults. Front Psychiatry . 2022 Jan 13;12:812667. Available from: [http://dx.doi.org/10.3389/fpsyt.2021.812667](https://www.google.com/search?q=http://dx.doi.org/10.3389/fpsyt.2021.812667)
164. Tsangaris E, Wong RKN, Oldfield LE, Husain A, Musa Z, Martins D, et al. Feasibility of implementing patient-reported outcome measures into routine breast cancer care delivery using a novel collection and reporting platform. JAMIA Open . 2024 Jan;7(1):ooad108. Available from: [http://dx.doi.org/10.1093/jamiaopen/ooad108](https://www.google.com/search?q=http://dx.doi.org/10.1093/jamiaopen/ooad108)
165. Ming D, Miller EG, Perrin JM, Van Cleave J, Kuhlthau K, Gotlieb E, et al. Feasibility of Implementation of a Mobile Digital Personal Health Record to Coordinate Care for Children and Youth With Special Health Care Needs in Primary Care: Protocol for a Mixed Methods Study. JMIR Res Protoc . 2023 Jun 15;12:e46847. Available from: [http://dx.doi.org/10.2196/46847](https://www.google.com/search?q=http://dx.doi.org/10.2196/46847)
166. Cordoba K, Rosales S, Wilson P, Dowshen N, Fernández M. Examining the Information Systems Success (ISS) of a mobile sexual health app (MyPEEPS Mobile) from the perspective of very young men who have sex with men (YMSM). Int J Med Inform . 2021 Oct;154:104529. Available from: [http://dx.doi.org/10.1016/j.ijmedinf.2021.104529](https://www.google.com/search?q=http://dx.doi.org/10.1016/j.ijmedinf.2021.104529)
167. ElJoueidi S, Tilahun B, Smillie K, Murray M, Bardosh K, Fitzgerald M, et al. Evaluation of the implementation process of the mobile health platform ‘WelTel’ in six sites in East Africa and Canada using the modified consolidated framework for implementation research (mCFIR). BMC Med Inform Decis Mak . 2021 Oct 18;21(1):284. Available from: <http://dx.doi.org/10.1186/s12911-021-01644-1>
168. Sharma S, Gergen Barnett K, Maypole JJ, Grochow Mishuris R. Evaluation of mHealth Apps for Diverse, Low-Income Patient Populations: Framework Development and Application Study. JMIR Form Res . 2022 Feb 11;6(2):e29922. Available from: [http://dx.doi.org/10.2196/29922](https://www.google.com/search?q=http://dx.doi.org/10.2196/29922)
169. Raeesi A, Khajouei R, Ahmadian L. Evaluation of HIV/AIDS-related mobile health applications content using an evidence-based content rating tool. BMC Med Inform Decis Mak . 2021 May 1;21(1):135. Available from: [http://dx.doi.org/10.1186/s12911-021-01498-7](https://www.google.com/search?q=http://dx.doi.org/10.1186/s12911-021-01498-7)
170. Koziol-McLain J, Vandal AC, Wilson D, Nada-Raja S, Dobbs T, Borman B, et al. Evaluation of a Healthy Relationship Smartphone App With Indigenous Young People: Protocol for a Co-designed Stepped Wedge Randomized Trial. JMIR Res Protoc . 2021 Mar 19;10(3):e24792. Available from: [http://dx.doi.org/10.2196/24792](https://www.google.com/search?q=http://dx.doi.org/10.2196/24792)
171. Niemann CE, Grothe C, Meyer-Massetti C, Hersberger KE, Arnet I. Evaluation criteria for health apps supporting medication adherence in early-stage technology development - a scoping review. GMS Med Inform Biom Epidemiol . 2023 Apr 19;19:Doc06. Available from: [http://dx.doi.org/10.3205/mibe000244](https://www.google.com/search?q=http://dx.doi.org/10.3205/mibe000244)
172. Ardito C, Cianciotta M, Corcione S, De Nunzio A, Di Palma G, Diroma V, et al. Evaluating Barriers and Facilitators to the Uptake of mHealth Apps in Cancer Care Using the Consolidated Framework for Implementation Research: Scoping Literature Review. J Med Internet Res . 2023 May 9;25:e42092. Available from: <http://dx.doi.org/10.2196/42092>
173. Carter AJE, Rehman N, Poss JW, Thompson G, Martin DE, Archambault PM, et al. Essential Elements to Implementing a Paramedic Palliative Model of Care: An Application of the Consolidated Framework for Implementation Research. J Palliat Med . 2022 Jun;25(6):929–43. Available from: [http://dx.doi.org/10.1089/jpm.2021.0459](https://www.google.com/search?q=http://dx.doi.org/10.1089/jpm.2021.0459)
174. Franzmair K, Höller K, Ambros K, Zacharasiewicz A. Effective German and English Language mHealth Apps for Self-management of Bronchial Asthma in Children and Adolescents: Comparison Study. JMIR Pediatr Parent . 2021 Aug 25;4(3):e24907. Available from: <http://dx.doi.org/10.2196/24907>
175. Qin ZZ, Bastard M, Van Cutsem G, Mpunga J, Makame M, Mapanga D, et al. Early user experience and lessons learned using ultra-portable digital X-ray with computer-aided detection (DXR-CAD) products: A qualitative study from the perspective of healthcare providers. PLoS One . 2023 Jan 27;18(1):e0277843. Available from: [http://dx.doi.org/10.1371/journal.pone.0277843](https://www.google.com/search?q=http://dx.doi.org/10.1371/journal.pone.0277843)
176. Khan WU, Seto E. A “Do No Harm” Novel Safety Checklist and Research Approach to Determine Whether to Launch an Artificial Intelligence–Based Medical Technology: Introducing the Biological-Psychological, Economic, and Social (BPES) Framework. J Med Internet Res . 2023 Apr 5;25:e43386. Available from: [http://dx.doi.org/10.2196/43386](https://www.google.com/search?q=http://dx.doi.org/10.2196/43386)
177. Melvin SC, Wiggins S, Burse N, Thompson E, Mongraw-Chaffin M. Dissemination and Implementation of a Google Apple Exposure Notification System for COVID-19 Risk Mitigation at a National Public University: Protocol for a Pilot Evaluation Study in a Real-World Setting. JMIR Res Protoc . 2022 Apr 8;11(4):e32567. Available from: <http://dx.doi.org/10.2196/32567>
178. Liem A, Wang C, Lau JTF, van Griensven F. A digital mental health intervention to reduce depressive symptoms among overseas Filipino workers: protocol for a pilot hybrid type 1 effectiveness-implementation randomized controlled trial. Implement Sci Commun . 2020 Dec 15;1(1):103. Available from: [http://dx.doi.org/10.1186/s43058-020-00072-y](https://www.google.com/search?q=http://dx.doi.org/10.1186/s43058-020-00072-y)
179. Wagner R, Brownell C, Hinton C, Rintala F, Carter P, Graham C, et al. Development of an App for Tracking Family Engagement With Early Intervention Services: Focus Groups and Pilot Evaluation Study. JMIR Form Res . 2023 May 12;7:e45957. Available from: [http://dx.doi.org/10.2196/45957](https://www.google.com/search?q=http://dx.doi.org/10.2196/45957)
180. Ribaut J, DeVito Dabbs A, Dobbels F, Teynor A, Mess EV, Hoffmann T, et al. Developing a Comprehensive List of Criteria to Evaluate the Characteristics and Quality of eHealth Smartphone Apps: Systematic Review. JMIR Mhealth Uhealth . 2024 Jan 15;12:e48625. Available from: [http://dx.doi.org/10.2196/48625](https://www.google.com/search?q=http://dx.doi.org/10.2196/48625)
181. Alon D, Avram A, Gordon M, Levkovich I, Balicer R, Shadmi E. Current challenges for evaluating mobile health applications. J Am Med Inform Assoc . 2023 Feb 14;30(3):593–600. Available from: [http://dx.doi.org/10.1093/jamia/ocac244](https://www.google.com/search?q=http://dx.doi.org/10.1093/jamia/ocac244)
182. Sujarwoto S, Tampubolon G, Prima A. COVID-19 Mobile Health Apps: An Overview of Mobile Applications in Indonesia. Front Public Health . 2022 May 25;10:879695. Available from: [http://dx.doi.org/10.3389/fpubh.2022.879695](https://www.google.com/search?q=http://dx.doi.org/10.3389/fpubh.2022.879695)
183. Muñoz-Mancisidor A, Pérez-Ríos M, Ruano-Ravina A, Varela-Lema L. Content, Behavior Change Techniques, and Quality of Pregnancy Apps in Spain: Systematic Search on App Stores. JMIR Mhealth Uhealth . 2021 Jul 19;9(7):e27995. Available from: [http://dx.doi.org/10.2196/27995](https://www.google.com/search?q=http://dx.doi.org/10.2196/27995)
184. Lee M, Partridge SR, Torquati L, Allman-Farinelli M, Neubeck L. A content and quality analysis of free, popular mHealth apps supporting ‘plant-based’ diets. PLOS Digit Health . 2023 Oct 5;2(10):e0000360. Available from: [http://dx.doi.org/10.1371/journal.pdig.0000360](https://www.google.com/search?q=http://dx.doi.org/10.1371/journal.pdig.0000360)
185. McAleese E, McAleese D, Linardakis M, Papadaki A. Quality and Presence of Behaviour Change Techniques in Mobile Apps for the Mediterranean Diet: A Content Analysis of Android Google Play and Apple App Store Apps. Nutrients. 2022;14(6):1290. Published 2022 Mar 18. doi:10.3390/nu14061290
186. O’Reilly S, Laws R, Hearn L, Harrison C, Devenish G, Brown J, et al. A Complex mHealth Coaching Intervention to Prevent Overweight, Obesity, and Diabetes in High-Risk Women in Antenatal Care: Protocol for a Hybrid Type 2 Effectiveness-Implementation Study. JMIR Res Protoc . 2023 Nov 30;12:e51431. Available from: [http://dx.doi.org/10.2196/51431](https://www.google.com/search?q=http://dx.doi.org/10.2196/51431)
187. Hirst J, Sharma M, De Costa A, Chand K, Gupta A, Mohanty A, et al. A community-based intervention to improve screening, referral and follow-up of non-communicable diseases and anaemia amongst pregnant and postpartum women in rural India: study protocol for a cluster randomised trial. Trials . 2023 Aug 8;24(1):510. Available from: [http://dx.doi.org/10.1186/s13063-023-07510-x](https://www.google.com/search?q=http://dx.doi.org/10.1186/s13063-023-07510-x)
188. Ageberg E, Fältström A, Bunke S, Lucander K, Eliasson K, Dahlberg LE, et al. Co-creating holistic injury prevention training for youth handball: Development of an intervention targeting end-users at the individual, team, and organizational levels. Inj Epidemiol . 2024 Jan 10;11(1):2. Available from: [http://dx.doi.org/10.1186/s40621-023-00498-0](https://www.google.com/search?q=http://dx.doi.org/10.1186/s40621-023-00498-0)
189. Matsuoka H, Okuyama H, Sakashita A, Nakashima K, Nakagawa T, Otsuka M, et al. Barriers and facilitators to implementing geriatric assessment in daily oncology practice in Japan: A qualitative study using an implementation framework. J Geriatr Oncol . 2024 Jan;15(1):101625. Available from: [http://dx.doi.org/10.1016/j.jgo.2023.101625](https://www.google.com/search?q=http://dx.doi.org/10.1016/j.jgo.2023.101625)
190. Meijer E, Wiers RW, Boendermaker WJ, Van Laarhoven AIM, Smit F. “At least someone thinks I’m doing well”: a real-world evaluation of the quit-smoking app StopCoach for lower socio-economic status smokers. Subst Abuse Treat Prev Policy . 2021 Nov 22;16(1):83. Available from: [http://dx.doi.org/10.1186/s13011-021-00418-z](https://www.google.com/search?q=http://dx.doi.org/10.1186/s13011-021-00418-z)
191. Stecher C, Schlichtiger J, Utesch T, Mess F. Assessing the Pragmatic Nature of Mobile Health Interventions Promoting Physical Activity: Systematic Review and Meta-analysis. JMIR Mhealth Uhealth . 2023 Apr 28;11:e43162. Available from: [http://dx.doi.org/10.2196/43162](https://www.google.com/search?q=http://dx.doi.org/10.2196/43162)
192. de la Vega R, Rosner B, Zempsky WT, Palermo TM. Assessing Digital Health Implementation for a Pediatric Chronic Pain Intervention: Comparing the RE-AIM and BIT Frameworks Against Real-World Trial Data and Recommendations for Future Studies. J Med Internet Res . 2020 Jul 8;22(7):e19898. Available from: <http://dx.doi.org/10.2196/19898>
193. Grau-Corral I, Pantoja PE, Grajales Iii FJ, Kostov B, Aragunde V, Puig-Soler M, et al. Assessing Apps for Health Care Workers Using the ISYScore-Pro Scale: Development and Validation Study. JMIR Mhealth Uhealth . 2021 Jul 21;9(7):e17660. Available from: [http://dx.doi.org/10.2196/17660](https://www.google.com/search?q=http://dx.doi.org/10.2196/17660)
194. Fortuna KL, Myers AL, Walsh D, Walker R, Moino K, Ferron JC, et al. “As soon as I start trusting human beings, they disappoint me, and now I am going to get on an app that someone could hack. I really do not want to take that chance”: barriers and facilitators to digital peer support implementation into community mental health centers. Front Digit Health . 2023 Mar 23;5:1130095. Available from: [http://dx.doi.org/10.3389/fdgth.2023.1130095](https://www.google.com/search?q=http://dx.doi.org/10.3389/fdgth.2023.1130095)
195. Rasmussen CDN, Østerås H, Kvamme T, Nøst TH, Nicholl BI, Hartvigsen J, et al. App-Delivered Self-Management Intervention Trial selfBACK for People With Low Back Pain: Protocol for Implementation and Process Evaluation. JMIR Res Protoc . 2020 Sep 22;9(9):e20308. Available from: [http://dx.doi.org/10.2196/20308](https://www.google.com/search?q=http://dx.doi.org/10.2196/20308)
196. Baloh J, Borodovsky JT, Huhn AS, Dunn KE. Al-Anon Intensive Referral (AIR): A qualitative formative evaluation for implementation. J Subst Abuse Treat . 2022 Feb;133:108520. Available from: [http://dx.doi.org/10.1016/j.jsat.2021.108520](https://www.google.com/search?q=http://dx.doi.org/10.1016/j.jsat.2021.108520)
197. Quimby K, Gibson A, Buckley B, Haynes E, Unwaha T, Alleyne G, et al. Adaptation of a community-based type-2 diabetes mellitus remission intervention during COVID-19: empowering persons living with diabetes to take control. Implement Sci Commun . 2022 Feb 16;3(1):14. Available from: [http://dx.doi.org/10.1186/s43058-022-00255-9](https://www.google.com/search?q=http://dx.doi.org/10.1186/s43058-022-00255-9)
