# Supplementary material for: Suitable Evaluation Frameworks for Disease-Agnostic Platforms for Remote Patient Monitoring: Scoping Review
Source: J Med Internet Res. 2025 Jun 16;27:e68910. doi: 10.2196/68910 (PMC12209728; doi:10.2196/68910)
Supplement: Multimedia Appendix 4 [file jmir_v27i1e68910_app4.docx]

| **No.** | **Evidence Assessment Framework for Digital Health Interventions**  Appendix 4 – Appraisal of identified frameworks against essential criteria  Framework criteria are as follows:  i Applicability not restricted to a specific population and/or therapeutic area. ii Can be applied to all domains of digital health evaluation (including clinical efficacy and effectiveness, usability, health economic assessment etc). iii Can support formative and summative evaluation (e.g. not limited to a retrospective checklist). iv Freely available to researchers and is not a proprietary tool which requires licence fees or similar. | **Year** | **Criteria** | | | | **Suitable?** | **Notes** |
| --- | --- | --- | --- | --- | --- | --- | --- | --- |
|  |  |  | **i** | **ii** | **iii** | **iv** |  |  |
| 1 | eHealth Smartphone App Evaluation (eHAPPI) framework [1] | 2024 | **Y** | **N** | **N** | **Y** | **NO** |  |
| 2 | Health navigator New Zealand [2] | 2024 | **Y** | **Y** | **N** | **Y** | **NO** |  |
| 3 | Evidence DEFINED framework [3] | 2023 | **Y** | **N** | **N** | **Y** | **NO** |  |
| 4 | FDA framework for use of real world evidence [4] | 2023 | **Y** | **N** | **N** | **Y** | **NO** |  |
| 5 | SATO (IDEAS expAnded wiTh BCIO) [5] | 2023 | **Y** | **N** | **Y** | **Y** | **NO** |  |
| 6 | Biological-Psychological, Economic, and Social (BPES) Framework [6] | 2023 | **Y** | **N** | **N** | **Y** | **NO** |  |
| 7 | 6-Step Process for Evaluating Clinical Impact [7] | 2022 | **Y** | **N** | **N** | **Y** | **NO** |  |
| 8 | Digital Therapeutics Alliance "Setting the Stage" recommendations [8] | 2022 | **Y** | **Y** | **Y** | **Y** | **YES** |  |
| 9 | Clinical robustness score [9] | 2022 | **Y** | **N** | **N** | **Y** | **NO** | Designed to appraise manufacturers |
| 10 | Multidimensional assessment framework for mobile medical apps [10] | 2022 | **Y** | **Y** | **N** | **Y** | **NO** | Recommendations for development of assessment frameworks |
| 11 | Medical Digital Solution Scoring Tool [11] | 2022 | **Y** | **N** | **N** | **Y** | **NO** |  |
| 12 | Mental Health Apps Assessment Model (Anxiety and Depression Association of America) | 2022 | **N** | **N** | **N** | **N** | **NO** | Resource no longer available. |
| 13 | Digital Health Assessment Framework [12] | 2022 | **Y** | **Y** | **N** | **N** | **NO** | Only the description of framework is available on request (full framework not available). |
| 14 | DTx Value Assessment and Integration Guide[13] | 2022 | **Y** | **Y** | **N** | **Y** | **NO** |  |
| 15 | App Rating Inventory Checklist [14] | 2022 | **Y** | **N** | **N** | **Y** | **NO** |  |
| 16 | Framework to Assist Stakeholders in Technology Evaluation for Recovery (FASTER) to Mental Health and Wellness [15] | 2022 | **N** | **Y** | **N** | **Y** | **NO** |  |
| 17 | Review, Assess, Classify, and Evaluate (RACE) [16] | 2022 | **Y** | **Y** | **N** | **Y** | **NO** |  |
| 18 | Bespoke evaluation tool for health-related apps [17] | 2022 | **Y** | **N** | **N** | **Y** | **NO** |  |
| 19 | Modified Enlight Suite [18] | 2022 | **Y** | **N** | **N** | **Y** | **NO** |  |
| 20 | Bespoke evaluation framework for breast cancer apps [19] | 2022 | **Y** | **N** | **N** | **Y** | **NO** |  |
| 21 | Bespoke framework to assess strengths and weaknesses of mhealth apps for diverse, low income populations [20] | 2022 | **Y** | **N** | **N** | **Y** | **NO** |  |
| 22 | App Script Score [21] | 2021 | **Y** | **N** | **N** | **N** | **NO** | Requires subscription to access |
| 23 | New Value Framework [22] | 2021 | **Y** | **Y** | **N** | **Y** | **NO** |  |
| 24 | Review of criteria used to evaluate health apps [23] | 2021 | **Y** | **Y** | **N** | **Y** | **NO** | Recommendations for development of a framework |
| 25 | ISO/TS 82304-2:2021(en) Health software - Part 2: Health and wellness apps - Quality and reliability [24] | 2021 | **Y** | **N** | **N** | **Y** | **NO** |  |
| 26 | Adapted Mobile Application Rating Scale (A-MARS) [25] | 2021 | **Y** | **Y** | **N** | **Y** | **NO** |  |
| 27 | App Quality Assessment Scoring System (AQASS) [26] | 2021 | **N** | **Y** | **N** | **Y** | **NO** |  |
| 28 | Vendor Evaluation framework [27] | 2021 | **Y** | **Y** | **N** | **Y** | **NO** | Designed to appraise vendors of technology |
| 29 | Value-Drive Framework for Evaluating Healthcare Innovations [28] | 2021 | **Y** | **N** | **N** | **Y** | **NO** |  |
| 30 | ACCU3RATE [29] | 2021 | **Y** | **N** | **N** | **Y** | **NO** |  |
| 31 | Digital Technology Assessment Criteria (DTAC)[30] | 2021 | **Y** | **Y** | **N** | **Y** | **NO** | References other frameworks and standards to cover multiple domains of digital health |
| 32 | Mhealth Index and Navigation Database (MIND)[31] | 2021 | **N** | **Y** | **N** | **Y** | **NO** |  |
| 33 | mhealth Atlas [32] | 2021 | **Y** | **N** | **N** | **Y** | **NO** |  |
| 34 | Mindtech co-design toolkit [33] | 2021 | **N** | **N** | **N** | **N** | **NO** | Original toolkit no longer available |
| 35 | iSYScore Index [34] | 2021 | **Y** | **N** | **N** | **Y** | **NO** |  |
| 36 | Six Aims for Quality Improvement [35] | 2021 | **Y** | **N** | **N** | **Y** | **NO** |  |
| 37 | mobile health application evaluation system (MHAES) [36] | 2021 | **Y** | **N** | **N** | **Y** | **NO** |  |
| 38 | Health technology assessment framework for Public Health interventions [37] | 2021 | **Y** | **Y** | **N** | **Y** | **NO** |  |
| 39 | 7-step framework for evaluating mobile devices [38] | 2021 | **Y** | **N** | **N** | **Y** | **NO** |  |
| 40 | Evidence-based content rating tool of mobile health applications (EBCRT-mHealth) [39] | 2021 | **Y** | **N** | **N** | **Y** | **NO** |  |
| 41 | Digital Health Indicator [40] | 2020 | **Y** | **N** | **N** | **N** | **NO** | Not accessible without booked consultation. |
| 42 | Benefit-Risk and Value Assessment Guide for Digital Health [41] | 2020 | **Y** | **N** | **N** | **Y** | **NO** |  |
| 43 | App Quality Assessment Tool (AQUA) [42] | 2020 | **Y** | **N** | **N** | **Y** | **NO** |  |
| 44 | THESIS Mobile Health Application Rating Tool [43] | 2020 | **Y** | **N** | **N** | **Y** | **NO** |  |
| 45 | Unnamed guide to Health intervention assessment [44] | 2020 | **Y** | **Y** | **N** | **Y** | **NO** | Summarises a range of evaluation models |
| 46 | Technology Evaluation and Assessment Criteria for Health Apps (TEACH-Apps) [45] | 2020 | **Y** | **N** | **N** | **Y** | **NO** |  |
| 47 | A Framework for Evaluation of Mobile Apps for Youth Mental Health [46] | 2020 | **N** | **Y** | **N** | **Y** | **NO** |  |
| 48 | Digital Health Applications Ordinance- DiGA [47] | 2020 | **Y** | **Y** | **N** | **Y** | **NO** |  |
| 49 | Unnamed framework for health technology assessment of mobile medical applications [48] | 2020 | **Y** | **Y** | **N** | **Y** | **NO** |  |
| 50 | Unnamed framework for selection of "credible" mHealth apps [49] | 2020 | **N** | **N** | **N** | **Y** | **NO** |  |
| 51 | Multi-attribute decision making framework for prioritization of mobile health applications [50] | 2020 | **Y** | **Y** | **N** | **Y** | **NO** |  |
| 52 | Australian National Safety and Quality Digital Mental Health Standards [51] | 2020 | **N** | **Y** | **N** | **Y** | **NO** |  |
| 53 | A decision-making checklist for selection of digital health technologies [52] | 2020 | **Y** | **N** | **N** | **Y** | **NO** |  |
| 54 | A digital health assessment framework from an epidemiological and legal perspective [53] | 2020 | **N** | **N** | **N** | **Y** | **NO** |  |
| 55 | Unnamed set of questions for assessment of mHealth apps [54] | 2020 | **Y** | **N** | **N** | **N** | **NO** | Recommendations for development of a framework |
| 56 | Development guide for mhealth apps [55] | 2020 | **Y** | **N** | **N** | **Y** | **NO** |  |
| 57 | Assessment of the transparency and reliability of health information dissemination [56] | 2020 | **Y** | **N** | **N** | **Y** | **NO** |  |
| 58 | Medical mobile app classification [57] | 2020 | **Y** | **N** | **N** | **Y** | **NO** | Focuses on the taxonomy of digital tools |
| 59 | Framework for digital support for the autism community [58] | 2020 | **Y** | **N** | **N** | **Y** | **NO** |  |
| 60 | Alberta rating index [59] | 2020 | **Y** | **N** | **N** | **Y** | **NO** |  |
| 61 | Express Scripts Digital Health Formulary [60] | 2020 | **N** | **N** | **N** | **N** | **NO** | Requires user account to access |
| 62 | Medical app checker [61] | 2020 | **Y** | **N** | **N** | **Y** | **NO** |  |
| 63 | Modernizing and Designing Evaluation Frameworks [62] | 2020 | **N** | **Y** | **N** | **Y** | **NO** |  |
| 64 | Unnamed framework for chatbot development and evaluation [63] | 2020 | **Y** | **N** | **N** | **Y** | **NO** |  |
| 65 | Verification, analytical validation, and clinical validation (V3) Framework [64] | 2020 | **Y** | **N** | **N** | **Y** | **NO** |  |
| 66 | Patient Education Materials Assessment Tool (PEMAT) [65] | 2020 | **Y** | **N** | **N** | **Y** | **NO** |  |
| 67 | Utility Factor Score [66] | 2020 | **Y** | **N** | **N** | **Y** | **NO** |  |
| 68 | Mobile App Rubric for Learning (MARuL) [67] | 2020 | **Y** | **N** | **N** | **Y** | **NO** |  |
| 69 | Peterson Health Technology Institute value assessment framework for digital health technologies [68] | 2020 | **Y** | **Y** | **N** | **Y** | **NO** |  |
| 70 | Bespoke evaluation criteria [69] | 2020 | **Y** | **Y** | **N** | **Y** | **NO** |  |
| 71 | Iterative decision-making for evaluation of adaptations (IDEA) [70] | 2020 | **Y** | **Y** | **Y** | **Y** | **YES** |  |
| 72 | iMedical [71] | 2020 | **Y** | **N** | **N** | **N** | **NO** | Review of apps but no detail on methodology |
| 73 | App Evaluation Model, American Psychiatric Association [72] | 2019 | **Y** | **Y** | **N** | **Y** | **NO** |  |
| 74 | Digital Assessment Questionnaire for Health Apps V2.2 [73] | 2019 | **Y** | **Y** | **N** | **Y** | **NO** | Vendor checklist |
| 75 | mHealth App Guidelines [74] | 2019 | **Y** | **Y** | **N** | **Y** | **NO** |  |
| 76 | Unnamed framework [75] | 2019 | **Y** | **N** | **N** | **Y** | **NO** |  |
| 77 | Digi-HTA: Health technology assessment framework for digital healthcare services [76] | 2019 | **Y** | **Y** | **N** | **Y** | **NO** |  |
| 78 | Digital Health Scorecard [77] | 2019 | **Y** | **Y** | **N** | **Y** | **NO** |  |
| 79 | Design and Evaluation of Digital Health Interventions (DEDHI) [78] | 2019 | **Y** | **Y** | **Y** | **Y** | **YES** |  |
| 80 | Unnamed framework for mHealth app assessment [79] | 2019 | **Y** | **N** | **N** | **Y** | **NO** |  |
| 81 | Transparency for Trust (T4T) Principles [80] | 2019 | **Y** | **N** | **N** | **Y** | **NO** |  |
| 82 | Cambridge health alliance tool [81] | 2019 | **N** | **N** | **N** | **Y** | **NO** |  |
| 83 | mHealth App Trustworthiness Checklist (mHAT) [82] | 2019 | **Y** | **N** | **N** | **Y** | **NO** |  |
| 84 | Framework for evaluating apps for adolescent users [83] | 2019 | **Y** | **N** | **N** | **Y** | **NO** |  |
| 85 | App Behavior Change Scale (ABACUS) [84] | 2019 | **Y** | **N** | **N** | **Y** | **NO** |  |
| 86 | Assessment Framework for Quality of Asthma Smartphone Applications [85] | 2019 | **N** | **N** | **N** | **Y** | **NO** |  |
| 87 | Integrate, Design, Assess, Share (IDEAS) framework [86] | 2019 | **Y** | **N** | **Y** | **Y** | **NO** |  |
| 88 | Quality principles of apps [87] | 2019 | **Y** | **N** | **N** | **Y** | **NO** |  |
| 89 | Digital health benefit evaluation (BE) framework [88] | 2019 | **Y** | **N** | **N** | **Y** | **NO** |  |
| 90 | Extended Clinical Adoption Framework [89] | 2019 | **Y** | **N** | **N** | **Y** | **NO** |  |
| 91 | WHO guideline recommendations on digital interventions for health system strengthening [90] | 2019 | **Y** | **Y** | **N** | **Y** | **NO** |  |
| 92 | Evidence Standards for Digital Health Technologies [91] | 2018 | **Y** | **Y** | **N** | **Y** | **NO** |  |
| 93 | Unnamed framework [92] | 2018 | **Y** | **N** | **N** | **Y** | **NO** |  |
| 94 | Unnamed framework [93] | 2018 | **Y** | **Y** | **N** | **Y** | **NO** | Summarises assessment domains for mhealth evaluation |
| 95 | Mobile Health Practice Guide [94] | 2018 | **Y** | **Y** | **N** | **Y** | **NO** |  |
| 96 | Toolkit for e-Mental Health Implementation [95] | 2018 | **N** | **Y** | **N** | **Y** | **NO** |  |
| 97 | Assessment Framework for e-Mental Health Apps in Canada [96] | 2018 | **Y** | **N** | **N** | **Y** | **NO** |  |
| 98 | Unnamed set of criteria for mHealth app assessment [97] | 2018 | **Y** | **N** | **N** | **Y** | **NO** | Recommendations for how clinicians should engage with manufacturers of digital health solutions |
| 99 | Quality assurance for user protection | 2018 | **N** | **N** | **N** | **N** | **NO** | Resource no longer available |
| 100 | DiaDigital app evaluation criteria [98] | 2018 | **N** | **N** | **N** | **Y** | **NO** |  |
| 101 | Graded review of dermatology apps [99] | 2018 | **Y** | **N** | **N** | **Y** | **NO** |  |
| 102 | Mobile applications recommendations [100] | 2018 | **Y** | **N** | **N** | **Y** | **NO** |  |
| 103 | Trust4app [101] | 2018 | **Y** | **N** | **N** | **Y** | **NO** |  |
| 104 | Evaluation criteria for noninvasive telemonitoring [102] | 2018 | **Y** | **Y** | **N** | **Y** | **NO** | Designed for hardware technologies |
| 105 | 6 steps in quality intervention development (6SQuID) [103] | 2018 | **Y** | **Y** | **Y** | **Y** | **YES** |  |
| 106 | Unnamed framework [104] | 2018 | **Y** | **Y** | **N** | **Y** | **NO** |  |
| 107 | Unnamed survey [105] | 2018 | **Y** | **N** | **N** | **Y** | **NO** |  |
| 108 | MedAd-AppQ ( Medication Adherence App Quality assessment tool) [106] | 2018 | **Y** | **N** | **N** | **Y** | **NO** |  |
| 109 | Framework for the Effectiveness Evaluation of Mobile (Mental) Health Tools [107] | 2017 | **Y** | **Y** | **Y** | **Y** | **YES** |  |
| 110 | Enlight Evaluation Tool for Mobile and Web-Based eHealth Interventions [108] | 2017 | **Y** | **N** | **N** | **Y** | **NO** |  |
| 111 | ORCHA-24 Framework [109] | 2017 | **Y** | **N** | **N** | **Y** | **NO** |  |
| 112 | Set of heuristic methods for evaluation of digital health interventions [110] | 2017 | **Y** | **N** | **N** | **Y** | **NO** |  |
| 113 | Assessment tool for STI apps [111] | 2017 | **N** | **N** | **N** | **Y** | **NO** |  |
| 114 | Diabetes evaluation tool [112] | 2017 | **N** | **N** | **N** | **Y** | **NO** |  |
| 115 | Quality assurance tool for research [113] | 2017 | **Y** | **N** | **N** | **Y** | **NO** | Describes guiding principles to evaluation |
| 116 | Usability evaluation tool [114] | 2017 | **Y** | **N** | **N** | **Y** | **NO** |  |
| 117 | APPLICATIONS scoring system [115] | 2017 | **Y** | **N** | **N** | **Y** | **NO** |  |
| 118 | NASSS: New Framework for Theorizing and Evaluating Nonadoption, Abandonment, and Challenges to the Scale-Up, Spread, and Sustainability of Health and Care Technologies. [116] | 2017 | **Y** | **Y** | **N** | **Y** | **NO** |  |
| 119 | App Quality Evaluation Tool (AQEL) [117] | 2017 | **N** | **N** | **N** | **Y** | **NO** |  |
| 120 | Self-Management Framework for Evaluating Interactive App Functions (SFEIAF) [118] | 2017 | **N** | **N** | **N** | **Y** | **NO** |  |
| 121 | Constructive eHealth evaluation method (CeHEM) [119] | 2017 | **Y** | **N** | **Y** | **Y** | **NO** |  |
| 122 | Health-related mobile app evaluation criteria [120] | 2017 | **Y** | **N** | **N** | **Y** | **NO** |  |
| 123 | Adapted checklist for assessment of mobile apps [121] | 2016 | **Y** | **N** | **N** | **Y** | **NO** |  |
| 124 | Pictorial identification schema for diabetes self-care apps [122] | 2016 | **N** | **N** | **N** | **Y** | **NO** |  |
| 125 | Key questions for appraisal of digital health interventions [123] | 2016 | **Y** | **Y** | **N** | **Y** | **NO** |  |
| 126 | Mobile Health (mHealth) Evidence Reporting and Assessment (mERA) checklist [124] | 2016 | **Y** | **Y** | **N** | **Y** | **NO** |  |
| 127 | Good Practice Guidelines on Health Apps and Smart Devices [125] | 2016 | **Y** | **N** | **N** | **Y** | **NO** |  |
| 128 | Interactive Mobile App Review Toolkit (IMART) [126] | 2016 | **Y** | **N** | **N** | **Y** | **NO** |  |
| 129 | Unnamed set of criteria for assessment of mobile medical applications in diabetes [127] | 2016 | **N** | **N** | **N** | **Y** | **NO** |  |
| 130 | Unnamed adaptation of NICE behaviour change guidance for app quality assessment [128] | 2016 | **Y** | **N** | **N** | **Y** | **NO** |  |
| 131 | Bespoke quality assessment scale combining Silberg Scale and Technology Assessment Model [129] | 2016 | **N** | **N** | **N** | **Y** | **NO** |  |
| 132 | Bespoke evaluation tool [130] | 2016 | **Y** | **N** | **N** | **Y** | **NO** |  |
| 133 | uMARS [131] | 2016 | **Y** | **Y** | **N** | **Y** | **NO** |  |
| 134 | Adaption of the CRAAP (Currency, Relevancy, Accuracy, Authority, Purpose) model [132] | 2016 | **Y** | **N** | **N** | **Y** | **NO** |  |
| 135 | Tool to assess medication related problems [133] | 2016 | **N** | **N** | **N** | **Y** | **NO** |  |
| 136 | Checklist for app dealing with chronic disease [134] | 2016 | **Y** | **N** | **N** | **Y** | **NO** |  |
| 137 | Tool to appraise TB apps [135] | 2016 | **Y** | **N** | **N** | **Y** | **NO** |  |
| 138 | Certification programme [136] | 2016 | **Y** | **N** | **N** | **Y** | **NO** |  |
| 139 | mhealth evaluation method [137] | 2016 | **Y** | **Y** | **N** | **Y** | **NO** |  |
| 140 | Evaluation of mhealth pain apps [138] | 2016 | **Y** | **N** | **N** | **Y** | **NO** |  |
| 141 | mhealth rating measure [139] | 2016 | **Y** | **N** | **N** | **Y** | **NO** |  |
| 142 | Quality assurance tool for engagement, quality and safety of mobile health applications [140] | 2016 | **Y** | **N** | **N** | **Y** | **NO** |  |
| 143 | AMA principles to promote safe effective mhealth applications [141] | 2016 | **Y** | **N** | **N** | **Y** | **NO** |  |
| 144 | Educational intervention mapping [142] | 2016 | **Y** | **N** | **Y** | **Y** | **NO** |  |
| 145 | User centred design approach [143] | 2016 | **Y** | **N** | **N** | **Y** | **NO** |  |
| 146 | Cost quality and access indicators for telemedicine implementation [144] | 2016 | **Y** | **N** | **N** | **Y** | **NO** |  |
| 147 | Currency, Relevancy, Accuracy, Authority, Purpose Test (CRAAP) [145] | 2016 | **Y** | **N** | **N** | **Y** | **NO** |  |
| 148 | Bespoke evaluation framework [146] | 2016 | **Y** | **N** | **N** | **Y** | **NO** |  |
| 149 | WHO guideline on monitoring and evaluating DHIs [147] | 2016 | **Y** | **Y** | **Y** | **Y** | **YES** |  |
| 150 | IDEAL-D: Idea, Development, Exploration, Assessment, Long term study (device) [148] | 2016 | **Y** | **Y** | **N** | **Y** | **NO** |  |
| 151 | CRAAP test (Currency, Relevance, Authority, Accuracy, and Purpose of information) [149] | 2016 | **Y** | **N** | **N** | **Y** | **NO** |  |
| 152 | Mobile Application Rating Scale (MARS) [150] | 2015 | **Y** | **Y** | **N** | **Y** | **NO** |  |
| 153 | Publicly available specification 277:2015 Health and wellness apps – quality criteria across the life cycle [151] | 2015 | **Y** | **Y** | **N** | **Y** | **NO** | Withdrawn Oct 2023 |
| 154 | Royal College of Physicians (RCP) Health Informatics Unit Checklist [152] | 2015 | **Y** | **Y** | **N** | **Y** | **NO** |  |
| 155 | Unnamed set of questions for assessment of mobile mental health apps [153] | 2015 | **Y** | **N** | **N** | **Y** | **NO** |  |
| 156 | ISO/IEC 9126-1 usability model [154] | 2015 | **Y** | **N** | **N** | **Y** | **NO** |  |
| 157 | Bespoke evaluation tool [155] | 2015 | **Y** | **N** | **N** | **Y** | **NO** |  |
| 158 | Tool to assess quality of mHealth app [156] | 2015 | **Y** | **N** | **N** | **Y** | **NO** |  |
| 159 | Quality assessment tool based on Health-related website evaluation tool [157] | 2015 | **Y** | **N** | **N** | **Y** | **NO** |  |
| 160 | Security and Safety measure tool [158] | 2015 | **Y** | **N** | **N** | **Y** | **NO** |  |
| 161 | Quality assessment tool for mhealth apps [159] | 2015 | **Y** | **N** | **N** | **Y** | **NO** |  |
| 162 | Quality assessment tool for patients using health apps [160] | 2015 | **N** | **N** | **N** | **Y** | **NO** |  |
| 163 | Quality assurance tool for research settings [161] | 2015 | **N** | **N** | **N** | **Y** | **NO** |  |
| 164 | Clinical guideline [162] | 2015 | **Y** | **N** | **N** | **Y** | **NO** |  |
| 165 | Appraisal of melanoma mhealth tools [163] | 2015 | **N** | **N** | **N** | **Y** | **NO** |  |
| 166 | Review of pain management apps [164] | 2015 | **N** | **N** | **N** | **Y** | **NO** |  |
| 167 | Concussion app development guidelines [165] | 2015 | **N** | **N** | **N** | **Y** | **NO** |  |
| 168 | mhealth evaluation tool [166] | 2015 | **N** | **N** | **N** | **Y** | **NO** |  |
| 169 | Quality assurance tool for research [167] | 2015 | **N** | **N** | **N** | **Y** | **NO** |  |
| 170 | NPMEDAPP (Novel, Potential of Benefit vs Risk, Medically sound, Ease of use, Developer, Audience, Price, Platform) [168] | 2015 | **Y** | **Y** | **N** | **Y** | **NO** |  |
| 171 | Trial of intervention principles framework [169] | 2015 | **Y** | **Y** | **Y** | **Y** | **YES** |  |
| 172 | TECH model (TElehealth in CHronic Disease) [170] | 2015 | **Y** | **N** | **N** | **Y** | **NO** |  |
| 173 | Unnamed framework [171] | 2015 | **Y** | **Y** | **N** | **Y** | **NO** |  |
| 174 | Unnamed set of recommendations for assessment of mobile health apps [172] | 2014 | **Y** | **N** | **N** | **Y** | **NO** | Strategies for consumer to select mobile applications |
| 175 | Unnamed set of recommendations for assessment of mobile health apps [173] | 2014 | **Y** | **N** | **N** | **Y** | **NO** |  |
| 176 | Evaluating Mobile Medical Applications Checklist [174] | 2014 | **Y** | **N** | **N** | **Y** | **NO** |  |
| 177 | Heuristic evaluation [175] | 2014 | **N** | **N** | **N** | **Y** | **NO** |  |
| 178 | Usability questionnaire [176] | 2014 | **Y** | **N** | **N** | **Y** | **NO** |  |
| 179 | Quality assurance of mobile apps [177] | 2014 | **N** | **N** | **N** | **Y** | **NO** |  |
| 180 | Tool to appraise breast applications [178] | 2014 | **N** | **N** | **N** | **Y** | **NO** |  |
| 181 | HIV app evaluation tool [179] | 2014 | **Y** | **N** | **N** | **Y** | **NO** |  |
| 182 | evaluation tool for patients [180] | 2014 | **Y** | **N** | **N** | **Y** | **NO** |  |
| 183 | Telehealth delivery barriers [181] | 2014 | **Y** | **N** | **N** | **Y** | **NO** | List of barriers rather than an evaluation framework |
| 184 | Barriers and facilitators to therapeutic alliance [182] | 2014 | **Y** | **N** | **N** | **Y** | **NO** |  |
| 185 | Mobile medical evaluation rubric [174] | 2014 | **Y** | **Y** | **N** | **Y** | **NO** |  |
| 186 | Clinical adoption meta-model (CAMM) [183] | 2014 | **Y** | **Y** | **N** | **Y** | **NO** | Designed to guide intervention evaluation conversations. |
| 187 | APEASE: affordability, practicability, effectiveness and cost-effectiveness, acceptability, side-effects and safety, equity. | 2014 | **Y** | **N** | **N** | **Y** | **NO** |  |
| 188 | PsyberGuide [184] | 2013 | **Y** | **Y** | **N** | **Y** | **NO** |  |
| 189 | Synopsis for Health Apps [185] | 2013 | **Y** | **N** | **N** | **Y** | **NO** |  |
| 190 | Framework for assessment of mobile applications for cardiac rehab [186] | 2013 | **N** | **N** | **N** | **Y** | **NO** |  |
| 191 | PANAsian Collaboration for Evidence-Based eHealth Adoption and Application [187] | 2013 | **Y** | **Y** | **N** | **Y** | **NO** |  |
| 192 | Health IT Usability Evaluation Model [188] | 2013 | **Y** | **N** | **N** | **Y** | **NO** |  |
| 193 | Quality of experience tool [189] | 2013 | **Y** | **N** | **N** | **Y** | **NO** |  |
| 194 | Quality assurance for user protection [190] | 2013 | **Y** | **N** | **N** | **Y** | **NO** |  |
| 195 | Evaluation tool for mobile applications [191] | 2013 | **Y** | **N** | **N** | **Y** | **NO** |  |
| 196 | Cancer information sources on mobile [192] | 2013 | **Y** | **N** | **N** | **Y** | **NO** |  |
| 197 | Designing for behaviour change [193] | 2013 | **Y** | **N** | **N** | **Y** | **NO** |  |
| 198 | e-health service implementation factors [194] | 2013 | **Y** | **Y** | **N** | **Y** | **NO** |  |
| 199 | Barriers to diabetes telehealth [195] | 2013 | **N** | **N** | **N** | **Y** | **NO** | List of barriers rather than an evaluation framework |
| 200 | Summary of effectiveness of telehealth delivery of evidence-based psychotherapies [196] | 2013 | **Y** | **N** | **N** | **Y** | **NO** | List of barriers rather than an evaluation framework |
| 201 | Behaviour Change Technique Taxonomy Version One (BCTTv1) [197] | 2013 | **Y** | **N** | **N** | **Y** | **NO** |  |
| 202 | Model for Assessment of Telemedicine applications (MAST) [198] | 2012 | **Y** | **Y** | **N** | **Y** | **NO** |  |
| 203 | Asthma self-management appraisal tool [199] | 2012 | **Y** | **N** | **N** | **Y** | **NO** |  |
| 204 | Quality assurance tool for research settings [200] | 2012 | **N** | **N** | **N** | **Y** | **NO** |  |
| 205 | Usability evaluation tool [201] | 2012 | **Y** | **N** | **N** | **Y** | **NO** |  |
| 206 | Medical app evaluation tool [202] | 2012 | **Y** | **N** | **N** | **Y** | **NO** | Guidance for consumers to identify appropriate mobile apps for their needs |
| 207 | Factors that hinder or support telemedicine implementation [203] | 2012 | **Y** | **N** | **N** | **Y** | **NO** | List of enablers and barriers rather than an evaluation framework |
| 208 | Appraisal of telehealth services for paediatric audiology [204] | 2012 | **N** | **N** | **N** | **Y** | **NO** |  |
| 209 | Self-developed checklist [205] | 2012 | **Y** | **N** | **N** | **Y** | **NO** |  |
| 210 | CONSORT-eHealth Checklist Extension [206] | 2011 | **Y** | **Y** | **N** | **Y** | **NO** |  |
| 211 | Instrument developed by Gan and Allman-Farinelli [207] | 2011 | **N** | **N** | **N** | **Y** | **NO** |  |
| 212 | Quality assurance tool for research settings [208] | 2011 | **N** | **N** | **N** | **Y** | **NO** |  |
| 213 | Behaviour Change Wheel [209] | 2011 | **Y** | **N** | **N** | **Y** | **NO** |  |
| 214 | Coventry, Aberdeen and London-Revised taxonomy (CALO-RE) [210] | 2011 | **Y** | **N** | **N** | **Y** | **NO** |  |
| 215 | Pragmatic health information technology evaluation framework [211] | 2011 | **Y** | **N** | **N** | **Y** | **NO** |  |
| 216 | Outcomes for implementation research [212] | 2011 | **Y** | **Y** | **N** | **Y** | **NO** |  |
| 217 | User experience assessment framework [213] | 2010 | **Y** | **N** | **N** | **Y** | **NO** |  |
| 218 | Privacy framework [214] | 2009 | **Y** | **N** | **N** | **Y** | **NO** |  |
| 219 | Web information assessment tool [215] | 2009 | **Y** | **N** | **N** | **Y** | **NO** |  |
| 220 | Consolidated Framework for Implementation Research (CFIR) [216] | 2009 | **Y** | **Y** | **Y** | **Y** | **YES** |  |
| 221 | Framework for continuous systemic evaluation for Health IT systems [217] | 2009 | **Y** | **N** | **Y** | **Y** | **NO** |  |
| 222 | Clinical adoption framework (CAF) [218] | 2009 | **Y** | **Y** | **Y** | **Y** | **YES** |  |
| 223 | Proctor's implementation science framework [219] | 2009 | **Y** | **N** | **Y** | **Y** | **NO** |  |
| 224 | Normalisation Process Theory (NPT) [220] | 2009 | **Y** | **N** | **N** | **Y** | **NO** | More suited to assess how practices are embedded and implemented |
| 225 | Behaviour Change Taxonomy (26 item) [221] | 2008 | **Y** | **N** | **N** | **Y** | **NO** |  |
| 226 | Human, organization and technology-fit factors (HOT-fit) [222] | 2008 | **Y** | **Y** | **N** | **Y** | **NO** |  |
| 227 | Health technology assessment framework [223] | 2008 | **Y** | **Y** | **N** | **Y** | **NO** |  |
| 228 | Practical, Robust Implementation and Sustainability Model (PRISM) [224] | 2008 | **Y** | **Y** | **N** | **Y** | **NO** |  |
| 229 | Information Systems Success Framework (ISS) [225] | 2008 | **Y** | **Y** | **N** | **Y** | **NO** |  |
| 230 | Revised MRC Framework [226] | 2007 | **Y** | **Y** | **Y** | **Y** | **YES** |  |
| 231 | Multiphase optimisation strategy [227] | 2007 | **Y** | **Y** | **Y** | **Y** | **YES** |  |
| 232 | Benefits evaluation framework [228] | 2007 | **Y** | **Y** | **N** | **Y** | **NO** |  |
| 233 | Sequential Multiple Assignment Randomized Trial (SMART) [227] | 2007 | **Y** | **Y** | **Y** | **Y** | **YES** |  |
| 234 | Usability evaluation checklist [229] | 2006 | **Y** | **N** | **N** | **Y** | **NO** |  |
| 235 | Healthcare Website Assessment Instrument [230] | 2006 | **Y** | **N** | **N** | **Y** | **NO** |  |
| 236 | PRECEDE-PROCEED Model [231] | 2005 | **Y** | **Y** | **N** | **Y** | **NO** |  |
| 237 | DISCERN [232] | 2004 | **Y** | **N** | **N** | **Y** | **NO** |  |
| 238 | Website quality assessment criteria [233] | 2004 | **Y** | **N** | **N** | **Y** | **NO** |  |
| 239 | Promoting action on research implementation (PARIHS) [234] | 2004 | **Y** | **N** | **N** | **Y** | **NO** |  |
| 240 | Modified ICT implementation framework [235] | 2003 | **Y** | **N** | **N** | **Y** | **NO** |  |
| 241 | International Network of Agencies for Health Technology Assessment (INAHTA) checklist [236] | 2003 | **Y** | **Y** | **N** | **Y** | **NO** |  |
| 242 | Process evaluation for Public Health Interventions and Research [237] | 2002 | **Y** | **N** | **N** | **N** | **NO** |  |
| 243 | Modified Silberg score [238] | 2000 | **Y** | **N** | **N** | **Y** | **NO** |  |
| 244 | Abbott's scale [239] | 2000 | **Y** | **N** | **N** | **Y** | **NO** |  |
| 245 | MRC complex intervention framework [240] | 2000 | **Y** | **Y** | **Y** | **Y** | **YES** |  |
| 246 | Reach Effectiveness Adoption Implementation and Maintenance (RE-AIM) framework [241] | 1999 | **Y** | **Y** | **Y** | **Y** | **YES** |  |
| 247 | AHRQ (Agency for Healthcare Research and Quality) criteria [242] | 1999 | **Y** | **N** | **N** | **Y** | **NO** |  |
| 248 | Jones instrument [243] | 1999 | **Y** | **N** | **N** | **Y** | **NO** |  |
| 249 | Health on the net [244] | 1998 | **Y** | **N** | **N** | **Y** | **NO** |  |
| 250 | Intervention planning framework [245] | 1998 | **Y** | **N** | **N** | **Y** | **NO** |  |
| 251 | Educational intervention mapping [246] | 1998 | **Y** | **N** | **Y** | **Y** | **NO** |  |
| 252 | Silberg scale for accountability [247] | 1997 | **Y** | **N** | **N** | **Y** | **NO** |  |
| 253 | ADDIE (analysis, design, development, implementation, and evaluation) [248] | 1975 | **Y** | **Y** | **Y** | **Y** | **YES** |  |
| 254 | CredibleMind [249] | Unknown | **N** | **N** | **N** | **N** | **NO** | Not accessible without booked consultation |

References:

1. Ribaut J, DeVito Dabbs A, Dobbels F, Teynor A, Mess EV, Hoffmann T, et al. Developing a Comprehensive List of Criteria to Evaluate the Characteristics and Quality of eHealth Smartphone Apps: Systematic Review. JMIR Mhealth Uhealth. 2024 Jan 15;12:e48625. PMID: 38224477. doi: 10.2196/48625.
2. Zealand HN. Health applications assessment guidance. 2024 [cited 2024 6 November]; Available from: <https://www.tewhatuora.govt.nz/health-services-and-programmes/digital-health/other-digital-health-initiatives/health-applications-assessment-guidance>.
3. Silberman J, Wicks P, Patel S, Sarlati S, Park S, Korolev IO, et al. Rigorous and rapid evidence assessment in digital health with the evidence DEFINED framework. NPJ Digit Med. 2023 May 31;6(1):101. PMID: 37258851. doi: 10.1038/s41746-023-00836-5.
4. Fda US. Framework for FDA’s real-world evidence program. Silver Spring, MD: US Department of Health and Human Services Food and Drug Administration. 2018.
5. Lisowska A, Wilk S, Peleg M. SATO (IDEAS expAnded wiTh BCIO): Workflow for designers of patient-centered mobile health behaviour change intervention applications. Journal of biomedical informatics. 2022:104276. PMID: 639923583. doi: [https://dx.doi.org/10.1016/j.jbi.2022.104276](https://www.google.com/search?q=https://dx.doi.org/10.1016/j.jbi.2022.104276).
6. Khan WU, Seto E. A "Do No Harm" Novel Safety Checklist and Research Approach to Determine Whether to Launch an Artificial Intelligence-Based Medical Technology: Introducing the Biological-Psychological, Economic, and Social (BPES) Framework. J Med Internet Res. 2023 Apr 5;25:e43386. PMID: 37018019. doi: 10.2196/43386.
7. Mathews S, Prime M. Evaluating digital health solutions: How to know which solutions will bring value to your organization?: Healthcare Transformers; 2023 [cited 2024 10 October]; Available from: <https://healthcaretransformers.com/digital-health/current-trends/evaluating-digital-health-solutions-event/>.
8. Alliance DT. Setting the Stage for a Fit-For-Purpose DTX Evidentiary Standard. 2022.
9. Day S, Shah V, Kaganoff S, Powelson S, Mathews SC. Assessing the clinical robustness of digital health startups: cross-sectional observational analysis. Journal of medical Internet research. 2022;24(6):e37677.
10. Tarricone R, Petracca F, Cucciniello M, Ciani O. Recommendations for developing a lifecycle, multidimensional assessment framework for mobile medical apps. Health Economics (United Kingdom). 2022;31(S1):73-97. PMID: 2015558935. doi: [https://dx.doi.org/10.1002/hec.4505](https://www.google.com/search?q=https://dx.doi.org/10.1002/hec.4505).
11. Wagneur N, Callier P, Zeitoun J-D, Silber D, Sabatier R, Denis F. Assessing a new prescreening score for the simplified evaluation of the clinical quality and relevance of eHealth apps: instrument validation study. Journal of Medical Internet Research. 2022;24(7):e39590.
12. (ORCHA) OftRoCaHA. Digital Health Assessment Framework. 2022 [cited 2024 10 October]; Available from: <https://orchahealth.com/our-products/assessment-frameworks/digital-health-assessment-framework-dhaf/>.
13. Alliance DT. DTx Value Assessment & Integration Guide Version 2.0. Digital Therapeutics Alliance; 2022 [cited 2024 10 October]; Available from: <https://dtxalliance.org/advancing-dtx/dtx-value-guide/>.
14. Mackey R, Gleason A, Ciulla R. A Novel Method for Evaluating Mobile Apps (App Rating Inventory): Development Study. JMIR Mhealth Uhealth. 2022 Apr 15;10(4):e32643. PMID: 35436227. doi: 10.2196/32643.
15. Agarwal S, Jalan M, Wilcox HC, Sharma R, Hill R, Pantalone E, et al. AHRQ Comparative Effectiveness Technical Briefs. Evaluation of Mental Health Mobile Applications. Rockville (MD): Agency for Healthcare Research and Quality (US); 2022.
16. Varshney U, Singh N, Bourgeois AG, Dube SR. Review, Assess, Classify, and Evaluate (RACE): a framework for studying m-health apps and its application for opioid apps. J Am Med Inform Assoc. 2022 Jan 29;29(3):520-35. PMID: 34939117. doi: 10.1093/jamia/ocab277.
17. Scherer J, Youssef Y, Dittrich F, Albrecht U-V, Tsitsilonis S, Jung J, et al. Proposal of a new rating concept for digital health applications in orthopedics and traumatology. International journal of environmental research and public health. 2022;19(22):14952.
18. Woulfe F, Fadahunsi KP, O'Grady M, Chirambo GB, Mawkin M, Majeed A, et al. Modification and Validation of an mHealth App Quality Assessment Methodology for International Use: Cross-sectional and eDelphi Studies. JMIR Form Res. 2022 Aug 19;6(8):e36912. PMID: 35984688. doi: 10.2196/36912.
19. Gomm SIM, Ebner FK, Lukac S, El Taie Z, Janni W, Schmidt-Strabetaburger U, et al. Mobile Applications Available in Germany Supporting Breast Cancer Patients during Treatment and Aftercare: A Systematic Review. Geburtshilfe und Frauenheilkunde. 2022;82(9):941-54. PMID: 2020423404. doi: [https://dx.doi.org/10.1055/a-1909-8736](https://www.google.com/search?q=https://dx.doi.org/10.1055/a-1909-8736).
20. Sharma S, Gergen Barnett K, Maypole JJ, Grochow Mishuris R. Evaluation of mHealth Apps for Diverse, Low-Income Patient Populations: Framework Development and Application Study. JMIR Form Res. 2022 Feb 11;6(2):e29922. PMID: 35147502. doi: 10.2196/29922.
21. IQVIA. AppScript | discover, deliver & track digital health. 2021 [cited 2024 15 September]; Available from: <https://www.appscript.net/score-details>.
22. Augenstein J, Marks J, Savuto M, Barron M, Mishra V, Lloyd S. Return on health: moving beyond dollars and cents in realizing the value of virtual care. Association Medical Association. 2021:2021-05.
23. Hensher M, Cooper P, Dona SWA, Angeles MR, Nguyen D, Heynsbergh N, et al. Scoping review: Development and assessment of evaluation frameworks of mobile health apps for recommendations to consumers. J Am Med Inform Assoc. 2021 Jun 12;28(6):1318-29. PMID: 33787894. doi: 10.1093/jamia/ocab041.
24. Standardization IOf. Health software -part 2: health and wellness apps—quality and reliability (ISO/TS 82304-2). 1 ed: International Organization for Standardization; 2021.
25. Roberts AE, Davenport TA, Wong T, Moon H-W, Hickie IB, LaMonica HM. Evaluating the quality and safety of health-related apps and e-tools: Adapting the Mobile App Rating Scale and developing a quality assurance protocol. Internet interventions. 2021;24:100379. doi: [https://dx.doi.org/10.1016/j.invent.2021.100379](https://www.google.com/search?q=https://dx.doi.org/10.1016/j.invent.2021.100379).
26. Wu K-L, Alegria R, Gonzalez J, Hu H, Wang H, Page R, et al. Characteristics and Quality of Mobile Apps Containing Prenatal Genetic Testing Information: Systematic App Store Search and Assessment. JMIR mHealth and uHealth. 2021;9(10):e30404. doi: <https://dx.doi.org/10.2196/30404>.
27. Institute V. Vendor Evaluation: Overview of this Multi-Part Series. Validation Institute; 2021 [cited 2024 18 October]; Available from: <https://validationinstitute.com/wp-content/uploads/2021/04/Vendor-Evaluation_-Overview-of-this-Multi-Part-Series.pdf>.
28. Affairs UDoV, Society DM. Value-Driven Framework for Evaluating Healthcare Innovations. Digital Medicine Society; 2022 [cited 2024 6 November]; Available from: <https://dimesociety.org/resources/value-driven-framework-for-evaluating-healthcare-innovations/>.
29. Biswas M, Tania MH, Kaiser MS, Kabir R, Mahmud M, Kemal AA. ACCU3RATE: A mobile health application rating scale based on user reviews. PLoS One. 2021;16(12):e0258050. PMID: 34914718. doi: 10.1371/journal.pone.0258050.
30. England N. Digital Technology Assessment Criteria (DTAC). NHS England Transformation Directorate; 2024 [cited 2024 11 October]; Available from: <https://transform.england.nhs.uk/key-tools-and-info/digital-technology-assessment-criteria-dtac/>.
31. Lagan S, Sandler L, Torous J. Evaluating evaluation frameworks: a scoping review of frameworks for assessing health apps. BMJ open. 2021;11(3):e047001. doi: <https://dx.doi.org/10.1136/bmjopen-2020-047001>.
32. Lehmann NJ, Spielmann F, George B, Ververs L, Karagülle M-U, Kmiotek D, et al., editors. mHealthAtlas-an approach for the multidisciplinary evaluation of mHealth applications. 2021: IEEE.
33. MinD. Mindful Co-Design Toolkit. 2021; Available from: <https://designingfordementia.eu/resources/mind-tools/co-design-toolkit>.
34. Grau-Corral I, Pantoja PE, Grajales Iii FJ, Kostov B, Aragunde V, Puig-Soler M, et al. Assessing Apps for Health Care Workers Using the ISYScore-Pro Scale: Development and Validation Study. JMIR mHealth and uHealth. 2021;9(7):e17660. PMID: 635666840. doi: <https://dx.doi.org/10.2196/17660>.
35. Lee Y, Shin S-Y, Kim J-Y, Kim JH, Seo D-W, Joo S, et al. Evaluation of mobile health applications developed by a tertiary hospital as a tool for quality improvement breakthrough. Healthcare Informatics Research. 2015;21(4):299-306.
36. Wang X, Du K, Zhu K, Xu S, Zhang S. Where Should Mobile Health Application Providers Focus Their Goals? International Journal of Computational Intelligence Systems. 2021;14(1):1119-31.
37. Hussain MS, Silvera-Tawil D, Farr-Wharton G. Technology assessment framework for precision health applications. Int J Technol Assess Health Care. 2021 May 26;37(1):e67. PMID: 34034854. doi: 10.1017/s0266462321000350.
38. Kleckner IR, Feldman MJ, Goodwin MS, Quigley KS. Framework for selecting and benchmarking mobile devices in psychophysiological research. Behav Res Methods. 2021 Apr;53(2):518-35. PMID: 32748241. doi: 10.3758/s13428-020-01438-9.
39. Raeesi A, Khajouei R, Ahmadian L. Evaluation of HIV/AIDS-related mobile health applications content using an evidence-based content rating tool. BMC Med Inform Decis Mak. 2021 Apr 24;21(1):135. PMID: 33892691. doi: 10.1186/s12911-021-01498-7.
40. Systems HIaM. DIGITAL HEALTH INDICATOR. HIMSS; 2020 [cited 2024 11 Oct]; Available from: <https://www.himss.org/maturity-models/digital-health-indicator/>.
41. Perakslis E, Ginsburg GS. Digital health—the need to assess benefits, risks, and value. Jama. 2021;325(2):127-8.
42. O'Rourke T, Pryss R, Schlee W, Probst T. Development of a multidimensional app-quality assessment tool for health-related apps (AQUA). Digital Psychology. 2020;1(2):13-23.
43. Levine DM, Co Z, Newmark LP, Groisser AR, Holmgren AJ, Haas JS, et al. Design and testing of a mobile health application rating tool. NPJ Digital Medicine. 2020;3(1):74.
44. Ondersma SJ, Walters ST. Clinician's guide to evaluating and developing ehealth interventions for mental health. Psychiatric Research and Clinical Practice. 2020;2(1):26-33.
45. Camacho E, Hoffman L, Lagan S, Rodriguez-Villa E, Rauseo-Ricupero N, Wisniewski H, et al. Technology evaluation and assessment criteria for health apps (TEACH-Apps): Pilot study. Journal of Medical Internet Research. 2020;22(8):e18346. PMID: 2007819686. doi: <https://dx.doi.org/10.2196/18346>.
46. Quintana Y, Torous J. A framework for evaluation of mobile apps for youth mental health. Guelph, Ontario, Canada: Homewood Research Institute. 2020.
47. Kramer VvDU, Vollmar HC. Verordnung über das Verfahren und die Anforderungen der Prüfung der Erstattungsfähigkeit digitaler Gesundheitsanwendungen in der gesetzlichen Krankenversicherung.
48. Moshi MR, Tooher R, Merlin T. Development of a health technology assessment module for evaluating mobile medical applications. International journal of technology assessment in health care. 2020;36(3):252-61. doi: [https://dx.doi.org/10.1017/S0266462320000288](https://www.google.com/search?q=https://dx.doi.org/10.1017/S0266462320000288).
49. Psihogios AM, Stiles-Shields C, Neary M. The Needle in the Haystack: Identifying Credible Mobile Health Apps for Pediatric Populations during a Pandemic and beyond. J Pediatr Psychol. 2020 Nov 1;45(10):1106-13. PMID: 33068424. doi: 10.1093/jpepsy/jsaa094.
50. Noee M, Sari AA, Olyaeemanesh A, Mobinizadeh M. Prioritizing the potential applications of Mobile-Health in the Iranian health system. Journal of Research in Health Sciences. 2020;20(1):e00473.
51. Brown P, Prest B, Miles P, Rossi V. The development of National Safety and Quality Digital Mental Health Standards. Australasian Psychiatry. 2021 2022/04/01;30(2):154-7. doi: 10.1177/10398562211042361.
52. Nebeker C, Bartlett Ellis RJ, Torous J. Development of a decision-making checklist tool to support technology selection in digital health research. Transl Behav Med. 2020 Oct 8;10(4):1004-15. PMID: 31120511. doi: 10.1093/tbm/ibz074.
53. Vokinger KN, Nittas V, Witt CM, Fabrikant SI, Von Wyl V. Digital health and the COVID-19 epidemic: An assessment framework for apps from an epidemiological and legal perspective. Swiss Medical Weekly. 2020;150(19-20):w20282. PMID: 2007969875. doi: <https://dx.doi.org/10.4414/smw.2020.20282>.
54. Dawson RM, Felder TM, Donevant SB, McDonnell KK, Card EB, 3rd, King CC, et al. What makes a good health 'app'? Identifying the strengths and limitations of existing mobile application evaluation tools. Nursing inquiry. 2020;27(2):e12333. doi: [https://dx.doi.org/10.1111/nin.12333](https://www.google.com/search?q=https://dx.doi.org/10.1111/nin.12333).
55. Llorens-Vernet P, Miro J. Standards for Mobile Health-Related Apps: Systematic Review and Development of a Guide. JMIR mHealth and uHealth. 2020;8(3):e13057. PMID: 631160292. doi: <https://dx.doi.org/10.2196/13057>.
56. Huang Z, Lum E, Car J. Medication Management Apps for Diabetes: Systematic Assessment of the Transparency and Reliability of Health Information Dissemination. JMIR mHealth and uHealth. 2020;8(2):e15364. PMID: 631166230. doi: <https://dx.doi.org/10.2196/15364>.
57. Nwe K, Larsen ME, Nelissen N, Wong DC-W. Medical Mobile App Classification Using the National Institute for Health and Care Excellence Evidence Standards Framework for Digital Health Technologies: Interrater Reliability Study. Journal of medical Internet research. 2020;22(6):e17457. doi: <https://dx.doi.org/10.2196/17457>.
58. Zervogianni V, Fletcher-Watson S, Herrera G, Goodwin M, Perez-Fuster P, Brosnan M, et al. A framework of evidence-based practice for digital support, co-developed with and for the autism community. Autism. 2020;24(6):1411-22. doi: [https://dx.doi.org/10.1177/1362361319898331](https://www.google.com/search?q=https://dx.doi.org/10.1177/1362361319898331).
59. Azad-Khaneghah P, Roduta Roberts M, Liu L. Alberta Rating Index for Apps: Study of Reliability and Validity. Canadian journal of occupational therapy Revue canadienne d'ergotherapie. 2022;89(3):326-38. doi: [https://dx.doi.org/10.1177/00084174221085451](https://www.google.com/search?q=https://dx.doi.org/10.1177/00084174221085451).
60. Scripts E. Digital support personalized for your needs. Evernorth; 2020 [cited 2024 3 October]; Available from: <https://www.express-scripts.com/partner/healthsolutions>.
61. Artsenfederatie K. Medical App Checker: A Guide to Assessing Mobile Medical Apps. 2019.
62. Coravos A, Doerr M, Goldsack J, Manta C, Shervey M, Woods B, et al. Modernizing and designing evaluation frameworks for connected sensor technologies in medicine. npj Digital Medicine. 2020 2020-03-13;3(1). doi: 10.1038/s41746-020-0237-3.
63. Denecke K, Warren J. How to Evaluate Health Applications with Conversational User Interface? Studies in health technology and informatics. 2020;270:976-80. doi: [https://dx.doi.org/10.3233/SHTI200307](https://www.google.com/search?q=https://dx.doi.org/10.3233/SHTI200307).
64. Goldsack JC, Coravos A, Bakker JP, Bent B, Dowling AV, Fitzer-Attas C, et al. Verification, analytical validation, and clinical validation (V3): the foundation of determining fit-for-purpose for Biometric Monitoring Technologies (BioMeTs). npj digital Medicine. 2020;3(1):55.
65. Shoemaker SJ, Wolf MS, Brach C. Development of the Patient Education Materials Assessment Tool (PEMAT): a new measure of understandability and actionability for print and audiovisual patient information. Patient education and counseling. 2014;96(3):395-403.
66. Burton JC, Regala S, Williams D, Desai A, He H, Aalami O, et al. A Comparative Utility Score for Digital Health Tools. Journal of Medical Systems. 2022;46(6):34.
67. Gladman T, Tylee G, Gallagher S, Mair J, Rennie SC, Grainger R. A Tool for Rating the Value of Health Education Mobile Apps to Enhance Student Learning (MARuL): Development and Usability Study. JMIR mHealth and uHealth. 2020;8(7):e18015. PMID: 632499109. doi: <https://dx.doi.org/10.2196/18015>.
68. Pearson SD, Singh P, Beaudoin F, Campbell J, Schapiro L, Emond SK, et al. Institute for Clinical and Economic Review - Peterson Health Technology Institute value assessment framework for digital health technologies. Journal of Comparative Effectiveness Research. 2023;12(12):e230154. PMID: 2026864577. doi: [https://dx.doi.org/10.57264/cer-2023-0154](https://www.google.com/search?q=https://dx.doi.org/10.57264/cer-2023-0154).
69. Scott IA, Scuffham P, Gupta D, Harch TM, Borchi J, Richards B. Going digital: a narrative overview of the effects, quality and utility of mobile apps in chronic disease self-management. Australian health review : a publication of the Australian Hospital Association. 2020;44(1):62-82. PMID: 631256494. doi: [https://dx.doi.org/10.1071/AH18064](https://www.google.com/search?q=https://dx.doi.org/10.1071/AH18064).
70. Miller CJ, Wiltsey-Stirman S, Baumann AA. Iterative Decision-making for Evaluation of Adaptations (IDEA): A decision tree for balancing adaptation, fidelity, and intervention impact. Journal of Community Psychology. 2020 2020/05/01;48(4):1163-77. doi: <https://doi.org/10.1002/jcop.22279>.
71. iMedicalApps. Medpage Today; 2020 [cited 2024 6 November]; Available from: <https://www.imedicalapps.com/>.
72. Association AP. The App Evaluation Model. American Psychiatric Association; 2021 [cited 2024 10 October]; Available from: <https://www.psychiatry.org/psychiatrists/practice/mental-health-apps/the-app-evaluation-model>.
73. Digital N. Digital Assessment Questions v2.2. Healthify; 2019 [cited 2024 6 October]; Available from: <https://healthify.nz/assets/Apps-library/WP12-app-checkers-NHS-DAQ-2.2.pdf>.
74. Abts NA, McNicol SA, Branaghan RJ. Guidelines for Mobile Health Applications. Proceedings of the International Symposium on Human Factors and Ergonomics in Health Care. 2019 2019/09/01;8(1):197-200. doi: 10.1177/2327857919081050.
75. Torous J, Andersson G, Bertagnoli A, Christensen H, Cuijpers P, Firth J, et al. Towards a consensus around standards for smartphone apps and digital mental health. World psychiatry. 2019;18(1):97.
76. Haverinen J, Keränen N, Falkenbach P, Maijala A, Kolehmainen T, Reponen J. Digi-HTA: Health technology assessment framework for digital healthcare services. Finnish Journal of eHealth and eWelfare. 2019;11(4):326-41.
77. Mathews SC, McShea MJ, Hanley CL, Ravitz A, Labrique AB, Cohen AB. Digital health: a path to validation. npj Digital Medicine. 2019 2019/05/13;2(1):38. doi: 10.1038/s41746-019-0111-3.
78. Kowatsch T, Otto L, Harperink S, Cotti A, Schlieter H. A design and evaluation framework for digital health interventions. 2019;61(5-6):253-63. doi: doi:10.1515/itit-2019-0019.
79. Henson P, David G, Albright K, Torous J. Deriving a practical framework for the evaluation of health apps. The Lancet Digital health. 2019;1(2):e52-e4. PMID: 634109476. doi: [https://dx.doi.org/10.1016/S2589-7500%2819%2930013-5](https://www.google.com/search?q=https://dx.doi.org/10.1016/S2589-7500%252819%252930013-5).
80. Wykes T, Schueller S. Why reviewing apps is not enough: transparency for trust (T4T) principles of responsible health app marketplaces. Journal of medical Internet research. 2019;21(5):e12390.
81. Hoffman L, Benedetto E, Huang H, Grossman E, Kaluma D, Mann Z, et al. Augmenting Mental Health in Primary Care: A 1-Year Study of Deploying Smartphone Apps in a Multi-site Primary Care/Behavioral Health Integration Program. Frontiers in psychiatry. 2019;10:94. doi: <https://dx.doi.org/10.3389/fpsyt.2019.00094>.
82. van Haasteren A, Gille F, Fadda M, Vayena E. Development of the mHealth App Trustworthiness checklist. Digital health. 2019;5:2055207619886463.
83. Jeminiwa RN, Hohmann NS, Fox BI. Developing a Theoretical Framework for Evaluating the Quality of mHealth Apps for Adolescent Users: A Systematic Review. The journal of pediatric pharmacology and therapeutics : JPPT : the official journal of PPAG. 2019;24(4):254-69. doi: <https://dx.doi.org/10.5863/1551-6776-24.4.254>.
84. McKay FH, Slykerman S, Dunn M. The App Behavior Change Scale: Creation of a Scale to Assess the Potential of Apps to Promote Behavior Change. JMIR mHealth and uHealth. 2019;7(1):e11130. doi: <https://dx.doi.org/10.2196/11130>.
85. Guan Z, Sun L, Xiao Q, Wang Y. Constructing an assessment framework for the quality of asthma smartphone applications. BMC medical informatics and decision making. 2019;19(1):192. doi: <https://dx.doi.org/10.1186/s12911-019-0923-8>.
86. Mummah SA, Robinson TN, King AC, Gardner CD, Sutton S. IDEAS (Integrate, Design, Assess, and Share): a framework and toolkit of strategies for the development of more effective digital interventions to change health behavior. Journal of medical Internet research. 2016;18(12):e317.
87. Albrecht U-V, Malinka C, Long S, Raupach T, Hasenfuß G, von Jan U. Quality principles of app description texts and their significance in deciding to use health apps as assessed by medical students: survey study. JMIR mHealth and uHealth. 2019;7(2):e13375.
88. Biggs JS, Willcocks A, Burger M, Makeham MAB. Digital health benefits evaluation frameworks: building the evidence to support Australia's National Digital Health Strategy. Medical Journal of Australia. 2019;210:S9-S11.
89. Rahal RM, Mercer J, Kuziemsky C, Yaya S. Primary care physicians’ experience using advanced electronic medical record features to support chronic disease prevention and management: qualitative study. JMIR medical informatics. 2019;7(4):e13318.
90. World Health O. WHO guideline: recommendations on digital interventions for health system strengthening: web supplement 2: summary of findings and GRADE tables. World Health Organization, 2019.
91. Unsworth H, Dillon B, Collinson L, Powell H, Salmon M, Oladapo T, et al. The NICE evidence standards framework for digital health and care technologies–developing and maintaining an innovative evidence framework with global impact. Digital health. 2021;7:20552076211018617.
92. Torous JB, Chan SR, Gipson SY-MT, Kim JW, Nguyen T-Q, Luo J, et al. A Hierarchical Framework for Evaluation and Informed Decision Making Regarding Smartphone Apps for Clinical Care. Psychiatric services (Washington, DC). 2018;69(5):498-500. doi: <https://dx.doi.org/10.1176/appi.ps.201700423>.
93. Nouri R, Kalhori NRS, Ghazisaeedi M, Marchand G, Yasini M. Criteria for assessing the quality of mHealth apps: a systematic review. Journal of the American Medical Informatics Association. 2018;25(8):1089-98. doi: 10.1093/jamia/ocy050.
94. Armstrong CM, Edwards-Stewart A, Ciulla RP, Bush NE, Cooper DC, Kinn JT, et al. Department of Defense mobile health practice guide. Military Health System. 2017.
95. Canada MHCo. Toolkit for e-Mental Health Implementation. 2021 [cited 2024 2 November]; Available from: <https://mentalhealthcommission.ca/wp-content/uploads/2021/05/E_Mental_Health_Implementation_Toolkit_2018_eng.pdf>.
96. Zelmer J, van Hoof K, Notarianni M, van Mierlo T, Schellenberg M, Tannenbaum C. An assessment framework for e-mental health apps in Canada: results of a modified Delphi process. JMIR mHealth and uHealth. 2018;6(7):e10016.
97. Wyatt JC. How can clinicians, specialty societies and others evaluate and improve the quality of apps for patient use? BMC medicine. 2018;16:1-10.
98. Kaltheuner M, Drossel D, Heinemann L. DiaDigital Apps: Evaluation of Smartphone Apps Using a Quality Rating Methodology for Use by Patients and Diabetologists in Germany. Journal of diabetes science and technology. 2019;13(4):756-62. doi: [https://dx.doi.org/10.1177/1932296818803098](https://www.google.com/search?q=https://dx.doi.org/10.1177/1932296818803098).
99. Masud A, Shafi S, Rao BK. Mobile medical apps for patient education: a graded review of available dermatology apps. Cutis. 2018;101(2):141-4.
100. Jisha RC, Krishnan R, Vikraman V, editors. Mobile applications recommendation based on user ratings and permissions. 2018: IEEE.
101. Habib SM, Alexopoulos N, Islam MM, Heider J, Marsh S, Müehlhäeuser M, editors. Trust4App: automating trustworthiness assessment of mobile applications. 2018: IEEE.
102. Farnia T, Jaulent MC, Steichen O. Evaluation Criteria of Noninvasive Telemonitoring for Patients With Heart Failure: Systematic Review. J Med Internet Res. 2018 Jan 16;20(1):e16. PMID: 29339348. doi: 10.2196/jmir.7873.
103. Wight D, Wimbush E, Jepson R, Doi L. Six steps in quality intervention development (6SQuID). J Epidemiol Community Health. 2016;70(5):520-5.
104. Sadegh SS, Khakshour Saadat P, Sepehri MM, Assadi V. A framework for m-health service development and success evaluation. International journal of medical informatics. 2018;112:123-30. doi: [https://dx.doi.org/10.1016/j.ijmedinf.2018.01.003](https://www.google.com/search?q=https://dx.doi.org/10.1016/j.ijmedinf.2018.01.003).
105. Fiks AG, Fleisher L, Berrigan L, Sykes E, Mayne SL, Gruver R, et al. Usability, Acceptability, and Impact of a Pediatric Teledermatology Mobile Health Application. Telemedicine and e-Health. 2017 2018/03/01;24(3):236-45. doi: 10.1089/tmj.2017.0075.
106. Ali EE, Teo AKS, Goh SXL, Chew L, Yap KY-L. MedAd-AppQ: A quality assessment tool for medication adherence apps on iOS and android platforms. Research in social & administrative pharmacy : RSAP. 2018;14(12):1125-33. doi: [https://dx.doi.org/10.1016/j.sapharm.2018.01.006](https://www.google.com/search?q=https://dx.doi.org/10.1016/j.sapharm.2018.01.006).
107. Betton V, Craven M, Davies B, Martin J, Nelissen N, Ridout A, et al. Framework for the effectiveness evaluation of mobile (mental) health tools. 2017.
108. Baumel A, Faber K, Mathur N, Kane JM, Muench F. Enlight: A Comprehensive Quality and Therapeutic Potential Evaluation Tool for Mobile and Web-Based eHealth Interventions. Journal of medical Internet research. 2017;19(3):e82. doi: <https://dx.doi.org/10.2196/jmir.7270>.
109. Leigh S, Ouyang J, Mimnagh C. Effective? Engaging? Secure? Applying the ORCHA-24 framework to evaluate apps for chronic insomnia disorder. Evidence-based mental health. 2017;20(4):e20. doi: [https://dx.doi.org/10.1136/eb-2017-102751](https://www.google.com/search?q=https://dx.doi.org/10.1136/eb-2017-102751).
110. Royston G, editor. Rapid Methods to Assess the Potential Impact of Digital Health Interventions, and their Application to Low Resource Settings. 2017.
111. Gibbs J, Gkatzidou V, Tickle L, Manning SR, Tilakkumar T, Hone K, et al. ‘Can you recommend any good STI apps?’ A review of content, accuracy and comprehensiveness of current mobile medical applications for STIs and related genital infections. Sexually Transmitted Infections. 2017;93(4):234. doi: 10.1136/sextrans-2016-052690.
112. Hoppe CD, Cade JE, Carter M. An evaluation of diabetes targeted apps for Android smartphone in relation to behaviour change techniques. Journal of human nutrition and dietetics. 2017;30(3):326-38.
113. Wealth S. Our approach: Social wealth. 2016 [cited 2024 8 November]; Available from: [http://www.happtique.com/](https://www.google.com/search?q=http://www.happtique.com/).
114. Veldsman A, Greunen Dv, editors. Comparative usability evaluation of a mobile health app. 2017 IST-Africa Week Conference (IST-Africa); 2017 30 May-2 June 2017.
115. Shaia KL, Farag S, Chyjek K, Knopman J, Chen KT. An evaluation of mobile applications for reproductive endocrinology and infertility providers. Telemedicine and e-Health. 2017;23(3):254-8.
116. Greenhalgh T, Wherton J, Papoutsi C, Lynch J, Hughes G, A'Court C, et al. Beyond Adoption: A New Framework for Theorizing and Evaluating Nonadoption, Abandonment, and Challenges to the Scale-Up, Spread, and Sustainability of Health and Care Technologies. J Med Internet Res. 2017 Nov 1;19(11):e367. PMID: 29092808. doi: 10.2196/jmir.8775.
117. DiFilippo KN, Huang W, Chapman-Novakofski KM. A New Tool for Nutrition App Quality Evaluation (AQEL): Development, Validation, and Reliability Testing. JMIR mHealth and uHealth. 2017;5(10):e163. doi: <https://dx.doi.org/10.2196/mhealth.7441>.
118. Xie B, Su Z, Zhang W, Cai R. Chinese Cardiovascular Disease Mobile Apps' Information Types, Information Quality, and Interactive Functions for Self-Management: Systematic Review. JMIR mHealth and uHealth. 2017;5(12):e195. doi: <https://dx.doi.org/10.2196/mhealth.8549>.
119. Høstgaard AMB, Bertelsen P, Nøhr C. Constructive eHealth evaluation: lessons from evaluation of EHR development in 4 Danish hospitals. BMC medical informatics and decision making. 2017;17:1-15.
120. Ferguson C, Jackson D. Selecting, appraising, recommending and using mobile applications (apps) in nursing. Journal of clinical nursing. 2017;26(21-22):3253-5. doi: [https://dx.doi.org/10.1111/jocn.13834](https://www.google.com/search?q=https://dx.doi.org/10.1111/jocn.13834).
121. Cook VE, Ellis AK, Hildebrand KJ. Mobile health applications in clinical practice: pearls, pitfalls, and key considerations. Annals of Allergy, Asthma & Immunology. 2016 2016/08/01/;117(2):143-9. doi: <https://doi.org/10.1016/j.anai.2016.01.012>.
122. Basilico A, Marceglia S, Bonacina S, Pinciroli F. Advising patients on selecting trustful apps for diabetes self-care. Computers in Biology and Medicine. 2016;71:86-96.
123. Murray E, Hekler EB, Andersson G, Collins LM, Doherty A, Hollis C, et al. Evaluating digital health interventions: key questions and approaches. Elsevier; 2016. p. 843-51.
124. Agarwal S, LeFevre AE, Lee J, L’engle K, Mehl G, Sinha C, et al. Guidelines for reporting of health interventions using mobile phones: mobile health (mHealth) evidence reporting and assessment (mERA) checklist. bmj. 2016;352.
125. de Santé HA. Good practice guidelines on health apps and smart devices (mobile health or mHealth). Haute Autorité de Santé (HAS). 2016.
126. Maheu MM, Nicolucci V, Pulier ML, Wall KM, Frye TJ, Hudlicka E. The interactive mobile app review toolkit (IMART): a clinical practice-oriented system. Journal of Technology in Behavioral Science. 2016;1:3-15.
127. Drincic A, Prahalad P, Greenwood D, Klonoff DC. Evidence-based mobile medical applications in diabetes. Endocrinology and Metabolism Clinics. 2016;45(4):943-65.
128. McMillan B, Hickey E, Patel MG, Mitchell C. Quality assessment of a sample of mobile app-based health behavior change interventions using a tool based on the National Institute of Health and Care Excellence behavior change guidance. Patient education and counseling. 2016;99(3):429-35.
129. Xiao Q, Wang Y, Sun L, Lu S, Wu Y. Current status and quality assessment of cardiovascular diseases related smartphone apps in China. Nursing Informatics 2016: IOS Press; 2016. p. 1030-1.
130. Yasini M, Beranger J, Desmarais P, Perez L, Marchand G. mHealth quality: a process to seal the qualified mobile health apps. Exploring complexity in health: An interdisciplinary systems approach: IOS Press; 2016. p. 205-9.
131. Stoyanov SR, Hides L, Kavanagh DJ, Wilson H. Development and validation of the user version of the Mobile Application Rating Scale (uMARS). JMIR mHealth and uHealth. 2016;4(2):e5849.
132. McNiel P, McArthur EC. Evaluating health mobile apps: information literacy in undergraduate and graduate nursing courses. Journal of Nursing Education. 2016;55(8):480-.
133. Loy JS, Ali EE, Yap KY-L. Quality assessment of medical apps that target medication-related problems. Journal of managed care & specialty pharmacy. 2016;22(10):1124-40.
134. Anderson K, Burford O, Emmerton L. App chronic disease checklist: protocol to evaluate mobile apps for chronic disease self-management. JMIR research protocols. 2016;5(4):e6194.
135. Iribarren SJ, Schnall R, Stone PW, Carballo-Diéguez A. Smartphone applications to support tuberculosis prevention and treatment: review and evaluation. JMIR mHealth and uHealth. 2016;4(2):e5022.
136. Ferrero-Alvarez-Rementeria J, Santana-Lopez V, Escobar-Ubreva A, Vazquez-Vazquez M. Quality and safety strategy for mobile health applications: a certification programme. Eur J ePractice. 2013;21:14-26.
137. Grundy QH, Wang Z, Bero LA. Challenges in Assessing Mobile Health App Quality: A Systematic Review of Prevalent and Innovative Methods. American Journal of Preventive Medicine. 2016 2016/12/01/;51(6):1051-9. doi: <https://doi.org/10.1016/j.amepre.2016.07.009>.
138. Portelli P, Eldred C. A quality review of smartphone applications for the management of pain. British Journal of Pain. 2016 2016/08/01;10(3):135-40. doi: 10.1177/2049463716638700.
139. Powell AC, Torous J, Chan S, Raynor GS, Shwarts E, Shanahan M, et al. Interrater Reliability of mHealth App Rating Measures: Analysis of Top Depression and Smoking Cessation Apps. JMIR mHealth uHealth. 2016 2016/02/10;4(1):e15. doi: 10.2196/mhealth.5176.
140. Singh K, Drouin K, Newmark LP, Rozenblum R, Lee J, Landman A, et al. Developing a framework for evaluating the patient engagement, quality, and safety of mobile health applications. Issue Brief (Commonw Fund). 2016;5(1):11.
141. Association AM. AMA adopts principles to promote safe, effective mHealth applications. ama.org: AMA; 2016.
142. Eldredge LKB, Markham CM, Ruiter RAC, Fernández ME, Kok G, Parcel GS. Planning health promotion programs: an intervention mapping approach: John Wiley & Sons; 2016. ISBN: 111903549X.
143. Schnall R, Rojas M, Bakken S, Brown W, Carballo-Dieguez A, Carry M, et al. A user-centered model for designing consumer mobile health (mHealth) applications (apps). Journal of biomedical informatics. 2016;60:243-51.
144. Kruse CS, Bouffard S, Dougherty M, Parro JS. Telemedicine use in rural Native American communities in the era of the ACA: a systematic literature review. Journal of medical systems. 2016;40:1-9.
145. Meriam L. Evaluating information–applying the CRAAP test. California State University Chico, CA; 2010.
146. CapraŞ RD, BolboacĂ SD. An evaluation of free medical applications for android smartphones. Applied Medical Informatics. 2016;38(3-4):117-32.
147. World Health O. Monitoring and evaluating digital health interventions: a practical guide to conducting research and assessment. 2016.
148. Sedrakyan A, Campbell B, Merino JG, Kuntz R, Hirst A, McCulloch P. IDEAL-D: a rational framework for evaluating and regulating the use of medical devices. Bmj. 2016;353.
149. McNiel P, McArthur Erin C. Evaluating Health Mobile Apps: Information Literacy in Undergraduate and Graduate Nursing Courses. Journal of Nursing Education. 2016 2016/08/01;55(8):480-. doi: 10.3928/01484834-20160715-12.
150. Stoyanov SR, Hides L, Kavanagh DJ, Zelenko O, Tjondronegoro D, Mani M. Mobile app rating scale: A new tool for assessing the quality of health mobile apps. JMIR mHealth and uHealth. 2015;3(1):1-9. doi: <https://dx.doi.org/10.2196/mhealth.3422>.
151. Institute BS. PAS 277:2015 Health and wellness apps - quality criteria across the life cycle - code of practice, 2015. 2015.
152. Wyatt JC, Thimbleby H, Rastall P, Hoogewerf J, Wooldridge D, Williams J. What makes a good clinical app? Introducing the RCP Health Informatics Unit checklist. Clinical Medicine. 2015;15(6):519-21.
153. Chan S, Torous J, Hinton L, Yellowlees P. Towards a framework for evaluating mobile mental health apps. Telemedicine and e-Health. 2015;21(12):1038-41.
154. Zapata BC, Fernández-Alemán JL, Idri A, Toval A. Empirical studies on usability of mHealth apps: a systematic literature review. Journal of medical systems. 2015;39:1-19.
155. Jin M, Kim J. Development and evaluation of an evaluation tool for healthcare smartphone applications. Telemedicine and e-Health. 2015;21(10):831-7.
156. Martínez-Pérez B, de la Torre-Díez I, López-Coronado M. Experiences and results of applying tools for assessing the quality of a mHealth app named Heartkeeper. Journal of medical systems. 2015;39:1-6.
157. Taki S, Campbell KJ, Russell CG, Elliott R, Laws R, Denney-Wilson E. Infant feeding websites and apps: a systematic assessment of quality and content. Interactive journal of medical research. 2015;4(3):e4323.
158. Scott K, Richards D, Adhikari R. A review and comparative analysis of security risks and safety measures of mobile health apps. Australasian Journal of Information Systems. 2015;19:1-18.
159. BinDhim NF, Hawkey A, Trevena L. A systematic review of quality assessment methods for smartphone health apps. Telemedicine and e-Health. 2015;21(2):97-104.
160. Brooks GC, Vittinghoff E, Iyer S, Tandon D, Kuhar P, Madsen KA, et al. Accuracy and usability of a self-administered 6-minute walk test smartphone application. Circulation: Heart Failure. 2015;8(5):905-13.
161. Fairburn CG, Rothwell ER. Apps and eating disorders: A systematic clinical appraisal. International Journal of Eating Disorders. 2015 2015/11/01;48(7):1038-46. doi: <https://doi.org/10.1002/eat.22398>.
162. Gautham M, Iyengar MS, Johnson CW. Mobile phone–based clinical guidance for rural health providers in India. Health Informatics Journal. 2014 2015/12/01;21(4):253-66. doi: 10.1177/1460458214523153.
163. Kassianos AP, Emery JD, Murchie P, Walter FM. Smartphone applications for melanoma detection by community, patient and generalist clinician users: a review. British Journal of Dermatology. 2015;172(6):1507-18.
164. Lalloo C, Jibb LA, Rivera J, Agarwal A, Stinson JN. “There’sa pain app for that”: review of patient-targeted smartphone applications for pain management. The Clinical journal of pain. 2015;31(6):557-63.
165. Lee H, Sullivan SJ, Schneiders AG, Ahmed OH, Balasundaram AP, Williams D, et al. Smartphone and tablet apps for concussion road warriors (team clinicians): a systematic review for practical users. British Journal of Sports Medicine. 2015;49(8):499. doi: 10.1136/bjsports-2013-092930.
166. Schnall R, Mosley JP, Iribarren SJ, Bakken S, Carballo-Diéguez A, Brown Iii W. Comparison of a User-Centered Design, Self-Management App to Existing mHealth Apps for Persons Living With HIV. JMIR mHealth uHealth. 2015 2015/09/18;3(3):e91. doi: 10.2196/mhealth.4882.
167. Shen N, Levitan M-J, Johnson A, Bender JL, Hamilton-Page M, Jadad AR, et al. Finding a Depression App: A Review and Content Analysis of the Depression App Marketplace. JMIR mHealth uHealth. 2015 2015/02/16;3(1):e16. doi: 10.2196/mhealth.3713.
168. Golden A, Krauskopf P. Systematic evaluation of mobile apps. The Journal for Nurse Practitioners. 2016;12(1):e27-e8.
169. Mohr DC, Schueller SM, Riley WT, Brown CH, Cuijpers P, Duan N, et al. Trials of intervention principles: evaluation methods for evolving behavioral intervention technologies. Journal of medical Internet research. 2015;17(7):e166.
170. Salisbury C, Thomas C, O'Cathain A, Rogers A, Pope C, Yardley L, et al. TElehealth in CHronic disease: mixed-methods study to develop the TECH conceptual model for intervention design and evaluation. BMJ open. 2015;5(2):e006448.
171. Butcher R, MacKinnon M, Gadd K, LeBlanc-Duchin D. Development and Examination of a Rubric for Evaluating Point-of-Care Medical Applications for Mobile Devices. Medical Reference Services Quarterly. 2015 2015/01/02;34(1):75-87. doi: 10.1080/02763869.2015.986794.
172. Boudreaux ED, Waring ME, Hayes RB, Sadasivam RS, Mullen S, Pagoto S. Evaluating and selecting mobile health apps: strategies for healthcare providers and healthcare organizations. Translational behavioral medicine. 2014;4(4):363-71.
173. Aungst TD, Clauson KA, Misra S, Lewis TL, Husain I. How to identify, assess and utilise mobile medical applications in clinical practice. International journal of clinical practice. 2014;68(2):155-62.
174. Hanrahan C, Aungst TD, Cole S. Evaluating mobile medical applications: American Society of Health-System Pharmacists; 2014. ISBN: 1585284580.
175. Reynoldson C, Stones C, Allsop M, Gardner P, Bennett MI, Closs SJ, et al. Assessing the quality and usability of smartphone apps for pain self-management. Pain medicine. 2014;15(6):898-909.
176. Cruz Zapata B, Hernandez Ninirola A, Idri A, Fernández-Alemán JL, Toval A. Mobile PHRs compliance with Android and iOS usability guidelines. Journal of medical systems. 2014;38:1-16.
177. Arnhold M, Quade M, Kirch W. Mobile applications for diabetics: a systematic review and expert-based usability evaluation considering the special requirements of diabetes patients age 50 years or older. Journal of medical Internet research. 2014;16(4):e104.
178. Mobasheri MH, Johnston M, King D, Leff D, Thiruchelvam P, Darzi A. Smartphone breast applications – What's the evidence? The Breast. 2014 2014/10/01/;23(5):683-9. doi: <https://doi.org/10.1016/j.breast.2014.07.006>.
179. Robustillo Cortés MdlA, Cantudo Cuenca MR, Morillo Verdugo R, Calvo Cidoncha E. High Quantity But Limited Quality in Healthcare Applications Intended for HIV-Infected Patients. Telemedicine and e-Health. 2014 2014/08/01;20(8):729-35. doi: 10.1089/tmj.2013.0262.
180. Shah N, Jonassaint J, De Castro L. Patients Welcome the Sickle Cell Disease Mobile Application to Record Symptoms via Technology (SMART). Hemoglobin. 2014 2014/04/01;38(2):99-103. doi: 10.3109/03630269.2014.880716.
181. Jang-Jaccard J, Nepal S, Alem L, Li J. Barriers for delivering telehealth in rural Australia: a review based on Australian trials and studies. Telemedicine and e-Health. 2014;20(5):496-504.
182. Simpson SG, Reid CL. Therapeutic alliance in videoconferencing psychotherapy: A review. Australian Journal of Rural Health. 2014;22(6):280-99.
183. Price M, Lau F. The clinical adoption meta-model: a temporal meta-model describing the clinical adoption of health information systems. BMC Medical Informatics and Decision Making. 2014 2014/05/29;14(1):43. doi: 10.1186/1472-6947-14-43.
184. PsyberGuide OM. Find Apps for Workplaces. One; 2013 [cited 2024 1 Oct]; Available from: <https://onemindpsyberguide.org/guide/apps-workplace/>.
185. Albrecht U-V, Von Jan U, Pramann O. Standard reporting for medical apps. Informatics, management and technology in healthcare. 2013:201-3.
186. Beatty AL, Fukuoka Y, Whooley MA. Using mobile technology for cardiac rehabilitation: a review and framework for development and evaluation. Journal of the American Heart Association. 2013;2(6):e000568.
187. Khoja S, Durrani H, Scott RE, Sajwani A, Piryani U. Conceptual framework for development of comprehensive e-health evaluation tool. Telemedicine and e-Health. 2013;19(1):48-53.
188. Brown Iii W, Yen P-Y, Rojas M, Schnall R. Assessment of the Health IT Usability Evaluation Model (Health-ITUEM) for evaluating mobile health (mHealth) technology. Journal of biomedical informatics. 2013;46(6):1080-7.
189. Martínez-Pérez B, de la Torre-Díez I, Candelas-Plasencia S, López-Coronado M. Development and evaluation of tools for measuring the quality of experience (QoE) in mHealth applications. Journal of medical systems. 2013;37:1-8.
190. Informatics I. Patient Apps for Improved Healthcare: From Novelty to Mainstream. Report by the IMS Institute for Healthcare Informatics. 2013.
191. Murfin M. Know Your Apps: An Evidence-Based Approach to Evaluation of Mobile Clinical Applications. The Journal of Physician Assistant Education. 2013;24(3).
192. Pandey A, Hasan S, Dubey D, Sarangi S. Smartphone Apps as a Source of Cancer Information: Changing Trends in Health Information-Seeking Behavior. Journal of Cancer Education. 2013 2013/03/01;28(1):138-42. doi: 10.1007/s13187-012-0446-9.
193. Force FSaNNSaBCT. Designing for Behavior Change For Agriculture, Natural Resource Management, Health and Nutrition. Washington, DC: Technical and Operational Performance Support (TOPS) Program.; 2013.
194. Hage E, Roo JP, van Offenbeek MAG, Boonstra A. Implementation factors and their effect on e-Health service adoption in rural communities: a systematic literature review. BMC health services research. 2013;13:1-16.
195. Fitzner K, Moss G. Telehealth—an effective delivery method for diabetes self-management education? Population health management. 2013;16(3):169-77.
196. Gros DF, Morland LA, Greene CJ, Acierno R, Strachan M, Egede LE, et al. Delivery of evidence-based psychotherapy via video telehealth. Journal of Psychopathology and Behavioral Assessment. 2013;35:506-21.
197. Michie S, Richardson M, Johnston M, Abraham C, Francis J, Hardeman W, et al. The behavior change technique taxonomy (v1) of 93 hierarchically clustered techniques: building an international consensus for the reporting of behavior change interventions. Annals of behavioral medicine. 2013;46(1):81-95.
198. Kidholm K, Ekeland AG, Jensen LK, Rasmussen J, Pedersen CD, Bowes A, et al. A model for assessment of telemedicine applications: mast. International journal of technology assessment in health care. 2012;28(1):44-51.
199. Huckvale K, Car M, Morrison C, Car J. Apps for asthma self-management: a systematic assessment of content and tools. BMC medicine. 2012;10:1-11.
200. Demidowich AP, Lu K, Tamler R, Bloomgarden Z. An evaluation of diabetes self-management applications for Android smartphones. Journal of Telemedicine and Telecare. 2012 2012/06/01;18(4):235-8. doi: 10.1258/jtt.2012.111002.
201. Group mAUW. Selecting a mobile app: Evaluating the usability of medical applications Health Care Information Management Systems Society; 2012 [cited 2024 17 October]; Available from: http:/​/www.​himss.org/​files/​HIMSSorg/​content/​files/SelectingMobileApp_EvaluatingUsabilityMedicalApplications.​pdf.
202. Walsworth DT. Medical apps: making your mobile device a medical device. Family Practice Management. 2012;19(3):10-3.
203. Saliba V, Legido-Quigley H, Hallik R, Aaviksoo A, Car J, McKee M. Telemedicine across borders: a systematic review of factors that hinder or support implementation. International journal of medical informatics. 2012;81(12):793-809.
204. Govender SM, Mars M. The use of telehealth services to facilitate audiological management for children: A scoping review and content analysis. Journal of Telemedicine and Telecare. 2017;23(3):392-401.
205. Kharrazi H, Chisholm R, VanNasdale D, Thompson B. Mobile personal health records: An evaluation of features and functionality. International Journal of Medical Informatics. 2012 2012/09/01/;81(9):579-93. doi: <https://doi.org/10.1016/j.ijmedinf.2012.04.007>.
206. Eysenbach G, Consort EG. CONSORT-EHEALTH: improving and standardizing evaluation reports of Web-based and mobile health interventions. Journal of medical Internet research. 2011;13(4):e1923.
207. Gan KO, Allman‐Farinelli M. A scientific audit of smartphone applications for the management of obesity. Australian and New Zealand journal of public health. 2011;35(3):293-4.
208. Chomutare T, Fernandez-Luque L, Årsand E, Hartvigsen G. Features of mobile diabetes applications: review of the literature and analysis of current applications compared against evidence-based guidelines. Journal of medical Internet research. 2011;13(3):e1874.
209. Michie S, Van Stralen MM, West R. The behaviour change wheel: a new method for characterising and designing behaviour change interventions. Implementation science. 2011;6:1-12.
210. Michie S, Ashford S, Sniehotta FF, Dombrowski SU, Bishop A, French DP. A refined taxonomy of behaviour change techniques to help people change their physical activity and healthy eating behaviours: the CALO-RE taxonomy. Psychology & health. 2011;26(11):1479-98.
211. Day K, Gu Y, Warren J, White S, Pollock M. National eReferrals Evaluation: Findings for the Northland District Health Board. 2011.
212. Proctor E, Silmere H, Raghavan R, Hovmand P, Aarons G, Bunger A, et al. Outcomes for implementation research: conceptual distinctions, measurement challenges, and research agenda. Administration and policy in mental health and mental health services research. 2011;38:65-76.
213. Schulze K, Krömker H, editors. A framework to measure user experience of interactive online products. 2010.
214. Kotz D, Avancha S, Baxi A, editors. A privacy framework for mobile health and home-care systems. 2009.
215. Daraz L, MacDermid JC, Wilkins S, Gibson J, Shaw L, editors. Health information from the web—assessing its quality: a KET intervention. 2009: IEEE.
216. Damschroder LJ, Aron DC, Keith RE, Kirsh SR, Alexander JA, Lowery JC. Fostering implementation of health services research findings into practice: a consolidated framework for advancing implementation science. Implementation science. 2009;4:1-15.
217. Catwell L, Sheikh A. Evaluating eHealth interventions: the need for continuous systemic evaluation. PLoS medicine. 2009;6(8):e1000126.
218. Lau F, Price M. Clinical adoption framework. Handbook of eHealth Evaluation: An Evidence-based Approach [Internet]: University of Victoria; 2017.
219. Proctor EK, Landsverk J, Aarons G, Chambers D, Glisson C, Mittman B. Implementation research in mental health services: an emerging science with conceptual, methodological, and training challenges. Administration and Policy in Mental Health and Mental Health Services Research. 2009;36:24-34.
220. May CR, Mair F, Finch T, MacFarlane A, Dowrick C, Treweek S, et al. Development of a theory of implementation and integration: Normalization Process Theory. Implementation Science. 2009 2009/05/21;4(1):29. doi: 10.1186/1748-5908-4-29.
221. Abraham C, Michie S. A taxonomy of behavior change techniques used in interventions. Health psychology. 2008;27(3):379.
222. Yusof MM, Kuljis J, Papazafeiropoulou A, Stergioulas LK. An evaluation framework for Health Information Systems: human, organization and technology-fit factors (HOT-fit). International journal of medical informatics. 2008;77(6):386-98.
223. Lampe K, Pasternack I. HTA Core Model for Diagnostic Technologies v 1.0. Work Package 4: The HTA Core Model. 2008.
224. Feldstein AC, Glasgow RE. A practical, robust implementation and sustainability model (PRISM) for integrating research findings into practice. The joint commission journal on quality and patient safety. 2008;34(4):228-43.
225. Petter S, DeLone W, McLean E. Measuring information systems success: models, dimensions, measures, and interrelationships. European Journal of Information Systems. 2008 2008/06/01;17(3):236-63. doi: 10.1057/ejis.2008.15.
226. Craig P, Dieppe P, Macintyre S, Michie S, Nazareth I, Petticrew M. Developing and evaluating complex interventions: the new Medical Research Council guidance. Bmj. 2008;337.
227. Collins LM, Murphy SA, Strecher V. The multiphase optimization strategy (MOST) and the sequential multiple assignment randomized trial (SMART): new methods for more potent eHealth interventions. American journal of preventive medicine. 2007;32(5):S112-S8.
228. Lau F, Hagens S, Muttitt S. A proposed benefits evaluation framework for health information systems in Canada. Healthcare Quarterly (Toronto, Ont). 2007;10(1):112-6.
229. Bright TJ, Bakken S, Johnson SB, editors. Heuristic evaluation of eNote: an electronic notes system. 2006: American Medical Informatics Association.
230. Lewiecki EM, Rudolph LA, Kiebzak GM, Chavez JR, Thorpe BM. Assessment of osteoporosis-website quality. Osteoporosis international. 2006;17:741-52.
231. Green LW. Health program planning: An educational and ecological approach. Mountain View. 2005.
232. Charnock D, Shepperd S. Learning to DISCERN online: applying an appraisal tool to health websites in a workshop setting. Health education research. 2004;19(4):440-6.
233. Moustakis V, Litos C, Dalivigas A, Tsironis L, editors. Website Quality Assessment Criteria. 2004.
234. Rycroft-Malone J. The PARIHS Framework—A Framework for Guiding the Implementation of Evidence-based Practice. Journal of Nursing Care Quality. 2004;19(4).
235. Kukafka R, Johnson SB, Linfante A, Allegrante JP. Grounding a new information technology implementation framework in behavioral science: a systematic analysis of the literature on IT use. Journal of biomedical informatics. 2003;36(3):218-27.
236. Hailey D. Toward transparency in health technology assessment: a checklist for HTA reports. International journal of technology assessment in health care. 2003;19(1):1-7.
237. Linnan L, Steckler A. Process evaluation for public health interventions and research. 2002.
238. Jeon E, Park H-A, Min YH, Kim H-Y. Analysis of the information quality of Korean obesity-management smartphone applications. Healthcare informatics research. 2014;20(1):23-9.
239. Abbott VP. Web page quality: can we measure it and what do we find? A report of exploratory findings. Journal of Public Health. 2000;22(2):191-7.
240. Campbell M, Fitzpatrick R, Haines A, Kinmonth AL, Sandercock P, Spiegelhalter D, et al. Framework for design and evaluation of complex interventions to improve health. Bmj. 2000;321(7262):694-6.
241. Glasgow RE, Vogt TM, Boles SM. Evaluating the public health impact of health promotion interventions: the RE-AIM framework. American journal of public health. 1999;89(9):1322-7.
242. Quality AfHRa. Assessing the Quality of Internet Health Information. US Department of Health and Human Services; 1999 [cited 2024 10 November]; Available from: <https://archive.ahrq.gov/research/data/infoqual.html>.
243. Jones J, editor. Development of a self-assessment method for patients to evaluate health information on the Internet. 1999: American Medical Informatics Association.
244. Boyer C, Selby M, Scherrer JR, Appel RD. The health on the net code of conduct for medical and health websites. Computers in biology and medicine. 1998;28(5):603-10.
245. de Zoysa I, Habicht J-P, Pelto G, Martines J. Research steps in the development and evaluation of public health interventions. Bulletin of the World Health Organization. 1998;76(2):127.
246. Bartholomew LK, Parcel GS, Kok G. Intervention Mapping: A Process for Developing Theory and Evidence-Based Health Education Programs. Health Education & Behavior. 1998 1998/10/01;25(5):545-63. doi: 10.1177/109019819802500502.
247. Silberg WM, Lundberg GD, Musacchio RA. Assessing, controlling, and assuring the quality of medical information on the Internet: Caveant lector et viewor—Let the reader and viewer beware. Jama. 1997;277(15):1244-5.
248. Seels BB, Richey RC. Instructional technology: The definition and domains of the field: Iap; 2012. ISBN: 161735905X.
249. CredibleMind. Empower Everyone’s Mental Wellbeing. Unknown [cited 2024 3 October]; Available from: <https://crediblemind.com/>.
